# Morphological characters of immature stages of Palaearctic species of *Cleopomiarus* and *Miarus* and their systematic value in Mecinini (Coleoptera, Curculionidae, Curculioninae)

**DOI:** 10.3897/zookeys.808.28172

**Published:** 2018-12-18

**Authors:** Jiří Skuhrovec, Rafał Gosik, Roberto Caldara, Ivo Toševski, Jacek Łętowski, Ewelina Szwaj

**Affiliations:** 1 Group Function of Invertebrate and Plant Biodiversity in Agro-Ecosystems, Crop Research Institute, Prague 6–Ruzyně, Czech Republic Crop Research Institute Prague Czech Republic; 2 Department of Zoology, Maria Curie-Skłodowska University, Akademicka 19, 20-033 Lublin, Poland Maria Curie-Skłodowska University Lublin Poland; 3 Center of Alpine Entomology, University of Milan, Via Celoria 2, 20133 Milan, Italy University of Milan Milan Italy; 4 CABI, Rue des Grillons 1, 2800 Delémont, Switzerland CABI Delémont Switzerland; 5 Institute for Plant Protection and Environment, Banatska 33, 11080 Zemun, Serbia Institute for Plant Protection and Environment Zemun Serbia; 6 University of Life Sciences in Lublin, ul. Akademicka 13, 20-950 Lublin, Poland University of Life Sciences in Lublin Lublin Poland

**Keywords:** biology, *
Cleopomiarus
*, distribution, mature larva, Mecinini, *
Miarus
*, morphology, pupa

## Abstract

The relationship between the genera *Cleopomiarus* and *Miarus* of Mecinini (Curculionidae, Curculioninae) was tested on the basis of morphological characters from the immature stages. The mature larvae of five *Cleopomiarus* species (*C.distinctus* (Boheman, 1845), *C.graminis* (Gyllenhal, 1813), *C.longirostris* (Gyllenhal, 1838), *C.medius* (Desbrochers des Loges, 1893), and *C.meridionalis* (H. Brisout de Barneville, 1863)), three *Miarus* species (*M.abnormis* Solari, 1947, *M.ajugae* (Herbst, 1795), and *M.campanulae* (Linnaeus, 1767)), and the pupae of four *Cleopomiarus* species (*C.distinctus*, *C.graminis*, *C.longirostris*, and *C.medius*) and two *Miarus* species (*M.abnormis* and *M.ajugae*) are described in detail for the first time. To confirm the taxonomic identification of some larvae, DNA COI barcode was obtained and compared with those of adults. The immature stages of the species herein studied were compared with those known from other genera in tribe Mecinini. It is suggested that *Miarus* and *Cleopomiarus* may be monophyletic based on several shared distinctive characters. Larvae of *Miarus* have a characteristic maxillary mala with six finger-like *dms* of two sizes (one or two *dms* very long and the rest of medium length), this feature being apparently unique among weevils. Other genus-specific character states are observed in the pupae, such as the length of setae on the head, rostrum and pronotum, including the number of *rs* on the rostrum, *ds* on pronotum, and finally the shape of the urogomphi. A key to the described larvae and pupae were respectively presented. New biological and distributional data on some species are reported.

## Introduction

The Mecinini is a tribe of the subfamily Curculioninae (Curculionidae) and comprises six genera: *Cleopomiarus* Pierce, 1919, *Gymnetron* Schoenherr, 1825, *Mecinus* Germar, 1821, *Miarus* Schoenherr, 1826, *Rhinumiarus* Caldara, 2001 and *Rhinusa* Stephens, 1829 (Caldara 2001; Caldara et al. 2014; Alonso-Zarazaga et al. 2017). Whereas *Rhinumiarus* is only found in South America, the other five genera are largely distributed in the Palaearctic region. Moreover, *Cleopomiarus* and *Gymnetron* are well known in the Afrotropical region, with many species, especially in South Africa (Caldara 2003, 2005). In the last twenty years, these genera were subjected to a careful taxonomic revision (not yet completed for *Rhinusa*) and a phylogenetic analysis based on adult morphology (Caldara 2001, 2003, 2005, 2007; Caldara et al. 2010, 2013; Caldara and Fogato 2013).

With regard to the biology, the larvae of Mecinini develop in roots, shoots, leaves and flowers, many of them causing the organs of the host plants to swell or develop into galls; moreover, some species of *Rhinusa* are inquilines in galls produced by other species of the same genus (Hoffmann 1958; Arzanov 2000; Caldara 2001, 2003, 2005, 2007; Korotyaev et al. 2005). The larvae are predominantly associated with the families Scrophulariaceae, Plantaginaceae, and Campanulaceae (sensu APG 2016). *Mecinus* species live on Plantaginaceae, while *Gymnetron* and *Rhinusa* species live on both Scrophulariaceae and Plantaginaceae, which are two closely related families placed together in the Order Lamiales (Olmstead et al. 2001; Albach et al. 2005; APG 2016). The Palaearctic species of *Gymnetron* live on *Veronica* (Caldara 2008), currently included in Plantaginaceae (Olmstead et al. 2001; Albach et al. 2005), while those in the Afrotropical region, where Plantaginaceae are poorly represented, appear to live on various genera of Scrophulariaceae distributed mainly in the southern hemisphere (Caldara 2003; Caldara et al. 2010). In contrast, the Palaearctic species of *Miarus* and *Cleopomiarus* are associated with the genera of Campanulaceae in the subfamily Campanuloideae (*Campanula*, *Jasione*, *Phyteuma*), whereas the *Cleopomiarus* species in South Africa and in the southern part of North America live on the genera of the subfamilies Campanuloideae (*Roella*, *Wahlenbergia*) and Lobelioideae (*Lobelia*) (Caldara 2005, 2007; Caldara and Legalov 2016; Prena and O’Brien 2017). However, it is noteworthy that the systematics of Campanuloideae, especially of *Campanula* s.l. and close genera is still highly unstable (see APG 2016). This plant family is less phylogenetically close to Scrophulariaceae and Plantaginaceae and placed in Order Asterales (APG 2016). *Cleopomiarus* and *Miarus* are very closely related each other and morphologically somewhat far from the other mecinine genera, as recent taxonomic revisions have shown (Caldara 2001, 2005, 2007; Caldara and Legalov 2016; Jiang et al. 2018).

The general habitus of the imagoes of all *Cleopomiarus* and *Miarus* species is very uniform, and there are few external characters allowing differentiation of many species. Species recognition is often possible only by the careful examination of male or female genitalia. The presence of a deep prosternal canal and free claws are two easily observed external characters that immediately allow the separation of *Cleopomiarus* and *Miarus* from other Mecinini. The shape of the penis and the sclerites of the endophallus, the slightly more pronounced convexity of the male pygidium, and the more globose femora distinguish *Cleopomiarus* from *Miarus*. Moreover, in many species of *Cleopomiarus*, meso- and metafemora are dentate, and the uncus of the male metatibiae is enlarged, whereas the fifth ventrite of *Miarus* often shows a median fovea and two teeth placed posterolaterally. Finally, both genera feed on Campanulaceae, a family of plants apparently not parasitized by any other weevil. Preliminary molecular studies appear to confirm the systematic separation of these two genera, whereas several species of *Miarus*, well identified on the basis of morphological characteristics, tend to have very similar DNA fragments on mitochondrial COI gene (Vahtera and Muona 2006; Hendrich et al. 2015; Horecka et al. 2017; I Toševski, unpublished data). It is clear that more characters are required to separate these two genera from each other and from other Mecinini genera.

A detailed study of immatures might reveal more defining characters. To date, larvae of only 19 Mecinini species have been described (Gardner 1934; Emden 1938; Scherf 1964; Anderson 1973; Lee and Morimoto 1988; May 1993; Gosik 2010; Jiang and Zhang 2015), while descriptions of pupae are known for ten Mecinini species (Scherf 1964; Anderson 1973; Gosik 2010; Jiang and Zhang 2015). However, there are only four detailed descriptions of larvae and pupae that can be used for an adequate taxonomic comparison; these include immatures of three species of *Gymnetron* (Jiang and Zhang 2015) and one species of *Rhinusa* (Gosik 2010). In fact, the comparison with other previously described immatures, e.g., *Gymnetronanagallis* Marshall, 1933 (Gardner 1934; Emden 1938); *Mecinusheydenii* Wencker, 1866 (Emden 1938); *M.janthinus* Germar, 1821 (Scherf 1964); *Gymnetronbeccabungae* (Linnaeus, 1760), *G.villosulum* Gyllenhal, 1838; *Rhinusacollina* (Gyllenhal, 1813); *R.linariae* (Panzer, 1795); *Cleopomiarusgraminis*, *C.hispidulus* (LeConte, 1876), and *Miaruscampanulae* (Emden 1938; Scherf 1964; Anderson 1973) is somewhat problematic due to the missing details of the chaetotaxy and/or the absence of quality drawings.

Therefore, the purpose of this study was the following: 1) to describe larvae and pupae of *Miarus* and *Cleopomiarus* in detail for the first time, confirming when necessary the identity of the immatures by the study of the DNA COI barcode; 2) to find characters distinctive between these two genera and between the species; and 3) to investigate the relationships of these two genera with other genera of the same tribe and other tribes within Curculioninae.

## Materials and methods

### Insect collection

Immature specimens examined in this study came from material preserved at the British Museum of Natural History (London), the Department of Zoology University collection of Maria Curie-Skłodowska (Lublin) and from personal collections of the two authors (RC and IT) which are deposited in the collection of the Group Function of Invertebrate and Plant Biodiversity in Agro-Ecosystems of the Crop Research Institute (Prague, Czech Republic). In the last case, the specimens were collected and placed in tubes with 95% ethyl alcohol generally with a few adults. Since it is well known that more than one species of the complex *Miarus* + *Cleopomiarus* can be found on the same plant (Caldara 2007; Caldara and Legalov 2016), to be completely sure of the identification of some immatures, the DNA COI barcode of some specimens was also studied and compared with adults found in the same plant or with data already deposited in GenBank. The collectors identified the plants.

### Morphological descriptions

Part of the larval and pupal material was preserved in Pampel fixation liquid (see Trnka et al. 2015) and used for the morphological descriptions. To prepare the slides, we followed May (1994): a larva was decapitated, and the head was cleared in a 10% potassium hydroxide (KOH) solution and then rinsed in distilled water. After clearing, the mouthparts were separated from the head capsule, and the head capsule and all mouthparts were mounted on permanent microscope slides in Euparal. All other body parts were mounted on temporary microscope slides in 10% glycerine.

The observations and measurements were conducted using a light microscope with calibrated oculars (Olympus BX 40 and Nikon Eclipse 80i). The following characters were measured for each larva: head width, body length (larvae fixed in a C-shape were measured in segments), and body width in the widest place (i.e., metathorax or abdominal segments I–IV). For the pupae, the length and width at the widest place were measured. The lengths of all setae are visible on Figures.

Drawings were created with a drawing tube on a light microscope and processed by a computer (Adobe Photoshop, Corel Photo-Paint 11, GIMP 2). The numbers of setae of the bilateral structures are given for one side.

We used the terms and abbreviations for the setae of the mature larvae and pupae found in Scherf (1964), May (1977, 1994), and Marvaldi (1998, 1999).

### Molecular analysis

For molecular analysis, DNA was extracted from larvae and adults collected from seed capsules or flowers of plants belonging to the Campanulaceae. The barcoding region of the mitochondrial cytochrome c oxidase subunit I gene (mtCOI) was used to confirm the identity of the sampled larvae and the corresponding adults previously determined by using morphological characteristics (Caldara 2007; Caldara and Legalov 2016). Genomic DNA was extracted using the DNeasy Blood and Tissue Kit (Qiagen Inc., Valencia, CA) following the manufacturer’s instructions. The barcoding region of the mtCOI gene was amplified using the *de novo* designed primer pair for *Miarus* and *Cleopomiarus* species, MiaF (5’ CATGATCAGGAATACTMGGAACATC 3’) and MiaR (5’ GCTCGTGTATCAACATCTATTCC 3’). The MiaF/MiaR primers amplified a mtCOI product of 838 bp, which consisted of 635 bp of the barcoding region (Hebert et al. 2003).

Each PCR reaction was carried out in a volume of 20 μl [1 μl of DNA, 11.8 μl of H2O, 2 μl of High Yield Reaction Buffer A (1 × 1.5 mM MgCl2), 1.8 μl of MgCl2 (2.25 mM), 1.2 μl of dNTP (0.6 mM), 1 μl of each primer of the pair MiaF/MiaR (0.5 μM) and 0.2 μl of KAPATaq DNA polymerase (0.0375 U/μl) (Kapa Biosystems Inc. USA)]. The PCR protocol consisted of an initial denaturation at 95 °C for 5 min; 35 cycles consisting of three steps, i.e., 1 min at 94 °C, 1 min at 54 °C and 1.5 min at 72 °C; and a final extension step at 72 °C for 7 min. After PCR amplification, the products were separated on a 1% agarose gel, stained with ethidium bromide, and visualized under a UV transilluminator. The amplified products were sequenced by Macrogen Inc. (Seoul, Korea). The sequence data were deposited in the NCBI GenBank database (http://www.ncbi.nlm.nih.gov) under accession number MH558545–MH558548.

## Results

### Morphology of immature stages

#### *Description of the mature larva* (L3)

##### Genus Cleopomiarus

Taxon classificationAnimaliaColeopteraCurculionidae

Pierce, 1919

###### Description.

*Measurements* (in mm). Body length: 2.20–8.70. Body width (metathorax or abdominal segments I–II) 0.73–2.44. Head width: 0.35–1.16.

*General.* Body elongated, slender, rounded in cross section.

*Colouration.* From yellow to pale brown head. All thoracic and abdominal segments from distinctly white to slightly yellow.

*Vestiture.* Setae on body thin, in different colouration, distinctly different in length; piliform, often with some asperities.

*Head capsule*. Head oval or suboval, slightly or more flattened laterally, endocarinal line present and very distinct, more than half the length of frons. Frontal sutures on the head in different sizes, and ever extended to antennae. One or two stemmata (st), anterior stemma in the form of a pigmented spot with convex cornea behind the antenna. Dorsum of the epicranium with five setae; *des_3_* located anteriorly on epicranium close border with frontal suture. Frons with four setae; *fs_2_* absent; *fs_4_*; and *fs_5_* subequal. Head also with two *les* and two *ves.* Epicranial area with three *pes* and 2–3 sensilla.

*Antennae* located at the end of the frontal suture on each side, membranous and distinctly convex basal article bearing 3–4 sensilla and one conical sensorium, the later elongated, narrow.

*Clypeus* transverse-shaped, approximately 2.5–3 times as wide as long with two *cls*, and one sensillum (clss) between setae; all very close to margin with frons.

*Mouthparts.* Labrum with three piliform *lms*; anterior margin bisinuate. Epipharynx with three finger-like *als*; with 2–3 *ams*; and 0–1 *mes*; labral rods (lr) distinct, elongated. Mandibles distinctly broad, bifid, teeth of unequal height; slightly truncate; both setae piliform. Maxilla stipes with one *stps*, two *pfs* and one *mbs* and one sensillum; mala with six finger-like *dms*; five *vms*; all *vms* distinctly shorter than *dms.* Maxillary palpi with two palpomeres; basal palpomere with one short *mxps* and two sensilla; distal palpomere with one sensillum and a group of micro cuticular apical processes. Prelabium oval-shaped, with one *prms*; ligula with sinuate margin and 1–2 *ligs*; premental sclerite well sclerotized but without anterior and posterior extensions, U-shaped. Labial palpi with two palpomeres (partially appears as one palpomere); each of the palpomeres with one sensillum, distal palpomere with cuticular apical processes. Postlabium with three *pms*, all located laterally.

*Thorax.* Prothorax slightly smaller than meso- and metathorax. Spiracle bicameral, placed between the pro- and mesothorax (see, e.g., Skuhrovec et al. 2015). Prothorax with 9–10 *prns*; two *ps*; and one *eus.* Mesothorax with one *prs*, three *pds*; one *as*; two long and one short *ss*; one *eps*; one *ps*; and one *eus.* Chaetotaxy of metathorax almost identical to that of mesothorax. Each pedal area of thoracic segments well separated, with 5–6 *pda*.

*Abdomen.* Abdominal segments I–III of almost equal length, next abdominal segments decreasing gradually to the terminal parts of the body. Abdominal segment X reduced to four anal lobes of unequal size, the lateral lobes being distinctly the largest, the dorsal and the ventral lobes being very small. Anus located terminally. Eight spiracles, bicameral, all spiracles functional, close to the anterior margin. Abdominal segments I–VII with one *prs*; three *pds*, *pds_2_* the longest one; one long and one minute *ss*; two long *eps*; one *ps*; one *lsts*; and two *eus.* Abdominal segment VIII with one *prs*; 2–3 *pds*, if there are three setae, then *pds_2_* the longest one; one long and one minute *ss*; two long *eps*; one *ps*; one *lsts*; and two *eus.* Abdominal segment IX with four *ds*; 1–2 *ps*; and 1–2 *sts.* Abdominal segment X with one minute seta present or absent.

##### Cleopomiarus distinctus

Taxon classificationAnimaliaColeopteraCurculionidae

(Boheman, 1845)

Figures 1 , 2 , 3–4 , 5–6 , 7 , 8–10

###### Material examined.

17 L3 larvae: 7 exx., 29.07.2010, Gródek ad Hrubieszów, CE Poland, leg. E. Szwaj, det. J. Łętowski; 10 exx., ex seed capsules of *Campanulacervicaria* L., 05.07.2017, Stara Planina, Babin Zub, east Serbia, leg. I. Toševski, all collected in association with adults det. R. Caldara. Accession numbers of sequenced specimens: MH558546.

###### Description.

*Measurements* (in mm). Body length: 4.43–5.57 (mean 4.90). Body width (metathorax or abdominal segments I–II) up to 1.37. Head width: 0.70–0.84 (mean 0.71).

**Figure 1.**
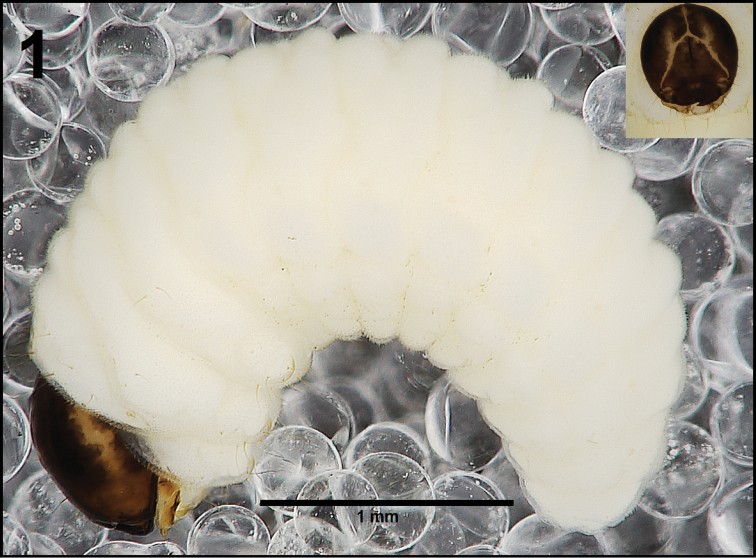
*Cleopomiarusdistinctus* mature larva habitus.

*General.* Body elongated, slender, curved, rounded in cross section (Fig. 1).

*Colouration.* Black head (Fig. 1). All thoracic and abdominal segments from distinctly white to slightly yellow (Fig. 1).

*Vestiture.* Setae on body thin, light yellow to greyish, distinctly different in length (minute to very long).

**Figure 2.**
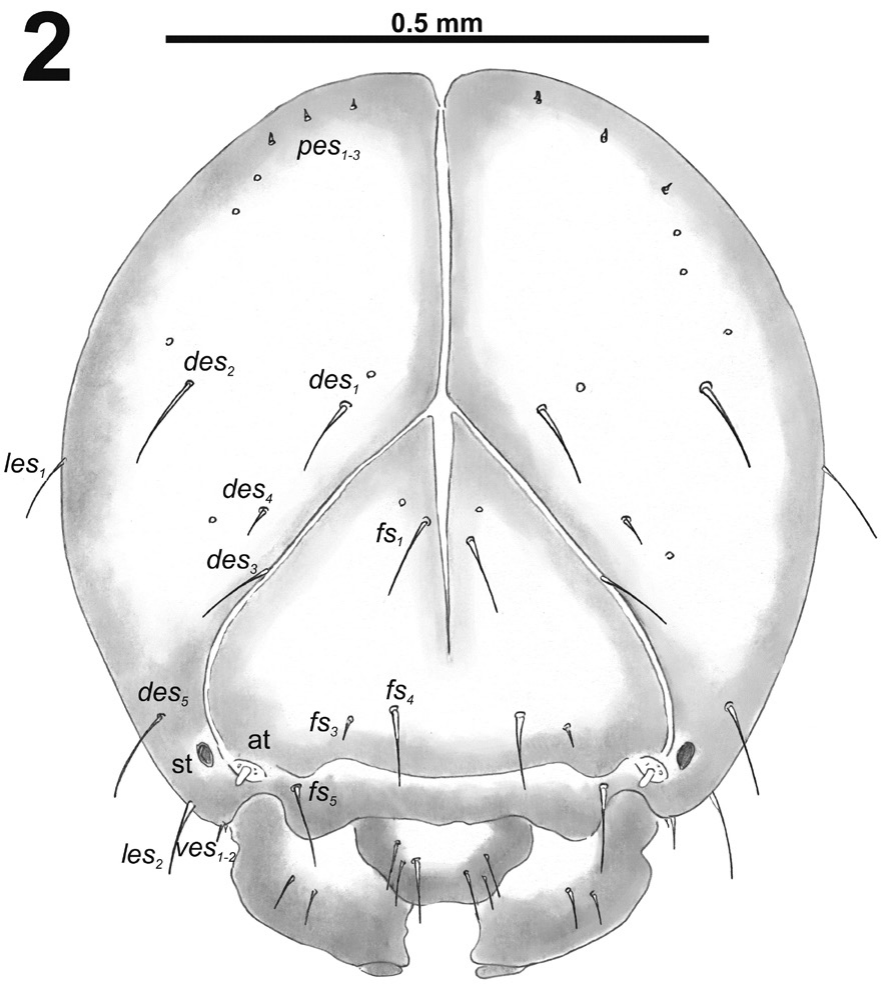
*Cleopomiarusdistinctus* mature larva head, frontal view. Abbreviations: *des* – dorsal epicranial s., *fs* – frontal epicranial s., *les* – lateral epicranial s., *pes* – postepicranial s., *ves* – ventral s., at – antenna, st – stemmata.

*Head capsule* (Fig. 2). Head oval, slightly flattened laterally. Frontal sutures distinct, seem as pallid stripes. Anterior stemma (st), in the form of a small pigmented spot. *Des_1–3_* and *des_5_* long; *des_4_* short (Fig. 2). *Fs_1_* long; *fs_2_* absent; *fs_3_* very short; *fs_4_* long; and long *fs_5_* (Fig. 2). *Les_1_* and *les_2_* as long as *des_5_*; both *ves* medium to very short. Epicranial area with three *pes* and two sensilla in line with *des_2_*.

**Figures 3–4.**
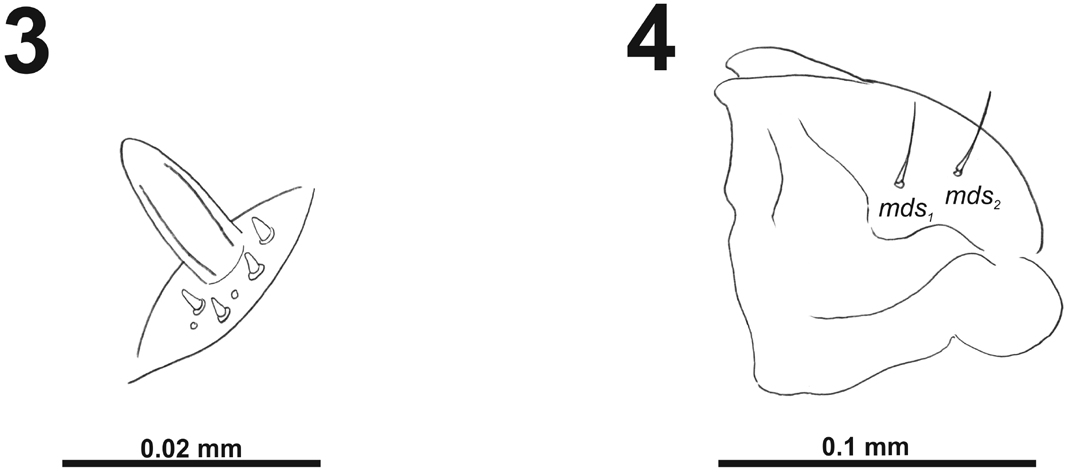
*Cleopomiarusdistinctus* mature larva. **3** Antenna **4** Right mandible. Abbreviation: *mds* – mandible dorsal s.

*Antennae* bearing one relatively elongated conical sensorium; and basal membranous article with four sensilla equal in length, and two pores (Fig. 3).

**Figures 5–6.**
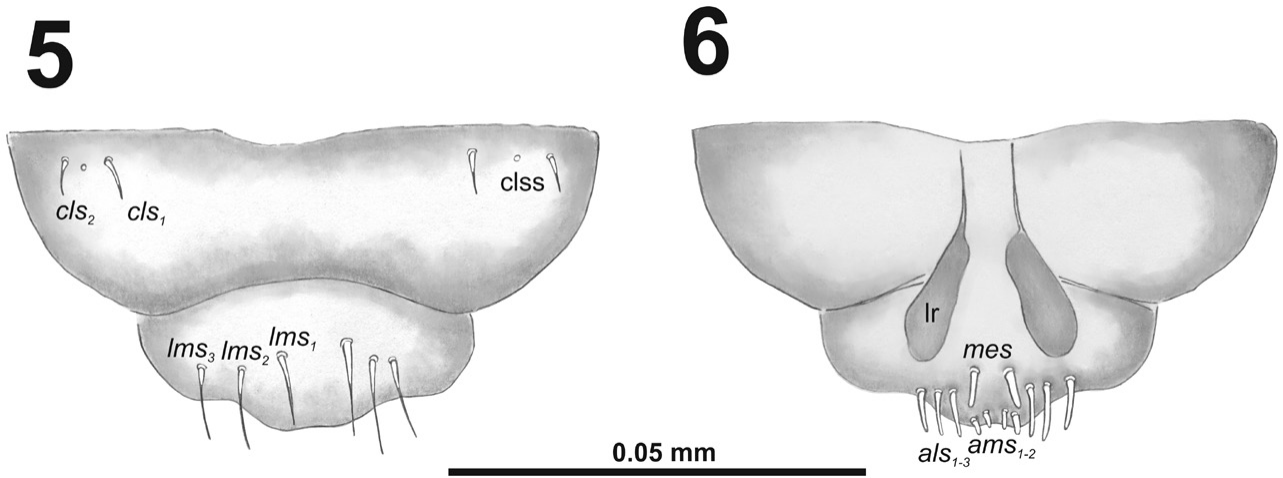
*Cleopomiarusdistinctus* mature larva, mouthparts. **5** Labrum and clypeus **6** Epipharynx. Abbreviations: *als* – anteriolateral s., *ams* – anteromedial s., *cls* – clypeal s., *lms* – labral s., *mes* – median s., clss – clypeal sensillum, lr – labral rods.

*Clypeus* (Fig. 5) approximately three times as wide as long with two *cls* of medium size, equal in length, and one sensillum; anterior margin sinuate.

**Figure 7.**
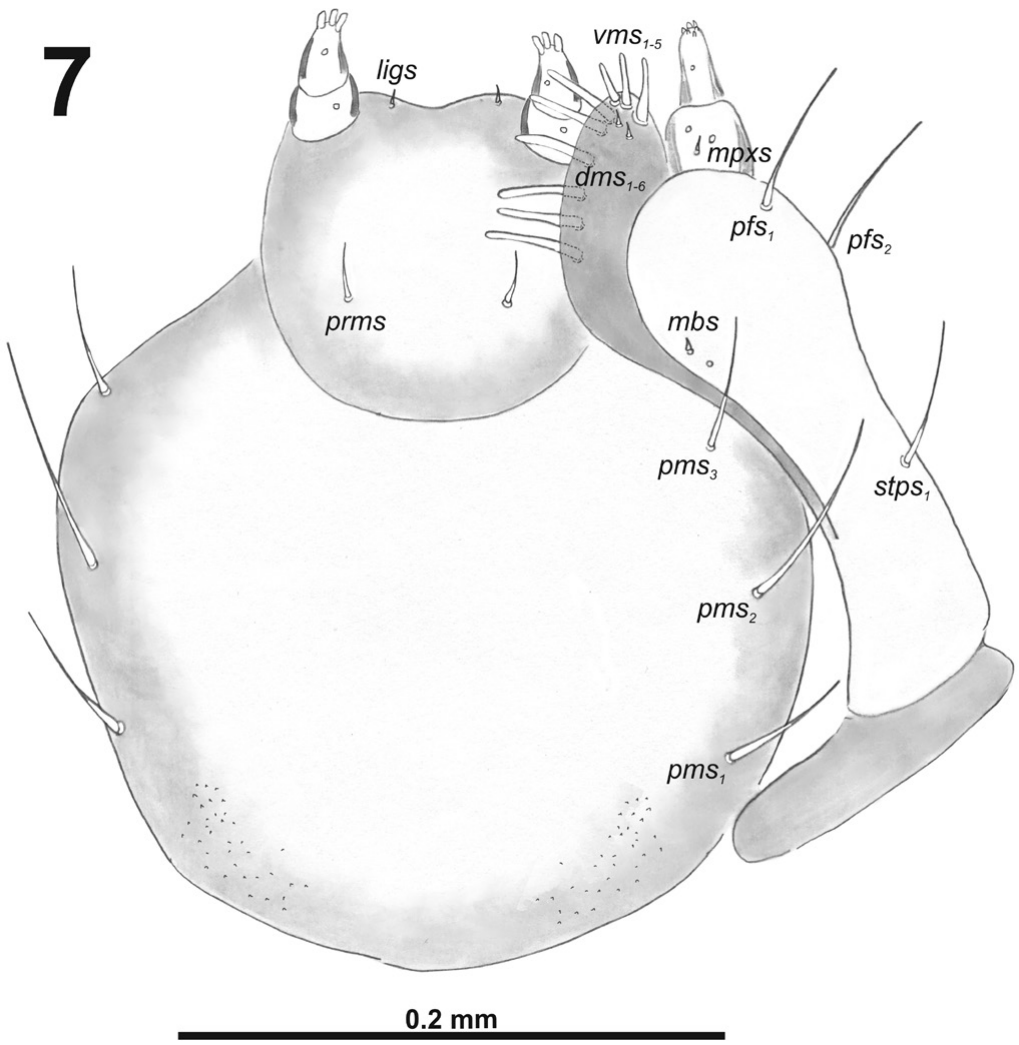
*Cleopomiarusdistinctus* larval mouthparts, maxillolabial complex, ventral view right maxilla. Abbreviations: *dms* – dorsal malar s., *vms* – ventral malar s., *mpxs* – maxillary palps s., *mbs* – basioventral s., *pfs* – palpiferal s., *stps* – stipital s., *prms* – premental s., *pms* – postmental s., *ligs* – ligular s.

*Mouthparts.* Labrum (Fig. 5) almost two times as wide as long, with three piliform *lms*, almost equal in the length; all located more or less anteromedially, *lms_2_* and *lms_3_*distinctly reach labral margin. Epipharynx (Fig. 6) with three medium sized finger-like *als*, all similar in length; with two rather short, equal in length *ams*; and one medium size, finger-like *mes*; labral rods (lr) distinct, elongated, slightly convex. Mandibles (Fig. 4) bifid; cutting edge with a blunt tooth; bearing with two setae in medium size, piliform, and aligned longitudinally. Maxilla (Fig. 7) stipes with long *stps* and both *pfs*, minute *mbs*, and one sensillum close to *mbs*; mala with six medium sized finger-like *dms*; five *vms*, three medium size, two very short. Maxillary palpi: basal palpomere with one short *mxps* and two sensilla; distal palpomeres with medium, cuticular apical processes; length ratio of basal and distal palpomeres 1:1. Prelabium (Fig. 7) with one short *prms*; ligula with one minute *ligs*; premental sclerite narrow, ring-shaped. Labial palpi with two palpomeres; length ratio of basal and distal palpomeres 1:1.2; each of the palpomeres with one sensillum, distal palpomeres with medium, cuticular apical processes. Postlabium (Fig. 7) with long *pms_1_* located basally, very long *pms_2_* located medially and long *pms_3_* located apically; membranous area basolaterally sparsely and finely asperate.

**Figures 8–10.**
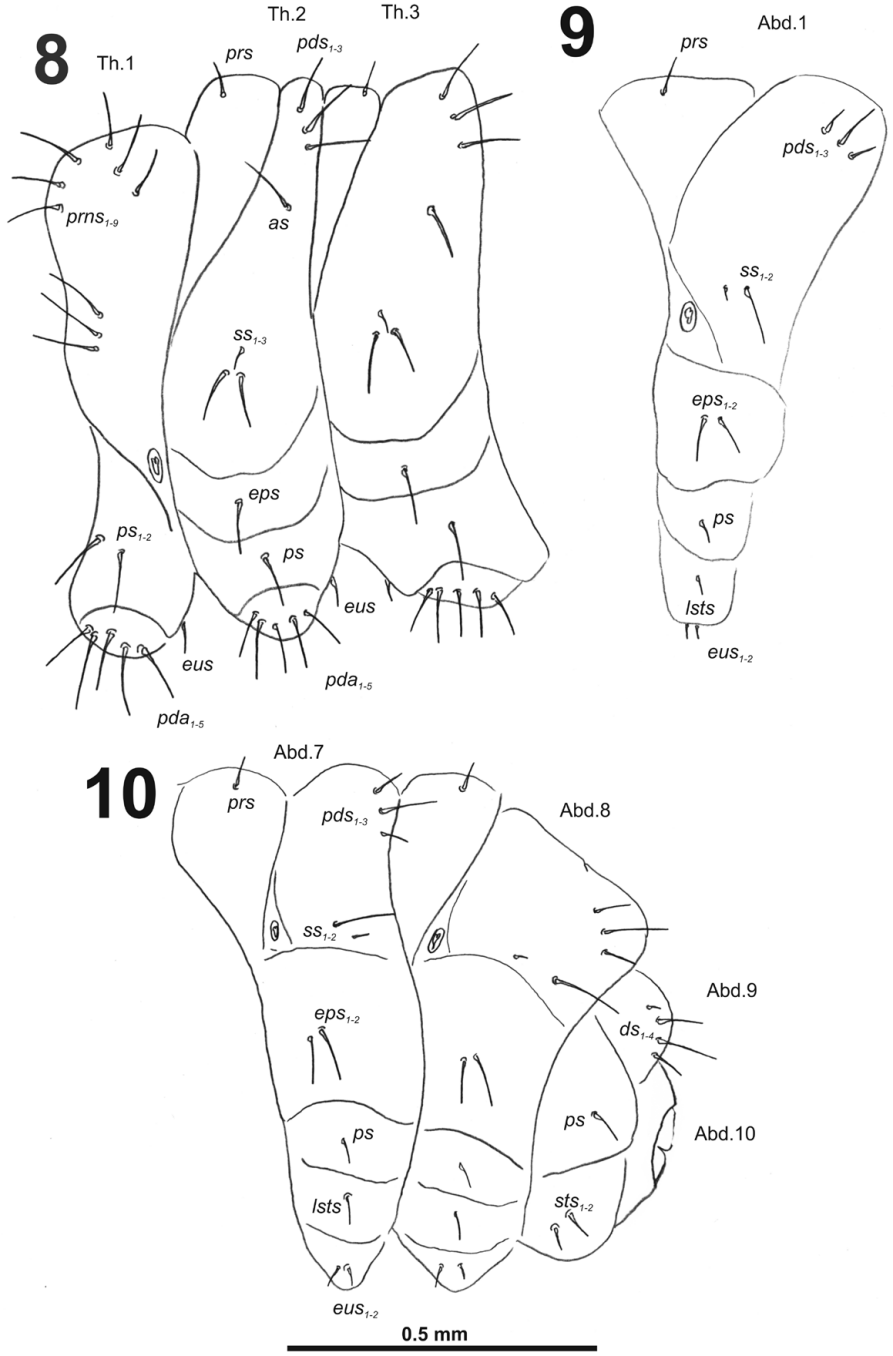
*Cleopomiarusdistinctus* mature larva, habitus. **8** Lateral view of thoracic segments **9** Lateral view of abdominal segment I **10** Lateral view of abdominal segments VII–X. Abbreviations: *as* – alar s., *ds* – dorsal s., *eps* – epipleural s., *eus* – eusternal s., *lsts* – laterosternal s., *pda* – pedal s., *pds* – postdorsal s., *prns* – pronotal s., *prs* – prodorsal s., *ss* – spiracular s., *ps* – pleural s., *sts* – sternal s., Th1–3 – number of thoracic segments, Ab1–10 – number of abdominal seg.

*Thorax.* Prothorax (Fig. 8) with nine very long *prns*, weakly pigmented dorsal sclerite present with six long *prns*, this sclerite subdivided in two triangular plates medially; two long *ps*; and one short *eus.* Meso- and metathorax (Fig. 8) with one medium *prs*, three long *pds*; one long *as*; two very long and one minute *ss*; one long *eps*; one long *ps*; and one very short *eus.* Each pedal area of the thoracic segments with five very long *pda*.

*Abdomen.* Abdominal segments I–VII (Figs 9–10) with one medium *prs*; one long and two medium size *pds* (order: medium, long, medium); one very long and one minute *ss*; two very long *eps*; one medium *ps*; one medium *lsts*; and two very short *eus.*Abdominal segment VIII (Fig. 10) with one very short to minute *prs*; one short and two long to relatively long *pds* (order: short, long, relatively long); one long and one minute *ss*; two very long *eps*; one medium *ps*; one medium *lsts*; and two very short *eus.* Abdominal segment IX (Fig. 10) with four short *ds*; one medium *ps*; and two short *sts.* Abdominal segment X (Fig. 10) without seta.

###### Biology.

This species lives on various species of *Campanula* (*C.glomerata* L., *C.incurva* Auch., *C.latifolia* L., *C.persicifolia* L., *C.rapunculus* L., *C.rhomboidalis* L., *C.thyrsoides* L., *C.trachelium* L.) in central Europe (Hoffmann 1958; Smreczyński 1976; Caldara and Legalov 2016). It was never reported to feed on *C.cervicaria* L., a species widely distributed in Europe. Larvae are seed feeders developing inside seed capsules.

###### Remarks.

This is one of the most variable species and with the widest Palaearctic distribution in the genus (Europe and central and northern Asia to the Russian Far East) (Caldara and Legalov 2016; Jiang et al. 2018). The three most variable characters in adults are the colour of the dorsal vestiture, which varies from whitish grey to light brown, the density of the elytral scales, which sometimes completely cover the integument, and the length of the rostrum, especially in the female and Anatolian populations. It is clear that it would be very interesting to perform a detailed molecular study of these populations. Apart from the characters of the shape of the rostra, the uncus of the male metatibiae and that of the penis, this species differs from *C.graminis* and related species also by the more angulate shape of the elytral base. Also the immatures of *C.distinctus* can easily be separated from those of *C.graminis* by several characters in larvae: postlabium with medium size *pms_1_* and *pms_3_*, a very long *pms_2_* (Fig. 7) and a membranous area of postlabium basolaterally finely asperate as well as in pupae: *Vs* and *sos* absent (or as microsetae) (Fig. 82), pronotum with one *sls* (Fig. 83), and abdominal segments I–VII without ventral setae (Fig. 82). Finally, we could confirm that these two species are well separated molecularly as previously reported (Vahtera and Muona 2006; Hendrich et al. 2015; Horecka et al. 2017).

##### Cleopomiarus graminis

Taxon classificationAnimaliaColeopteraCurculionidae

(Gyllenhal, 1813)

Figures 11 , 12 , 13–14 , 15–16 , 17 , 18–20

###### Material examined.

11 L3 larvae: 6 exx., 18.07.2010, Wólka ad Lublin, CE Poland, leg. E. Szwaj, det. J. Łętowski; 5 exx., ex seed capsules of *Campanulamacrostachya* Waldst. et Kit. ex Willd., Dobra, Iron Gate, east Serbia. 13.07.2015, leg. I. Toševski, all collected in association with adults, det. R. Caldara. Accession numbers of sequenced specimens: MH558545.

###### Description.

*Measurements* (in mm). Body length: 3.75–6.27 (mean 4.80). Body width (metathorax or abdominal segments I–II) up to 1.63. Head width: 0.65–0.78 (mean 0.71).

**Figure 11.**
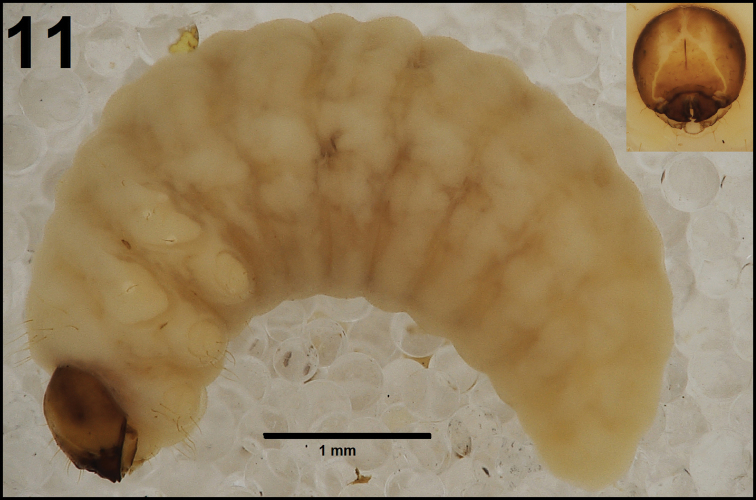
*Cleopomiarusgraminis* mature larva habitus.

*General.* Body elongated, slender, curved, rounded in cross section (Fig. 11).

*Colouration.* Pale brown head (Fig. 11). All thoracic and abdominal segments from distinctly white to slightly yellow (Fig. 11).

*Vestiture.* Setae on body thin, slightly from orange to pale brown, distinctly different in length (minute to very short or long to very long). Cuticle distinctly asperate.

**Figure 12.**
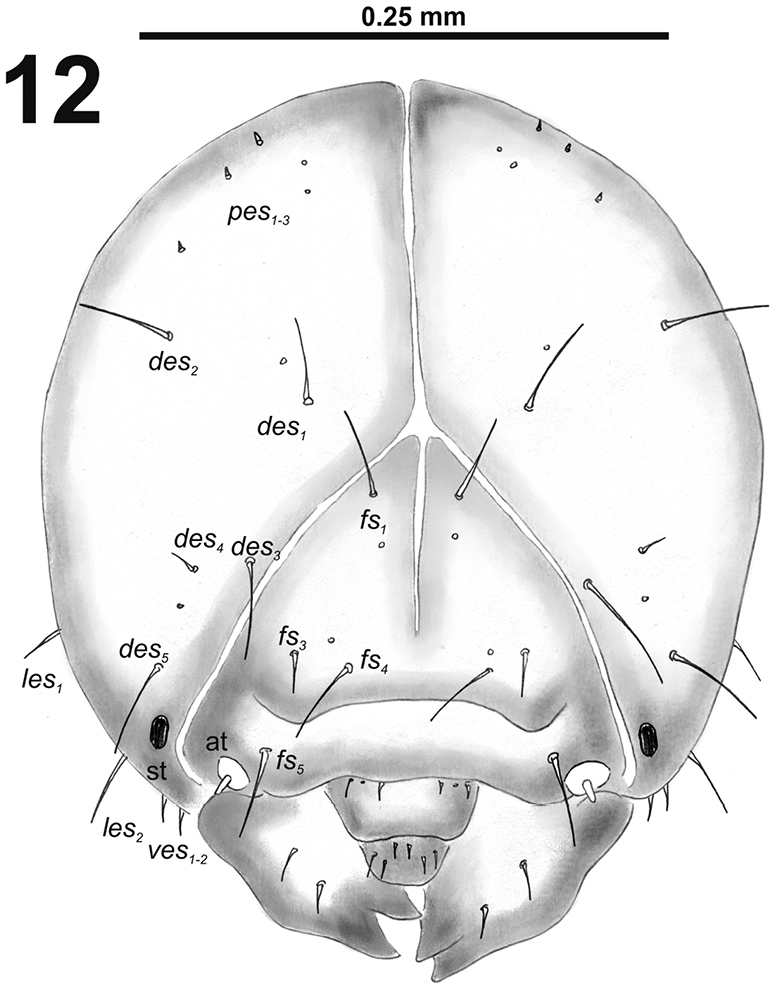
*Cleopomiarusgraminis* mature larva head, frontal view. Abbreviations: *des* – dorsal epicranial s., *fs* – frontal epicranial s., *les* – lateral epicranial s., *pes* – postepicranial s., *ves* – ventral s., at – antenna, st – stemmata.

*Head capsule* (Fig. 12). Head oval, slightly flattened laterally. Frontal sutures narrow, but distinct. Anterior stemma (st), in the form of a large pigmented spot. *Des_1–3_* and *des_5_* long; *des_4_* short to very short (Fig. 12). *Fs_1_* long; *fs_2_* absent; *fs_3_* very short; *fs_4_* long; and long *fs_5_* (Fig. 12). *Les_1_* and *les_2_* as long as *des_5_*; both *ves* very short. Epicranial area with two sensilla and three minute *pes* in line with *des_2_*.

**Figures 13–14.**
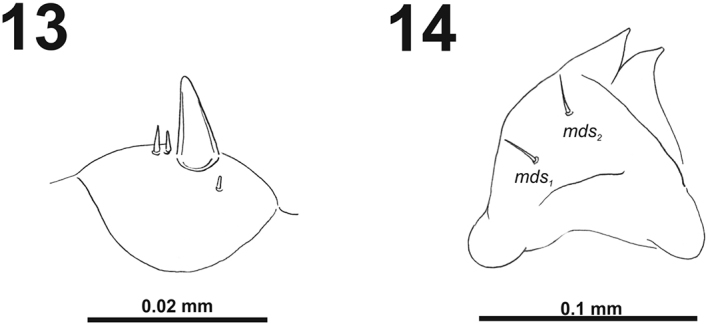
*Cleopomiarusgraminis* mature larva. **13** Antenna **14** Right mandible. Abbreviation: *mds* – mandible dorsal s.

*Antennae* bearing one medium size conical sensorium, and basal membranous article with three sensilla different in length, two behind conical sensorium, and one ahead of it (Fig. 13).

**Figures 15–16.**
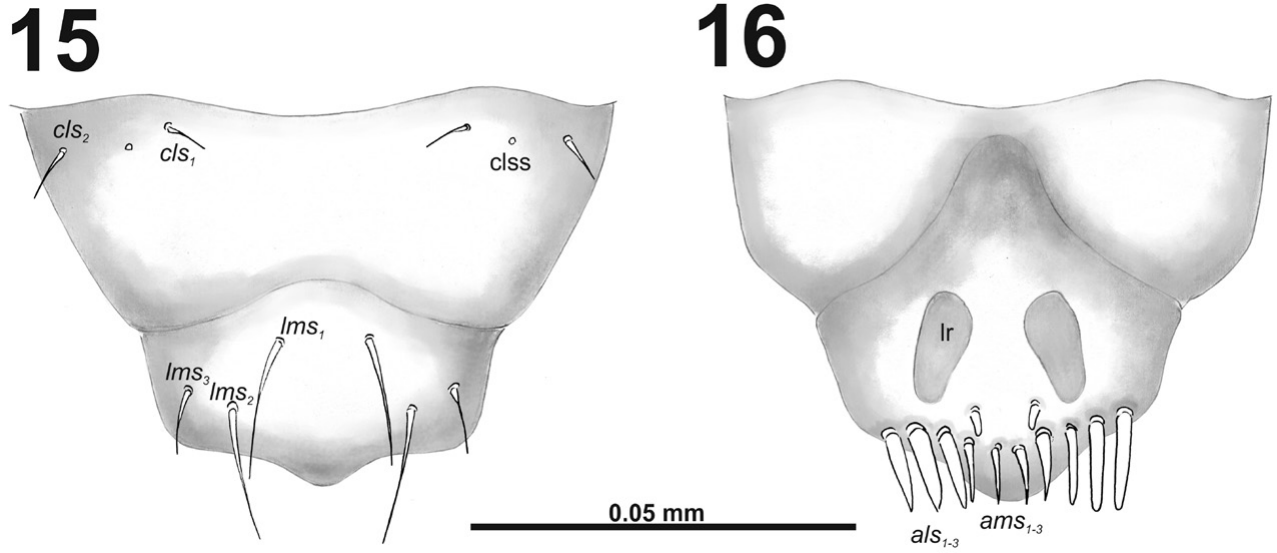
*Cleopomiarusgraminis* mature larva, mouthparts. **15** Labrum and clypeus **16** Epipharynx. Abbreviations: *als* – anteriolateral s., *ams* – anteromedial s., *cls* – clypeal s., *lms* – labral s., *mes* – median s., clss – clypeal sensillum, lr – labral rods.

*Clypeus* (Fig. 15) approximately 2.5–3 times as wide as long with two short *cls*, *cls_1_* slightly shorter than *cls_2_*, and one sensillum; anterior margin sinuate.

**Figure 17.**
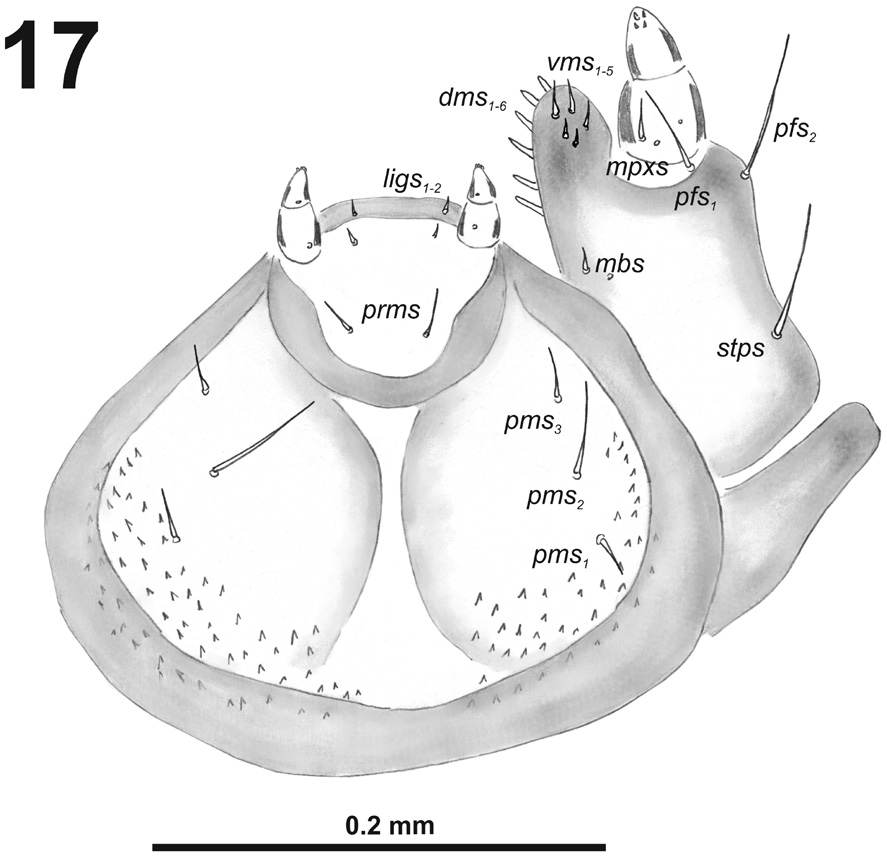
*Cleopomiarusgraminis* larval mouthparts, maxillolabial complex, ventral view right maxilla. Abbreviations: *dms* – dorsal malar s., *vms* – ventral malar s., *mpxs* – maxillary palps s., *mbs* – basioventral s., *pfs* – palpiferal s., *stps* – stipital s., *prms* – premental s., *pms* – postmental s., *ligs* – ligular s.

*Mouthparts.* Labrum (Fig. 15) less than two times as wide as long, with three piliform *lms*, different in the length; *lms_1_* located anteromedially, very close to margin of clypeus, *lms_2_* located in the middle, and *lms_3_* located anterolaterally; *lms_1_* and *lms_2_* of medium size, and *lms_3_* distinctly shorter than the previous two; only *lms_2_* distinctly reaches labral margin. Epipharynx (Fig. 16) with three long finger-like *als*, all of identical in length; with three *ams* in different length, *ams_1_* and *ams_2_* piliform of medium size, finger-like short *ams_3_* and enlarged in middle, and also located more close to lr; without *mes*; labral rods (lr) distinct, elongated, oval. Mandibles (Fig. 14) bifid; bearing with two setae in medium size, piliform, and aligned longitudinally, *mds_1_* located basally; *mds_2_*, located distinctly apically. Maxilla (Fig. 17) stipes with very long *stps* and *pfs_2_*, medium *pfs_1_*, very short to minute *mbs*, and sensillum close to *mbs*; mala with six medium sized finger-like *dms*; five *vms*, different in length, three setae medium size, and two setae very short. Maxillary palpi: basal palpomere with one short *mxps* and two sensilla; distal palpomere with some cuticular apical processes; length ratio of basal and distal palpomeres 1:0.8. Prelabium (Fig. 17) with one short *prms*; ligula with two very short to minute *ligs*; premental sclerite broad, ring-shaped. Labial palpi with two palpomeres; length ratio of basal and distal palpomeres 1:0.8; each of the palpomeres with one sensillum, distal palpomere with cuticular apical processes. Postlabium (Fig. 17) with short *pms_1_* located basally, very long *pms_2_* located medially and short *pms_3_* located apically; membranous area basolaterally distinctly asperate.

**Figures 18–20.**
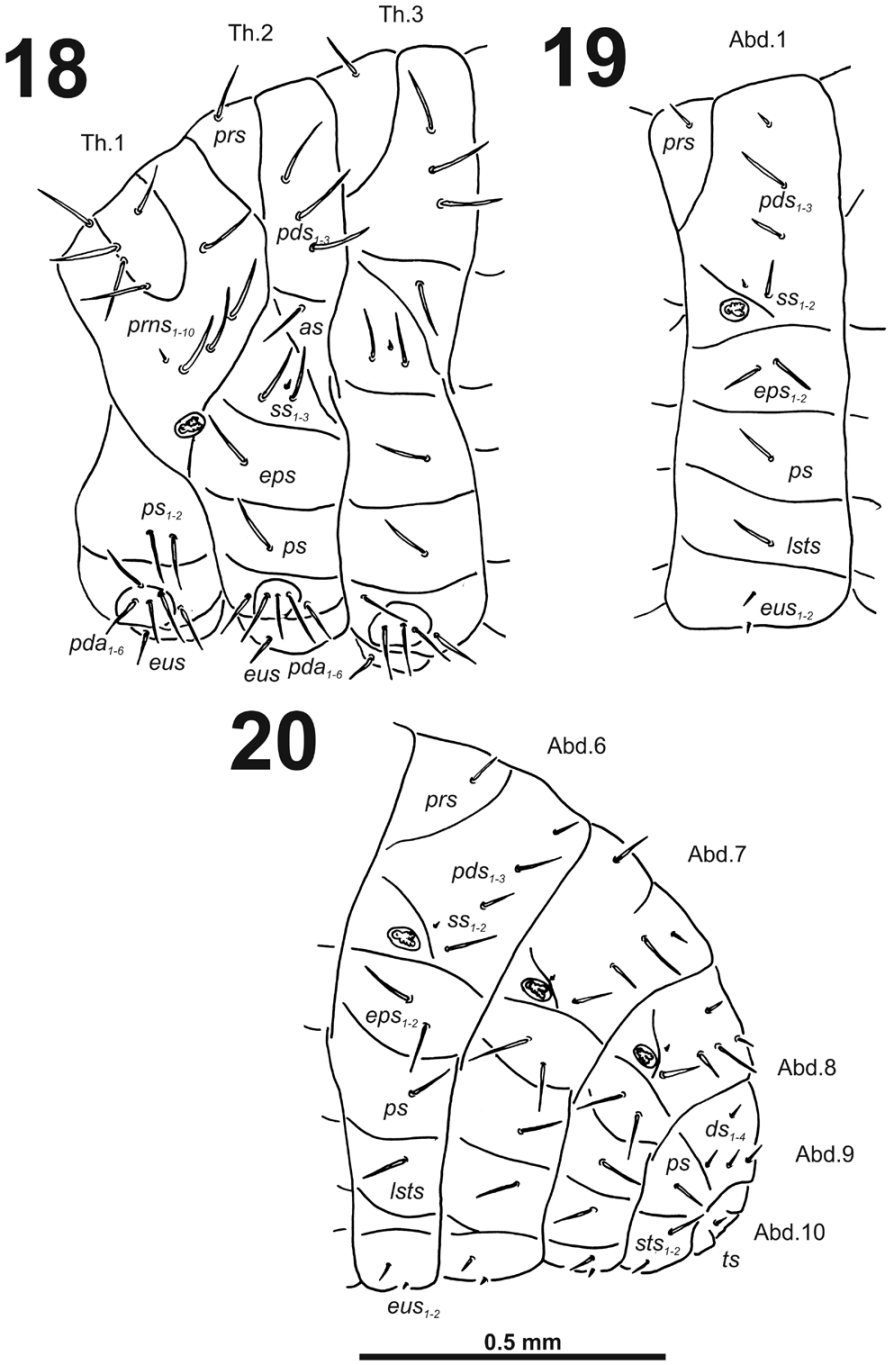
*Cleopomiarusgraminis* mature larva, habitus. **18** Lateral view of thoracic segments **19** Lateral view of abdominal segment I **20** Lateral view of abdominal segments VI–X. Abbreviations: *as* – alar s., *ds* – dorsal s., *eps* – epipleural s., *eus* – eusternal s., *lsts* – laterosternal s., *pda* – pedal s., *pds* – postdorsal s., *prns* – pronotal s., *prs* – prodorsal s., *ss* – spiracular s., *ps* – pleural s., *sts* – sternal s., *ts* – terminal s., Th1–3 – number of thoracic segments, Ab1–10 – number of abdominal seg.

*Thorax.* Prothorax (Fig. 18) with nine very long and one very short to minute *prns*, small pigmented dorsal sclerite present with five long *prns*, this sclerite subdivided in two triangular plates medially; two very long to long *ps*; and one short *eus.* Meso- and metathorax (Fig. 18) with one long *prs*, three very long *pds*; one very long *as*; two very long and one very short to minute *ss*; one very long *eps*; one very long *ps*; and one short to very short *eus.* Each pedal area of the thoracic segments with 5–6 very long *pda*.

*Abdomen.* Abdominal segments I–VII (Figs 19–20) with one medium *prs*; one short and two very long to long *pds* (order: short, very long, long); one very long and one minute *ss*; two long *eps*; one very long *ps*; one long *lsts*; and two short to very short *eus.* Abdominal segment VIII (Fig. 20) with one very short to minute *prs*; one short and two long to relatively long *pds* (order: short, long, relatively long); one long and one minute *ss*; two very long *eps*; one long *ps*; one long *lsts*; and two short to very short *eus.* Abdominal segment IX (Fig. 20) with three relatively long and one short to very short *ds*; one relatively long and sometimes one minute *ps*; and one relatively long to short and one short to very short *sts.* Abdominal segment X (Fig. 20) with one very short seta (*ts*).

###### Biology.

Larvae were collected while feeding on the seeds of several species of *Campanula*, mainly *C.glomerata*, *C.persicifolia*, and *C.rotundifolia* L. (Hustache 1932; Hoffmann 1958; Smreczyński 1976; Lohse and Tischler 1983; Caldara and Legalov 2016) without producing galls. The species was not previously reported on *Campanulamacrostachya* Waldst. and Kit. ex Willd., a taxon distributed from Ukraine along the Balkans until Anatolia. Pupae, as well as immatures of *M.ajugae*, were also collected on *Adenophoraliliifolia* (L.) A. DC, although in another Serbian locality (see below). This genus, however, is very closely related to *Campanula* (Cano-Maqueda and Talavera 2011).

###### Remarks.

This is a very common and variable species with a wide European and Asian distribution from the Iberian Peninsula to eastern China (Caldara and Legalov 2016; Jiang et al. 2018). The two most variable characters in adults are the colour of the dorsal vestiture, which varies from whitish grey to light brown, and the density of the elytral scales, sometimes completely covering the integument. The rostrum varies somewhat in length and curvature, especially in the female. *Cleopomiarusgraminis* is very closely related to *C.longirostris* as demonstrated by our data on the molecular fragment COI (I Toševski, unpublished data). Therefore, the differences between these two taxa found in the study of the immature stages, especially in the larvae – antennae with a very long conical sensorium and three sensilla (Figs 13, 33), dorsal setae (except *des_4_*) long (Figs 12, 32), prothorax with nine very long and one very short to minute *prns* (Figs 18, 38) – are very important in order to confirm the specific rank of both taxa. On the other hand, the larva of *C.longirostris* is distinctly longer than the larva of *C.graminis*. With regard to the differences from *C.distinctus*, another widespread sympatric species sometimes confused with *C.graminis*, see the Remarks for the former taxon.

##### Cleopomiarus longirostris

Taxon classificationAnimaliaColeopteraCurculionidae

(Gyllenhal, 1838)

Figures 21 , 22 , 23–24 , 25–26 , 27 , 28–30

###### Material examined.

11 L3 larvae: south-eastern France, Menton, July 2007, ex capsules of *Campanulatrachelium* L., leg. and det. R. Caldara, all determined by association with reared adults.

###### Description.

*Measurements* (in mm). Body length: 6.60–8.70 (mean 8.3). Body width (abdominal segments I–III) up to 2.44. Head width: 1.05–1.16 (mean 1.10).

**Figure 21.**
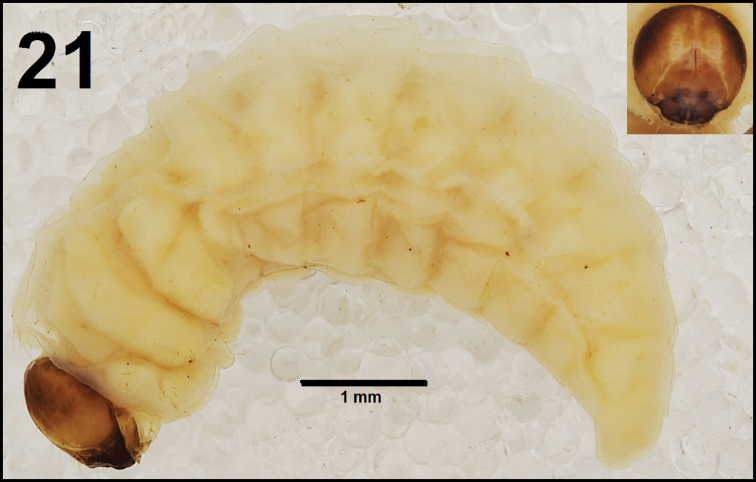
*Cleopomiaruslongirostris* mature larva habitus.

*General.* Body elongated, slender, curved, rounded in cross section (Fig. 21).

*Colouration.* Pale brown head with indistinct pattern around frontal sutures (Fig. 21). All thoracic and abdominal segments from distinctly white to slightly yellow (Fig. 21).

*Vestiture.* Setae on body thin, orange, distinctly different in length (minute to very short or long to very long). Cuticle slightly asperate.

**Figure 22.**
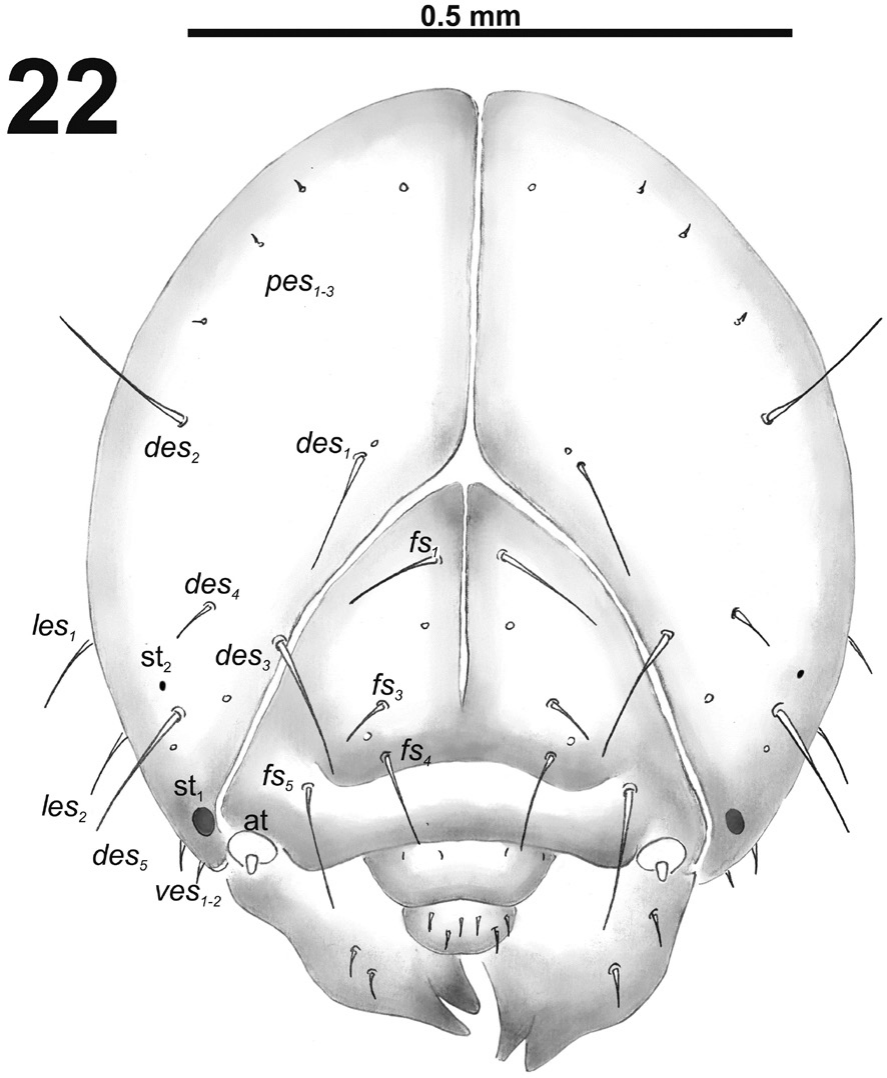
*Cleopomiaruslongirostris* mature larva head, frontal view. Abbreviations: *des* – dorsal epicranial s., *fs* – frontal epicranial s., *les* – lateral epicranial s., *pes* – postepicranial s., *ves* – ventral s., at – antenna, st – stemmata.

*Head capsule* (Fig. 22). Head oval, slightly flattened laterally. Frontal sutures medium width, distinct. Two pairs of stemmata (st), anterior one in the form of a large pigmented spot; and posterior one in form of a very small pigmented spot, located on each side close *des_5_*. *Des_1–3_* long; *des_4_* short and *des_5_* long to very long (Fig. 22). *Fs_1_* long; *fs_2_* absent; *fs_3_* short; *fs_4_* long; and long *fs_5_* (Fig. 22). *Les_1_* and *les_2_* as long as *des_5_*; both *ves* very short. Epicranial area with three *pes* and two sensilla in line with *des_2_*.

**Figures 23–24.**
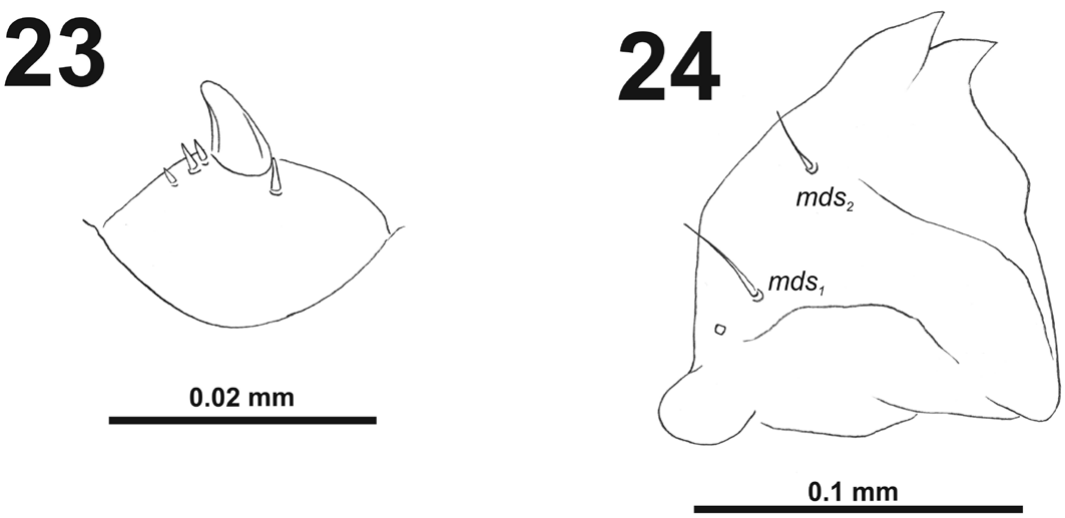
*Cleopomiaruslongirostris* mature larva. **23** Antenna **24** Right mandible. Abbreviation: *mds* – mandible dorsal s.

*Antennae* bearing one medium size conical sensorium, and basal membranous article with four sensilla different in length, three behind conical sensorium, and one ahead of it (Fig. 23).

**Figures 25–26.**
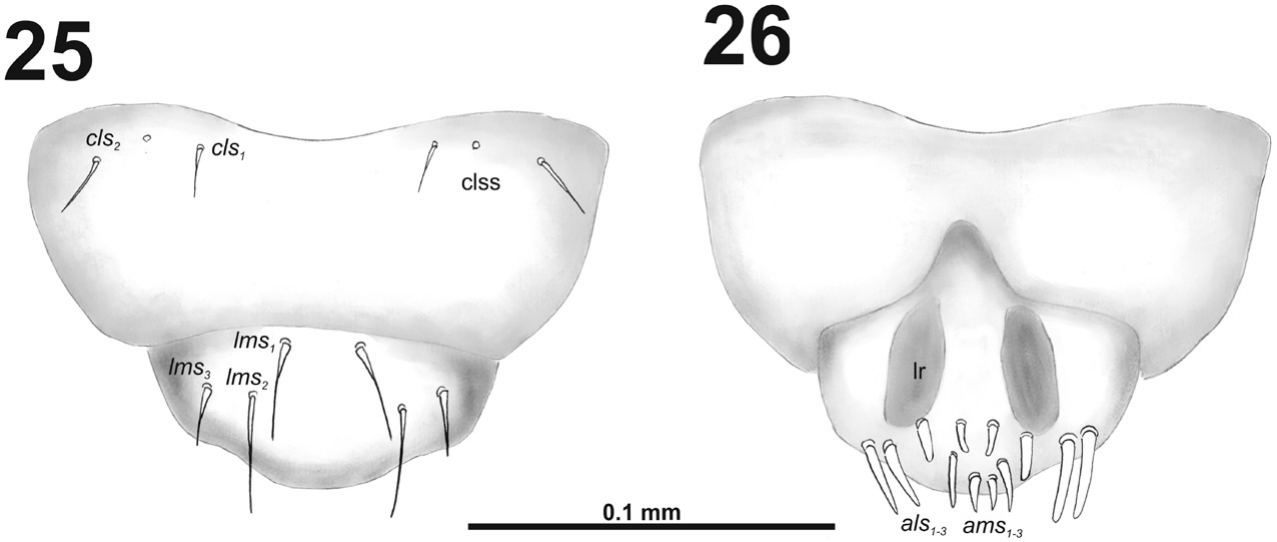
*Cleopomiaruslongirostris* mature larva, mouthparts. **25** Labrum and clypeus **26** Epipharynx. Abbreviations: *als* – anteriolateral s., *ams* – anteromedial s., *cls* – clypeal s., *lms* – labral s., *mes* – median s., clss – clypeal sensillium, lr – labral rods.

*Clypeus* (Fig. 25) approximately 2.5 times as wide as long with two short *cls*, *cls_2_* distinctly longer than *cls_1_*, and one sensillum.

**Figure 27.**
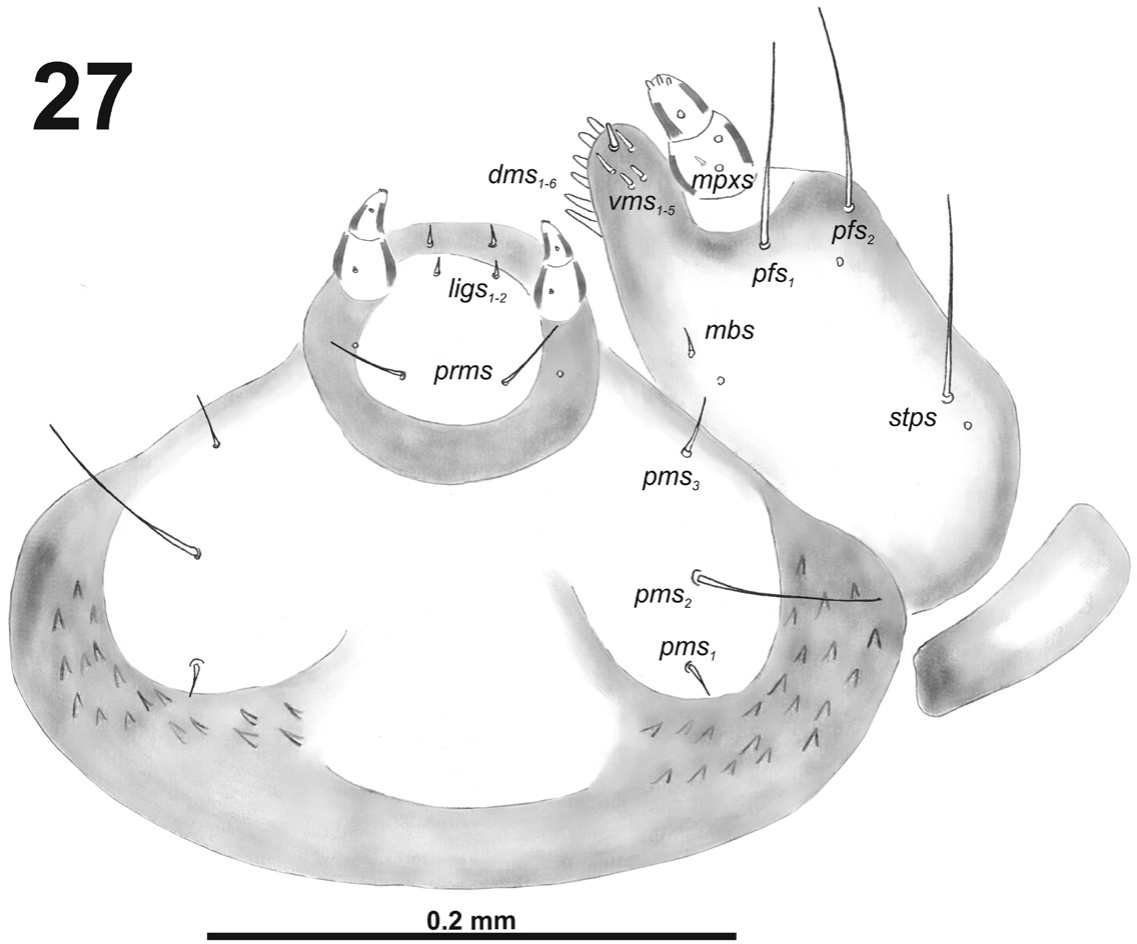
*Cleopomiaruslongirostris* larval mouthparts, maxillolabial complex, ventral view right maxilla. Abbreviations: *dms* – dorsal malar s., *vms* – ventral malar s., *mpxs* – maxillary palps s., *mbs* – basioventral s., *pfs* – palpiferal s., *stps* – stipital s., *prms* – premental s., *pms* – postmental s., *ligs* – ligular s.

*Mouthparts.* Labrum (Fig. 25) less than 2.5 times as wide as long, with three piliform *lms*, different in the length; *lms_1_* located anteromedially, close to margin, *lms_2_* located in the middle, and *lms_3_* located posterolaterally; *lms_1_* and *lms_2_* of medium size, and *lms_3_* distinctly shorter than the previous two; only *lms_2_* distinctly reaches labral margin. Epipharynx (Fig. 26) with three long finger-like *als*, two *als* of identical in length, and the third one distinctly shorter and also located more close to labral rods (lr); with three *ams* in different length, *ams_1_* and *ams_2_* piliform and short, finger-like *ams_3_* and enlarged in middle, and also located more close to lr; without *mes*; labral rods (lr) distinct, elongated, oval. Mandibles (Fig. 24) bifid; *mds_1_* relatively long, piliform, located basally; *mds_2_* medium size, piliform, located distinctly apically and laterally. Maxilla (Fig. 27) stipes with very long *stps* and both *pfs*; very short to minute *mbs*, and sensillum close to *mbs*; mala with six medium sized finger-like *dms*; five *vms*, different in length, three setae medium size, and two setae very short. Maxillary palpi: basal palpomere with one short *mxps* and two sensilla; distal palpomere with, cuticular apical processes; length ratio of basal and distal palpomeres 1:0.8. Prelabium (Fig. 27) with one relatively long *prms*; ligula with two very short to minute *ligs*; premental sclerite broad, ring-shaped. Labial palpi with two palpomeres; length ratio of basal and distal palpomeres 1:0.8; each of the palpomeres with one sensillum, distal palpomere with short, cuticular apical processes. Postlabium (Fig. 27) with very short *pms_1_* located basally, very long *pms_2_* located medially and short to medium size *pms_3_* located apically; membranous area basolaterally distinctly asperate.

**Figures 28–30.**
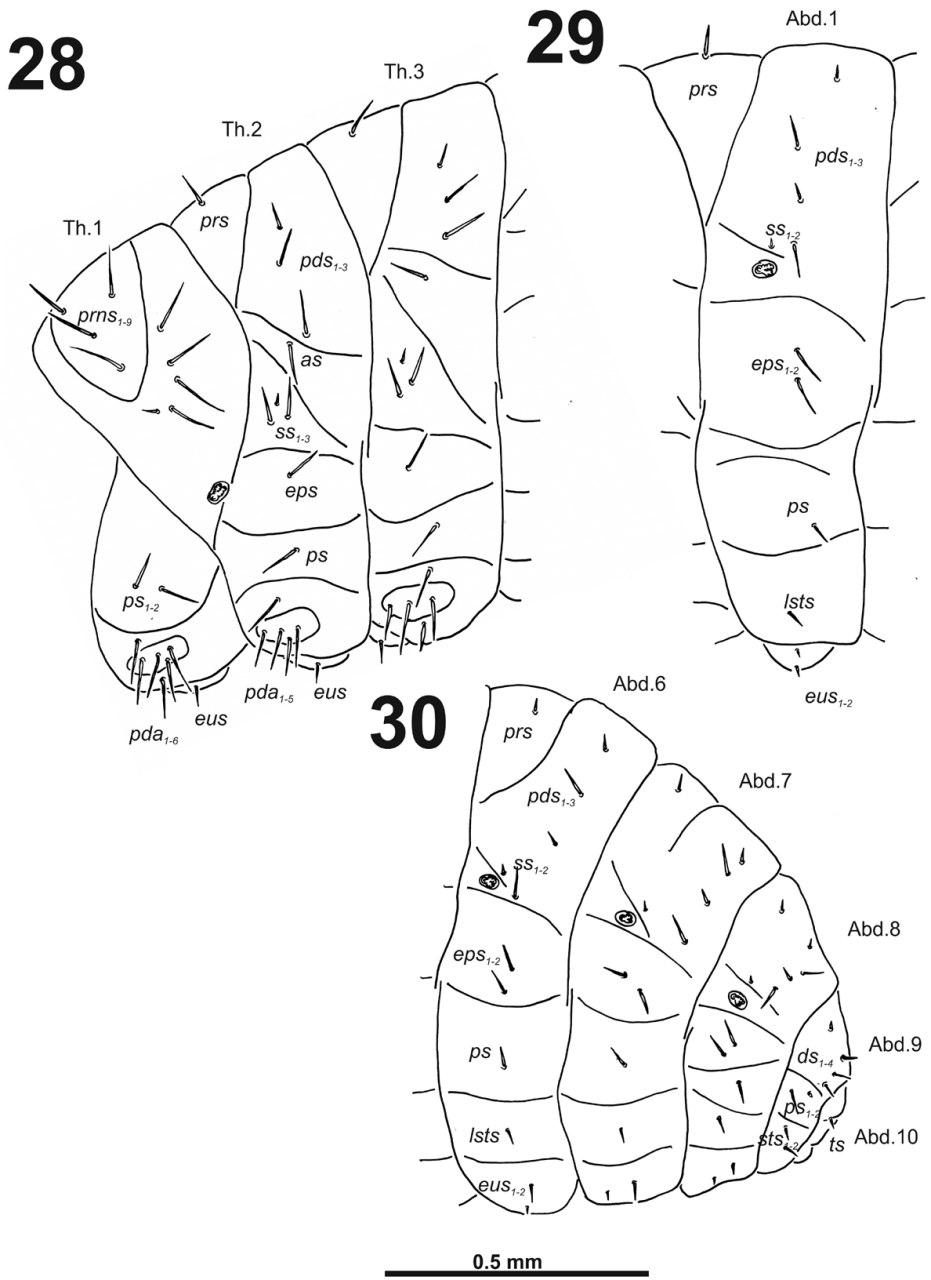
*Cleopomiaruslongirostris* mature larva, habitus. **28** Lateral view of thoracic segments **29** Lateral view of abdominal segment I **30** Lateral view of abdominal segments VI–X. Abbreviations: *as* – alar s., *ds* – dorsal s., *eps* – epipleural s., *eus* – eusternal s., *lsts* – laterosternal s., *pda* – pedal s., *pds* – postdorsal s., *prns* – pronotal s., *prs* – prodorsal s., *ss* – spiracular s., *ps* – pleural s., *sts* – sternal s., *ts* – terminal s., Th1–3 – number of thoracic segments, Ab1–10 – number of abdominal seg.

*Thorax.* Prothorax (Fig. 28) with eight very long to long and one very short to minute *prns*, small pigmented dorsal sclerite present with four long *prns*, this sclerite subdivided in two triangular plates medially; two very long to long *ps*; and one short to very short *eus.* Meso- and metathorax (Fig. 28) with one long *prs*, three medium to long *pds*; one very long to long *as*; two very long and one very short to minute *ss*; one long *eps*; one long *ps*; and one short to very short *eus.* Each pedal area of the thoracic segments with 5–6 very long to long *pda*.

*Abdomen.* Abdominal segments I–VII (Figs 29–30) with one short *prs*; one long and two short to very short *pds* (order: short, long, short); one long and one minute *ss*; two very long to long *eps*; one relatively long *ps*; one short *lsts*; and two very short *eus.* Abdominal segment VIII (Fig. 30) with one very short to minute *prs*; one relatively long and two very short *pds* (order: very short, long, very short); one relatively long and one minute *ss*; two relatively long *eps*; one short *ps*; one short *lsts*; and two very short *eus.* Abdominal segment IX (Fig. 30) with three relatively long and one very short to minute *ds*; one relatively long and sometimes one minute *ps*; and two short to very short *sts.* Abdominal segment X (Fig. 30) with one very short seta (*ts*).

###### Biology.

We can confirm that larvae of this species feed on seed capsules of *Campanulatrachelium* L., where they pupate without producing galls. It is noteworthy that adults did not exit by making a hole in the capsules but remained inside with the rostrum folded in the ventral canal until these opened spontaneously and forcefully, blowing up the seeds. On the other hand, it would be impossible, especially for the female, to straighten up the very long rostrum inside the capsule due to the limited available space. This is a more advantageous behaviour and apparently opposite to that of *Rhopalapionlongirostre* (Olivier, 1807), another species where the female rostrum is more than twice as long as the stout male rostrum. In this species, Wilhelm et al. (2011) argued that the long rostrum is presumably an advantage for this weevil because its larvae can feed on plant parts with high energy density into buds (i.e., pollen grains) and that natural selection favours rostrum elongation. However, these authors reported that the elongated rostrum of females also bears a high risk when metamorphosed weevils attempt to leave their site of pupal development, which is the dry seed chambers, and therefore mortality during escaping may counteract selection for rostrum elongation, thus placing a limit on rostrum exaggeration. It is noteworthy that *R.longirostre* does not possess a ventral canal, which allows it to retain the folded rostrum.

###### Remarks.

This species is only known from France, Italy, and Switzerland, where it is quite common. The adult is very closely related to *C.graminis*, as also supported by preliminary molecular studies (I Toševski, unpublished data), from which it differs only by the very long rostrum especially in the female and usually by the larger size (Caldara and Legalov 2016). Therefore, the larval differences between these two taxa, in *C.longirostris* antennae bearing one medium size conical sensorium and four sensilla (Fig. 23), dorsal setae (except *des_4_*) extremely long (Fig. 22), prothorax with eight very long and one very short to minute *prns* (Fig. 28), are very important since they allow easy separation of these two species.

##### Cleopomiarus medius

Taxon classificationAnimaliaColeopteraCurculionidae

(Desbrochers des Loges, 1893)

Figures 31 , 32 , 33–34 , 35–36 , 37 , 38–40

###### Material examined.

13 L3 larvae, ex seed capsules of *Campanulalingulata* Waldst. and Kit., 26.06.2017, Staničenje, Pirot, east Serbia, leg. I. Toševski, all collected in association with adults, det R. Caldara. Accession numbers of sequenced specimens: MH558547.

###### Description.

*Measurements* (in mm). Body length: 5.10–7.30 (mean 5.67). Body width (metathorax or abdominal segments I–II) up to 2.02. Head width: 0.83–0.96 (mean 0.91).

**Figure 31.**
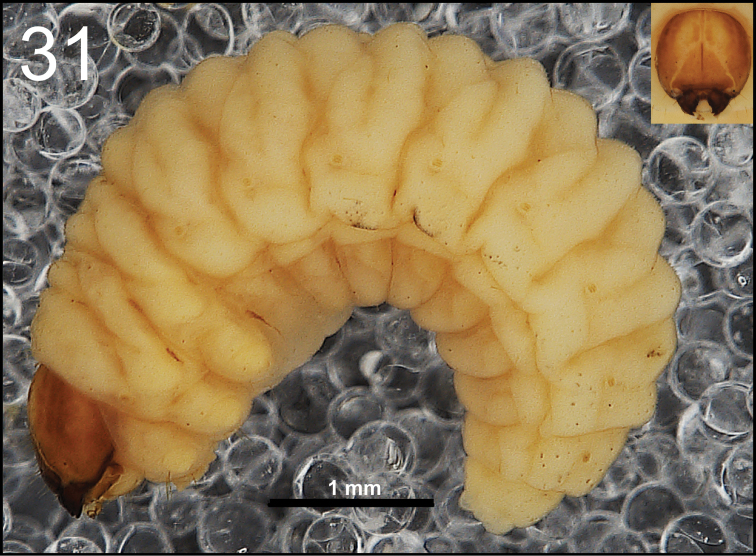
*Cleopomiarusmedius* mature larva habitus.

*General.* Body elongated, slender, weakly curved, rounded in cross section (Fig. 31).

*Colouration.* Pale brown or almost yellow head (Fig. 31). All thoracic and abdominal segments from white to slightly yellow (Fig. 31).

*Vestiture.* Setae on body thin, slightly from orange to brown, distinctly different in length (minute to very short or long). Cuticle distinctly asperate.

**Figure 32.**
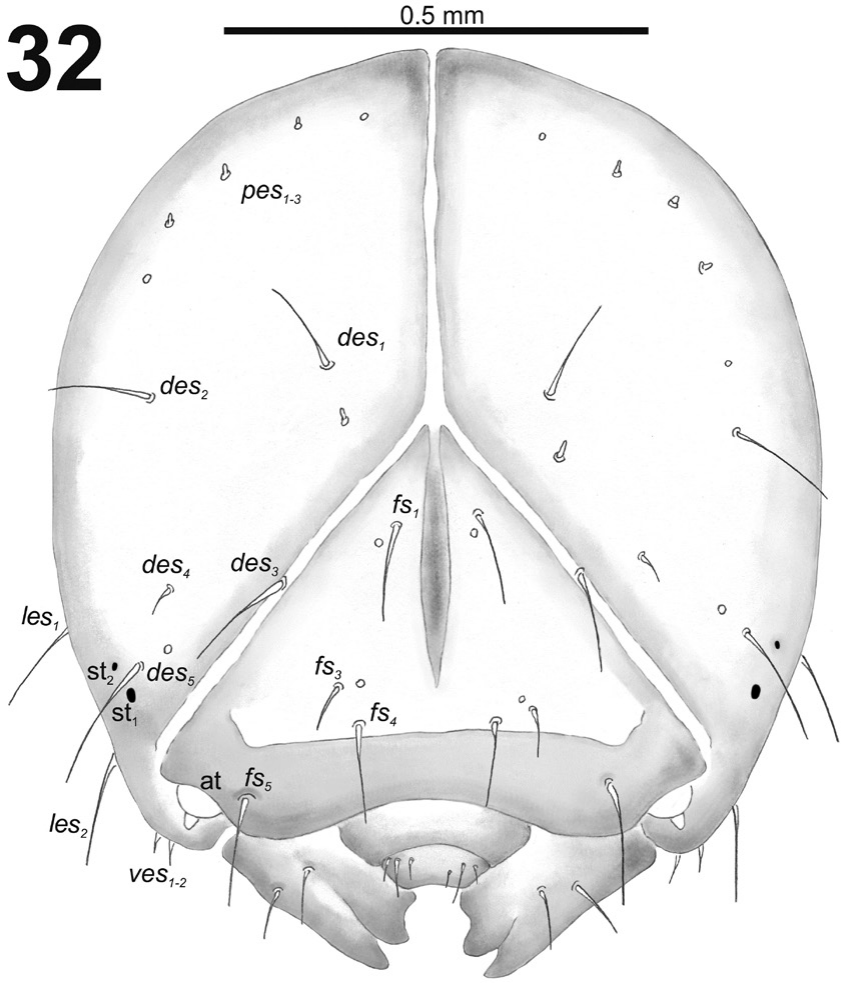
*Cleopomiarusmedius* mature larva head, frontal view. Abbreviations: *des* – dorsal epicranial s., *fs* – frontal epicranial s., *les* – lateral epicranial s., *pes* – postepicranial s., *ves* – ventral s., at – antenna, st – stemmata.

*Head capsule* (Fig. 32). Head suboval. Frontal sutures distinct. Endocarina distinctly widened in the middle of the length. Two small stemmata (st), located close to *des_5_*. *Des_1–2_* and *des_5_* very long; *des_3_* medium size; *des_4_* short (Fig. 32). *Fs_1_* long; *fs_2_* absent; *fs_3_* medium; *fs_4_* long; and *fs_5_* very long (Fig. 32). *Les_1_* and *les_2_* as long as *des_5_*; both *ves* very short. Epicranial area with two sensilla and three *pes* in line with *des_2_*.

**Figures 33–34.**
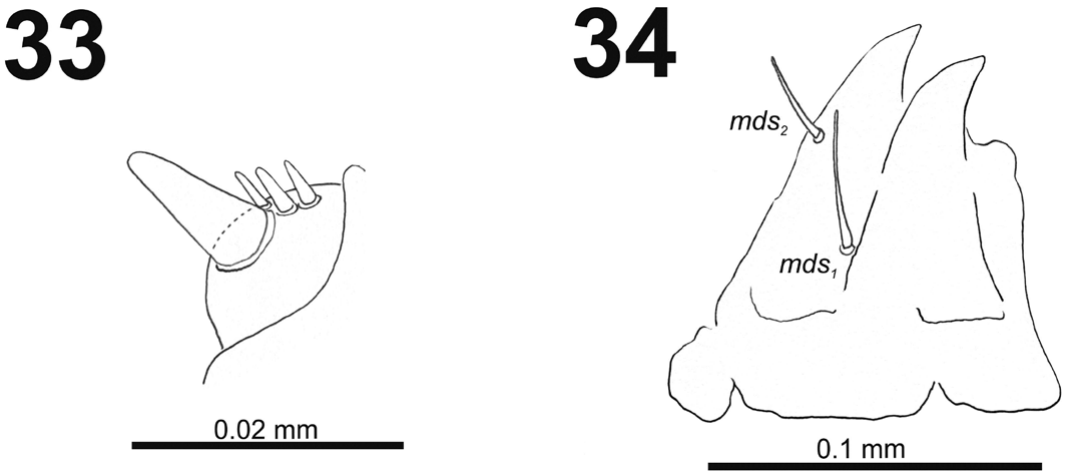
*Cleopomiarusmedius* mature larva. **33** Antenna **34** Right mandible. Abbreviation: *mds* – mandible dorsal s.

*Antennae* bearing one very long conical sensorium, and basal membranous article with three sensilla almost equal in length (Fig. 33).

**Figures 35–36.**
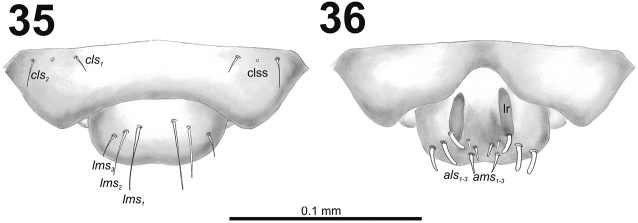
*Cleopomiarusmedius* mature larva, mouthparts. **35** Labrum and clypeus **36** Epipharynx. Abbreviations: *als* – anteriolateral s., *ams* – anteromedial s., *cls* – clypeal s., *lms* – labral s., *mes* – median s., clss – clypeal sensillum, lr – labral rods.

*Clypeus* (Fig. 35) approximately 4.25 times as wide as long with two almost equal in length *cls*: *cls_2_* some longer than *cls_1_*, and one sensillum; anterior margin sinuate.

**Figure 37.**
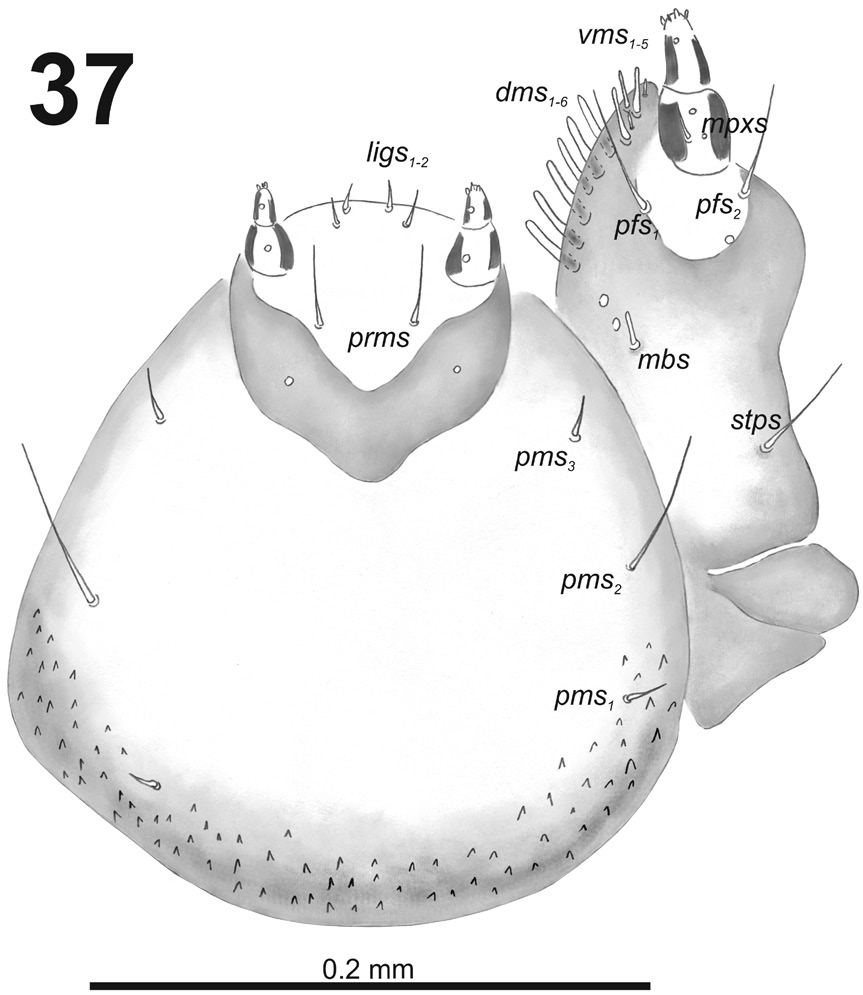
*Cleopomiarusmedius* larval mouthparts, maxillolabial complex, ventral view right maxilla. Abbreviations: *dms* – dorsal malar s., *vms* – ventral malar s., *mpxs* – maxillary palps s., *mbs* – basioventral s., *pfs* – palpiferal s., *stps* – stipital s., *prms* – premental s., *pms* – postmental s., *ligs* – ligular s.

*Mouthparts.* Labrum (Fig. 35) two times as wide as long, with three piliform *lms*, different in the length; *lms_1_* and *lms_2_* located medially, and *lms_3_* located anterolaterally; *lms_1_* very long and reaches distinctly the labrum margin, *lms_2_* long, and *lms_3_* short, three times as shorter than *lms_1_*. Epipharynx (Fig. 36) with three medium sized finger-like *als*, and three medium to short *ams* in different shape; labral rods (lr) distinct, elongated. Mandibles (Fig. 36) bifid; bearing with two setae in short to medium size, piliform, and aligned longitudinally, *mds_1_* located basally; *mds_2_*, located distinctly apically. Maxilla (Fig. 34) stipes with long *stps* and equal in length *pfs_1_* and *pfs_2_*, very short to minute *mbs*, and two sensilla close to *mbs*; mala with six medium sized finger-like *dms*; five *vms*, different in length, three setae medium size, and two setae very short. Maxillary palpi: basal palpomere with one short *mxps* and two sensilla; distal palpomere with cuticular apical processes; length ratio of basal and distal palpomeres 1:0.8. Prelabium (Fig. 37) with one short *prms*; ligula with two short *ligs*; premental sclerite broad, ring-shaped. Labial palpi with two palpomeres; length ratio of basal and distal palpomeres 1:0.9; each of the palpomeres with one sensillum, distal palpomere with short, cuticular apical processes. Postlabium (Fig. 37) with short *pms_1_* located basally, very long *pms_2_* located medially and short *pms_3_* located apically; membranous area basolaterally distinctly asperate.

**Figures 38–40.**
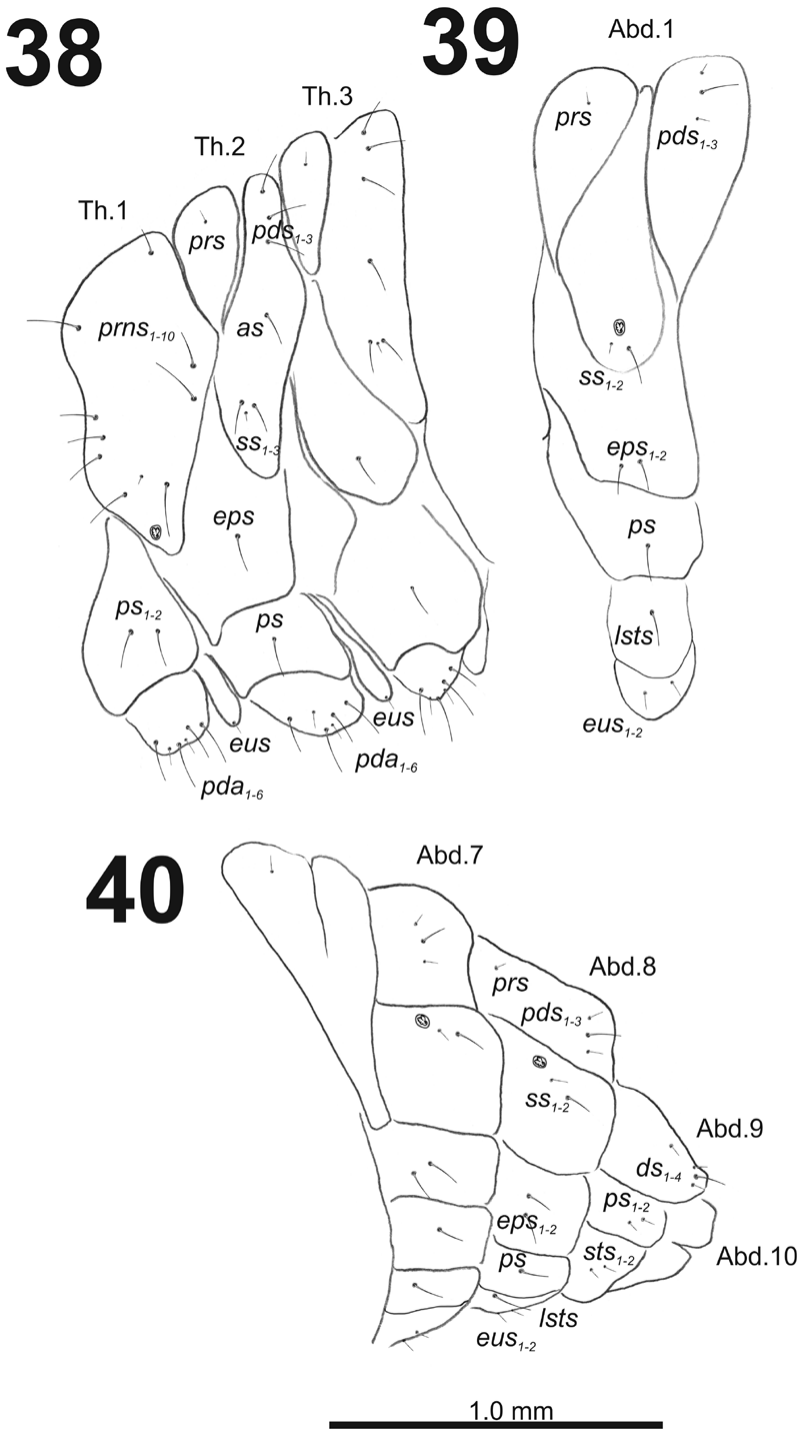
*Cleopomiarusmedius* mature larva, habitus. **38** Lateral view of thoracic segments **39** Lateral view of abdominal segment I **40** Lateral view of abdominal segments VI–X. Abbreviations: *as* – alar s., *ds* – dorsal s., *eps* – epipleural s., *eus* – eusternal s., *lsts* – laterosternal s., *pda* – pedal s., *pds* – postdorsal s., *prns* – pronotal s., *prs* – prodorsal s., *ss* – spiracular s., *ps* – pleural s., *sts* – sternal s., Th1–3 – number of thoracic segments, Ab1–10 – number of abdominal seg.

*Thorax.* Prothorax (Fig. 38) with nine long and one very short *prns*; two long *ps*; and one short *eus.* Meso- and metathorax (Fig. 38) with one short *prs*, three long *pds*; one long *as*; two long and one very short to minute *ss*; one long *eps*; one long *ps*; and one short *eus.* Each pedal area of the thoracic segments with six different in length *pda*.

*Abdomen.* Abdominal segments I–VII (Figs 39–40) with one very short *prs*; two short and one long *pds* (order: short, long, short); one long and one minute *ss*; two long *eps*; one long *ps*; one long *lsts*; and two short *eus.* Abdominal segment VIII (Fig. 40) with one very short *prs*; three *pds* (order: short, long, short); one long and one minute *ss*; two long *eps*; one long *ps*; one long *lsts*; and two short *eus.* Abdominal segment IX (Fig. 40) with one medium long and three very short *ds*; two short *ps*; and two short *sts.* Abdominal segment X (Fig. 40) without seta.

###### Biology.

Previously, the unique biological datum on this species was reported by Weill et al. (2011), who collected adults in Syria on *Michauxiacampanuloides* L’Hér., a small genus of Campanulaceae distributed in the Middle East, possibly a synonym of *Campanula* (Crowl et al. 2014). Therefore, the observation that this species feeds on *Campanulalingulata* Waldst. and Kit. is unpublished. Moreover, adults were recently observed feeding on flowers of *Campanulasibirica* L. in eastern Serbia (I Toševski, pers. obs.), but larval development on this plant species is not confirmed. Like *C.distinctus* and *C.graminis*, larvae are seed feeders inside capsules of the host plant without producing galls.

###### Remarks.

This species was previously known from Anatolia, Syria and many countries of the Balkans but not from Serbia. The adults of this species are characterized by a very long rostrum in the females. This character, however, is not uncommon in the Palaearctic *Cleopomiarus*. For example, this character is shared with *C.longirostris* and *C.distinctus*, two other taxa presented in this paper. It is distinguishable from these species by the less globose and moderately elongate elytra, and moreover by the shape of the male and female genitalia (Caldara and Legalov 2016). Other characters of the immatures allow easy separation of *C.medius* from these two species as well as from *C.graminis* (see keys to larvae and pupae). There are also substantial molecular differences between *C.medius* and other species (I Toševski, unpublished data).

##### Cleopomiarus meridionalis

Taxon classificationAnimaliaColeopteraCurculionidae

(H. Brisout, 1863)

Figures 41 , 42 , 43–44 , 45–46 , 47 , 48–50

###### Material examined.

10 L3 larvae: south-eastern France, Castellar (Menton), Juin 2005, ex seed capsules of *Campanularapunculus* L., leg. and det. R. Caldara all collected in association with adults.

###### Description.

*Measurements* (in mm). Body length: 2.20–3.15 (mean 2.8). Body width (metathorax or abdominal segments I–II) up to 0.73. Head width: 0.35–0.51 (mean 0.45).

**Figure 41.**
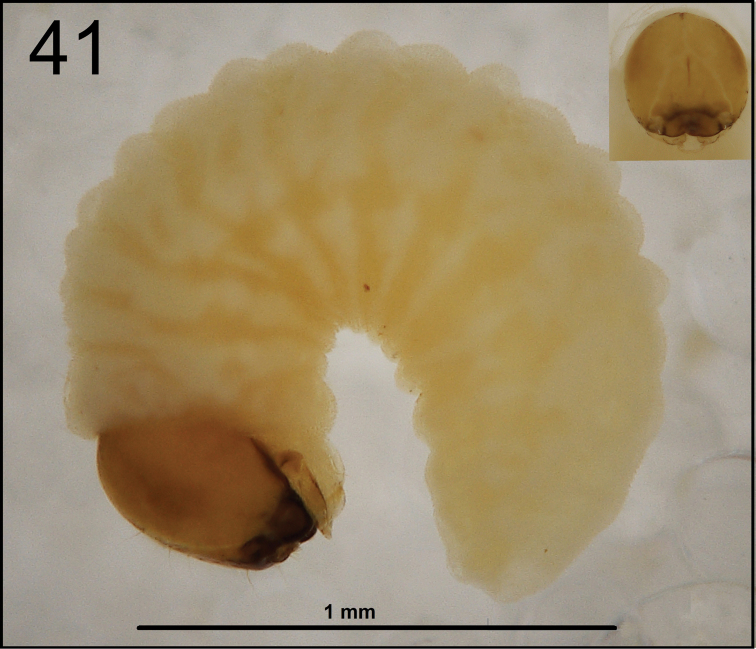
*Cleopomiarusmeridionalis* mature larva habitus.

*General.* Body elongated, slender, curved, rounded in cross section (Fig. 41).

*Colouration.* Pale brown or almost yellow head (Fig. 41). All thoracic and abdominal segments from distinctly white to slightly yellow (Fig. 41).

*Vestiture.* Setae on body thin, slightly from orange to pale brown, distinctly different in length (minute to very short or long to very long). Cuticle distinctly asperate.

**Figure 42.**
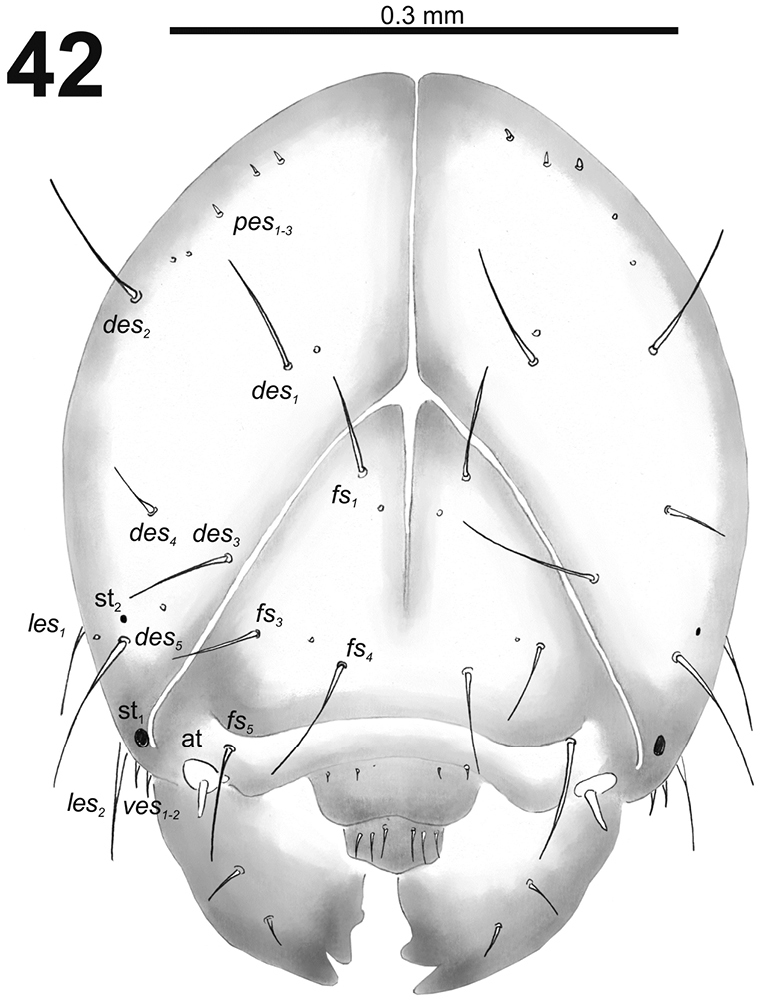
*Cleopomiarusmeridionalis* mature larva head, frontal view. Abbreviations: *des* – dorsal epicranial s., *fs* – frontal epicranial s., *les* – lateral epicranial s., *pes* – postepicranial s., *ves* – ventral s., at – antenna, st – stemmata.

*Head capsule* (Fig. 42). Head suboval, distinctly flattened laterally. Frontal sutures narrow, but distinct. Two stemmata (st), anterior one in the form of a small pigmented spot; and posterior one in form of a very small pigmented spot, located on each side close *des_5_*. *Des_1–3_* and *des_5_* very long; *des_4_* relatively long (Fig. 42). *Fs_1_* long to very long; *fs_2_* absent; *fs_3_* long medium, laterally to *fs_4_*; *fs_4_* very long; and *fs_5_* very long (Fig. 42). *Les_1_* and *les_2_* as long as *des_5_*; both *ves* medium size. Epicranial area with two sensilla and three *pes* in line with *des_2_*.

**Figures 43–44.**
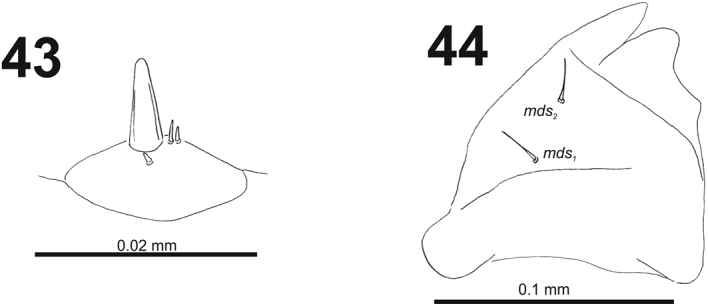
*Cleopomiarusmeridionalis* mature larva. **43** Antenna **44** Right mandible. Abbreviation: *mds* – mandible dorsal s.

*Antennae* bearing one very long conical sensorium, and basal membranous article with three sensilla different in length, two behind conical sensorium, and one ahead of it (Fig. 43).

**Figures 45–46.**
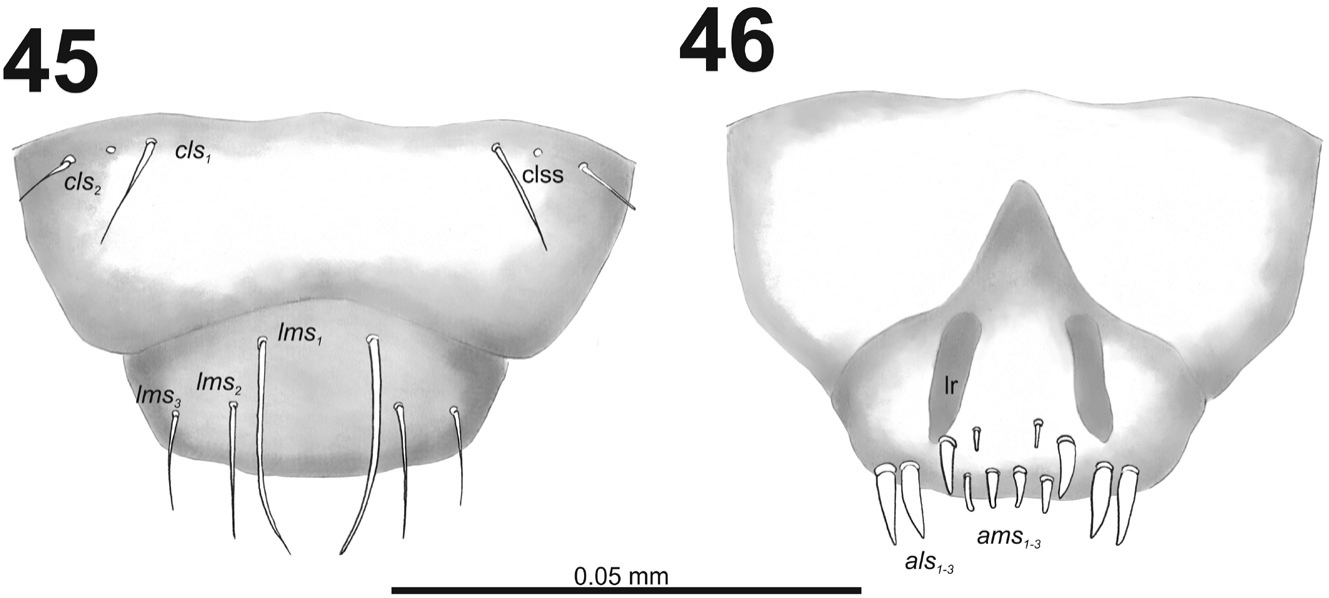
*Cleopomiarusmeridionalis* mature larva, mouthparts. **45** Labrum and clypeus **46** Epipharynx. Abbreviations: *als* – anteriolateral s., *ams* – anteromedial s., *cls* – clypeal s., *lms* – labral s., *mes* – median s., clss – clypeal sensillum, lr – labral rods.

*Clypeus* (Fig. 45) approximately three times as wide as long with two medium size *cls*, *cls_1_* distinctly longer than *cls_2_*, and one sensillum; anterior margin sinuate.

**Figure 47.**
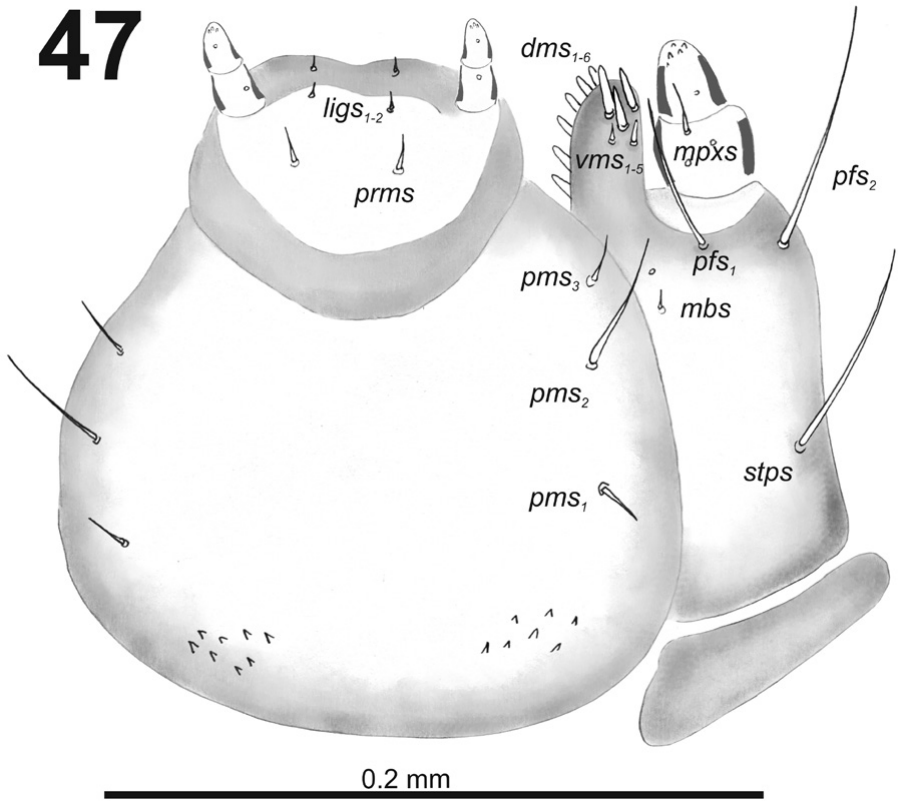
*Cleopomiarusmeridionalis* larval mouthparts, maxillolabial complex, ventral view right maxilla. Abbreviations: *dms* – dorsal malar s., *vms* – ventral malar s., *mpxs* – maxillary palps s., *mbs* – basioventral s., *pfs* – palpiferal s., *stps* – stipital s., *prms* – premental s., *pms* – postmental s., *ligs* – ligular s.

*Mouthparts.* Labrum (Fig. 45) less than 2.5 times as wide as long, with three piliform *lms*, different in the length; *lms_1_* located posteromedially, very close to margin of clypeus, *lms_2_* located in the middle, and *lms_3_* located laterally; *lms_1_* very long and reaches distinctly the labrum margin, *lms_2_* long, and *lms_3_* medium size, more than twice times as short as *lms_1_*. Epipharynx (Fig. 46) with three medium sized finger-like *als*, two *als* of identical in length, and the third one distinctly shorter and also located close to labral rods (lr); with three short *ams* in different shape, *ams_1_* and *ams_2_* piliform, finger-like *ams_3_* and enlarged in middle, and also located more close to lr; without *mes*; labral rods (lr) distinct, elongated, oval. Mandibles (Fig. 44) bifid; bearing with two setae in short to medium size, piliform, and aligned longitudinally., *mds_1_* located basally; *mds_2_*, located distinctly apically. Maxilla (Fig. 47) stipes with very long *stps* and *pfs_2_*, medium size *pfs_1_*, very short to minute *mbs*, and sensillum close to *mbs*; mala with six medium sized finger-like *dms*; five *vms*, different in length, four setae medium size, and one seta very short. Maxillary palpi: basal palpomere with one short *mxps* and two sensilla; distal palpomere with short, cuticular apical processes; length ratio of basal and distal palpomeres 1:0.8. Prelabium (Fig. 47) with one very short *prms*; ligula with two very short to minute *ligs*; premental sclerite broad, ring-shaped. Labial palpi with two palpomeres; length ratio of basal and distal palpomeres 1:0.9; each of the palpomeres with one sensillum, distal palpomere with short, cuticular apical processes. Postlabium (Fig. 47) with short *pms_1_* located basally, long *pms_2_* located medially and short *pms_3_* located apically; membranous area basolaterally only a partly and finely asperate.

**Figures 48–50.**
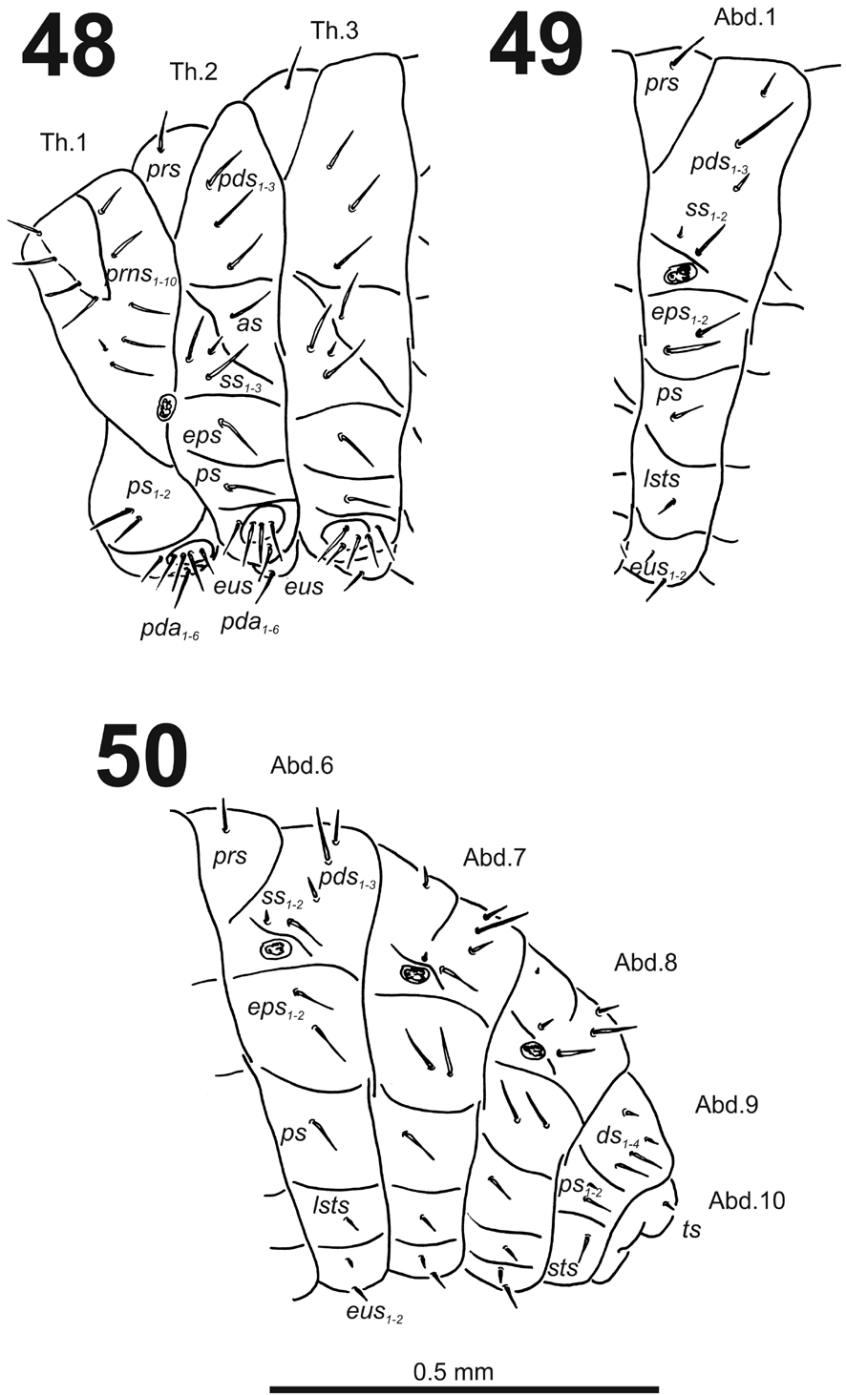
*Cleopomiarusmeridionalis* mature larva, habitus. **48** Lateral view of thoracic segments **49** Lateral view of abdominal segment I **50** Lateral view of abdominal segments VI–X. Abbreviations: *as* – alar s., *ds* – dorsal s., *eps* – epipleural s., *eus* – eusternal s., *lsts* – laterosternal s., *pda* – pedal s., *pds* – postdorsal s., *prns* – pronotal s., *prs* – prodorsal s., *ss* – spiracular s., *ps* – pleural s., *sts* – sternal s., *ts* – terminal s., Th1–3 – number of thoracic segments, Ab1–10 – number of abdominal seg.

*Thorax.* Prothorax (Fig. 48) with nine very long and one very short to minute *prns*, small pigmented dorsal sclerite present with four long *prns*, this sclerite subdivided in two triangular plates medially; two very long to long *ps*; and one short *eus.* Meso- and metathorax (Fig. 48) with one long *prs*, three very long to long *pds*; one long *as*; two very long and one very short to minute *ss*; one long *eps*; one very long to long *ps*; and one short to very short *eus.* Each pedal area of the thoracic segments with 5–6 very long *pda*.

*Abdomen.* Abdominal segments I–VII (Figs 49–50) with one long *prs*; two relatively long to short and one very long to long *pds* (order: relatively long, very long, short); one very long to long and one minute *ss*; two very long *eps*; one very long to long *ps*; one relatively long to short *lsts*; and one short to very short and one relatively long *eus.* Abdominal segment VIII (Fig. 50) with sometimes one very short to minute *prs*; one short and one long to relatively long *pds* (order: short, long); one long and one minute *ss*; two very long *eps*; one very long to long *ps*; one relatively long to short *lsts*; and one short to very short and one relatively long *eus.* Abdominal segment IX (Fig. 50) with two relatively long and two short to very short *ds*; one relatively long and one minute *ps*; and one relatively long *sts.* Abdominal segment X (Fig. 50) with one very short seta (*ts*).

###### Biology.

Adults of this species are usually collected on the flowers of *Campanularapunculus* L., and we can confirm that larvae feed on seeds of this plant as previously reported by Hoffmann (1958).

###### Remarks and comparative notes.

This species is widely distributed and common in southern Europe, whereas it appears rare in North Africa and the Middle East. Adults can be confused with some related species such as *C.plantarum* (Germar, 1823), *C.micros* (Germar, 1821) and *C.reitteri* (Caldara & Legalov, 2016), from which they differ by some external characters and the shape of their genitalia (Caldara and Legalov 2016). In contrast, this species is poorly related morphologically to the other species of *Cleopomiarus* studied here. This difference is confirmed also by the larval morphology, which differs from all of the other species mainly by a longer *fs_3_* that is almost as long as *fs_4_*.

##### Genus Miarus

Taxon classificationAnimaliaColeopteraCurculionidae

Schoenherr, 1826

###### Description.

*Measurements* (in mm). Body length: 3.80–8.39. Body width (metathorax or abdominal segments I–II) 1.55–2.04. Head width: 0.57–0.83.

*General.* Body slender, C-curved, rounded in cross section.

*Colouration.* From black to dark brown head. All thoracic and abdominal segments yellowish, with some asperities.

*Vestiture.* Setae on body thin, in different colouration, distinctly different in length; piliform.

*Head capsule*. Head almost rounded, sometimes slightly flattened laterally, endocarinal line present and distinct, more than half the length of frons. Frontal sutures on the head narrow and loosened, but distinct, and ever extended to the antennae. One stemma (st), in the form of a pigmented spot with convex cornea. Dorsum of the epicranium with four or five setae; *des_3_* located anteriorly on epicranium close border with frontal suture. Frons with three or four setae; *fs_2_* absent. Head also with two *les* and two *ves.* Epicranial area with two or three *pes* and more or without sensilla.

*Antennae* located at the end of the frontal suture on each side, membranous and distinctly convex basal article bearing one very long conical sensorium; basal membranous article with 1–4 sensilla.

*Clypeus* transverse-shaped, approximately 2.5–3.5 times as wide as long with two *cls*, and one sensillum (clss) between setae; all very close to margin with frons.

*Mouthparts.* Labrum with three piliform *lms*; anterior margin bisinuate. Epipharynx with three finger-like *als*; with two *ams*; and 0–2 *mes*; labral rods (lr) elongated. Mandibles distinctly broad, bifid, teeth of unequal height; slightly truncate; both setae piliform and located apically. Maxilla stipes with one *stps*, two *pfs* and one *mbs* and one sensillum; mala with six finger-like *dms*, in two sizes, first or first and second *dms* very long as *pfs*, next medium length; five *vms*; all *vms* distinctly shorter than *dms.* Maxillary palpi with two palpomeres; basal palpomere with one short *mxps* and two sensilla; distal palpomere with one sensillum and a group of micro cuticular apical processes. Prelabium oval-shaped, with one *prms*; ligula with sinuate margin and 2–3 *ligs*; premental sclerite feebly visible. Labial palpi with two palpomeres; each of the palpomeres with one sensillum, distal palpomere with cuticular apical processes. Postlabium with three *pms*, all located laterally.

*Thorax.* Prothorax slightly smaller than meso- and metathorax. Spiracle bicameral, placed between the pro- and mesothorax (see, e.g., Skuhrovec et al. 2015). Prothorax with ten *prns*; two *ps*; and one *eus.* Mesothorax with one *prs*, three *pds*; one *as*; two long and one short *ss*; one *eps*; one *ps*; and one *eus.* Chaetotaxy of metathorax almost identical to that of mesothorax. Each pedal area of the thoracic segments well separated, with 5–6 *pda*.

*Abdomen.* Abdominal segments I–III of almost equal length, next abdominal segments decreasing gradually to the terminal parts of the body. Abdominal segment X reduced to four anal lobes of unequal size, the lateral lobes being distinctly the largest, the dorsal and the ventral lobe very small. Anus located terminally. Eight spiracles, bicameral, all spiracles placed medially or anteromedially and functional. Abdominal segments I–VIII with one *prs* (sometimes abdominal segment VIII without); three *pds*, *pds_2_* the longest one; one long and one minute *ss*; two long *eps*; one *ps*; one *lsts*; and two *eus.* Abdominal segment IX with 3–4 *ds*; 1–3 *ps*; and two *sts.* Abdominal segment X with one minute seta present or absent.

##### Miarus abnormis

Taxon classificationAnimaliaColeopteraCurculionidae

Solari, 1947

Figures 51 , 52 , 53–54 , 55–56 , 57 , 58–60

###### Material examined.

5 L3 larvae: north-eastern Italy, Venezia Giulia, Duino (Trieste), Rilke path, August 2017, ex galls on capsules of *Campanulapyramidalis* L., leg. E. Tomasi, all collected in association with adults, det. R. Caldara.

###### Description.

*Measurements* (in mm). Body length: 3.50–4.75 (mean 3.9). Body width (abdominal segment II) up to 1.65. Head width: 0.57–0.65 (mean 0.60).

**Figure 51.**
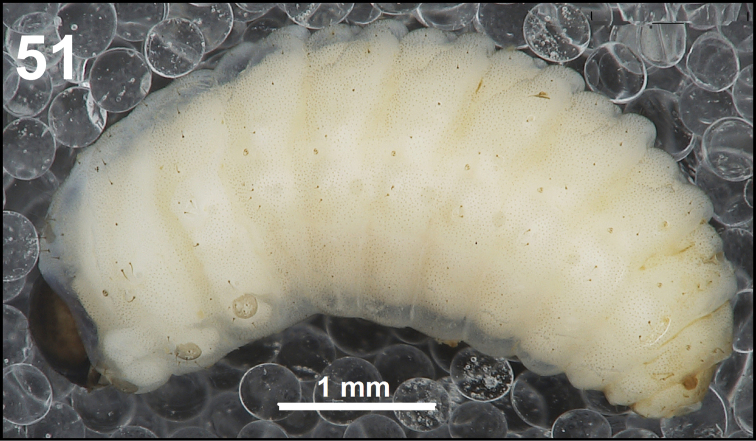
*Miarusabnormis* mature larva habitus.

*General.* Body moderately elongated, rather stout, curved, rounded in cross section (Fig. 51).

*Colouration.* Almost black head (Fig. 51). All thoracic and abdominal segments from greyish-white to yellowish; prodorsum with brownish dorsal sclerite; all abdominal segments covered with fine spiculation (Fig. 51).

*Vestiture.* Setae on body thin, brown, rather short or minute, piliform.

**Figure 52.**
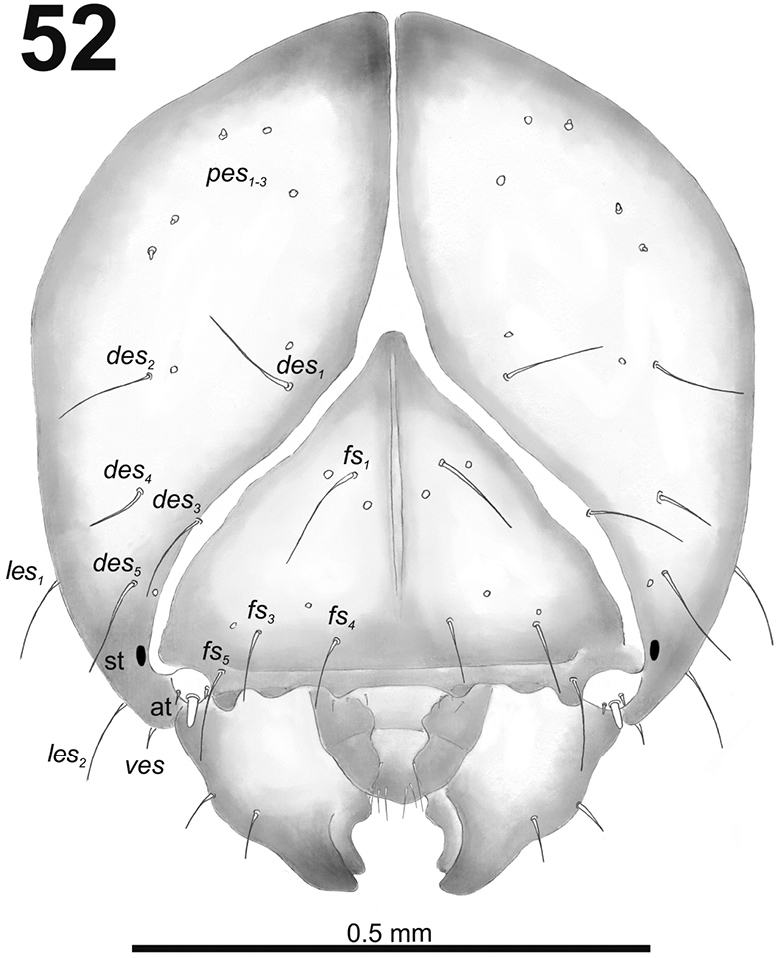
*Miarusabnormis* mature larva head, frontal view. Abbreviations: *des* – dorsal epicranial s., *fs* – frontal epicranial s., *les* – lateral epicranial s., *pes* – postepicranial s., *ves* – ventral s., at – antenna, st – stemmata.

*Head capsule* (Fig. 52). Head oval, slightly flattened laterally. Endocarinal line present and very distinct. Stemma placed below *des_5_*. *Des_1–3_* and *des_5_* long; *des_4_* medium size (Fig. 52). *Fs_1_* long; *fs_2_* absent; *fs_3_* and *fs_4_* medium size; and *fs_5_* long (Fig. 52). *Les_1_* and *les_2_* as long as *des_5_*; one *ves* very short. Epicranial area with three *pes* (in line with *des_2_*), and also two sensilla.

**Figures 53–54.**
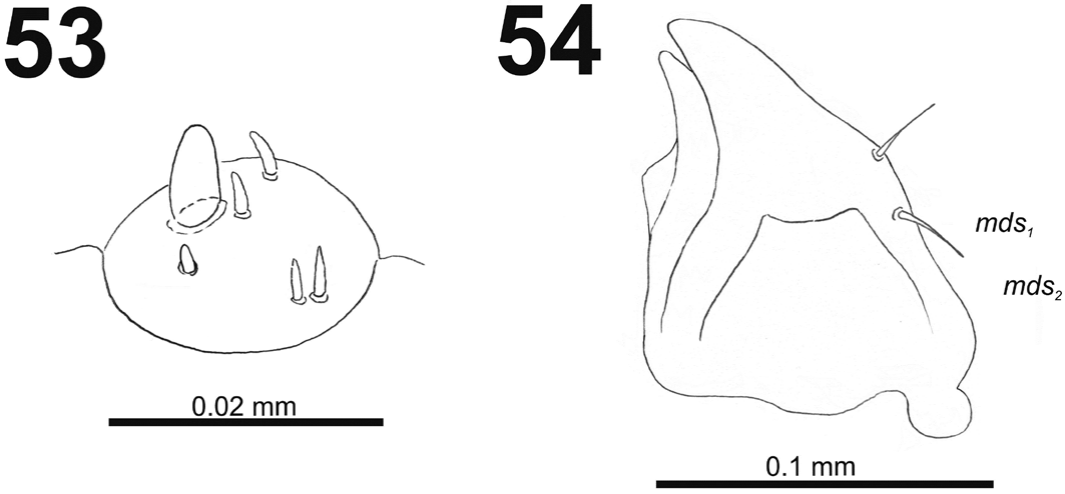
*Miarusabnormis* mature larva. **53** Antenna **54** Right mandible. Abbreviation: *mds* – mandible dorsal s.

*Antennae* located at the end of the frontal suture on each side, membranous and distinctly convex basal article bearing one conical sensorium, relatively elongated; basal membranous article with four sensilla (styloconica) equal in length, and one (ampullacae) (Fig. 53).

**Figures 55–56.**
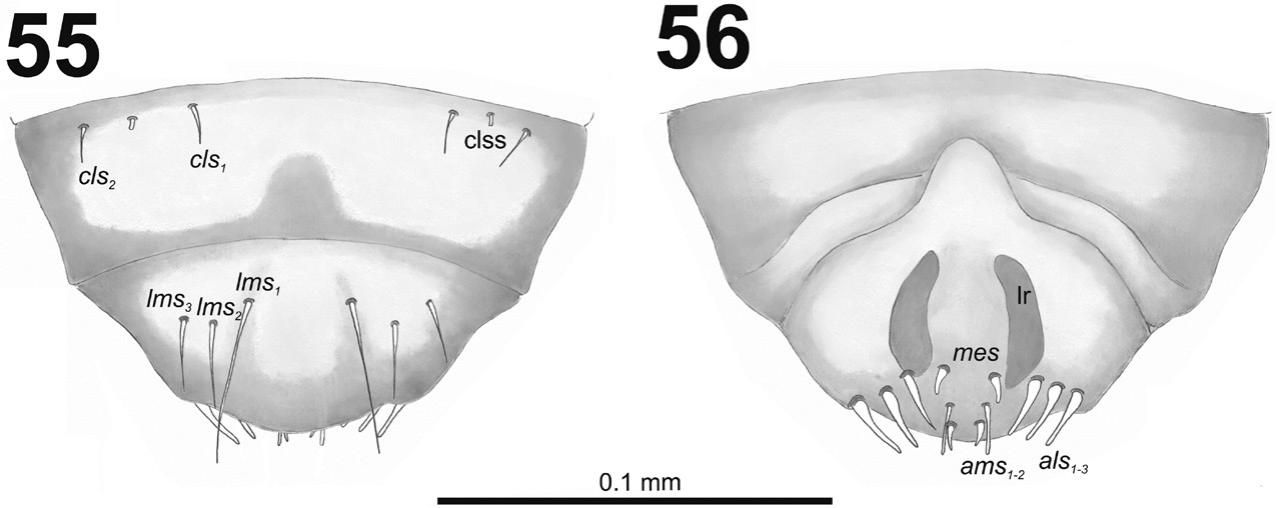
*Miarusabnormis* mature larva, mouthparts. **55** Labrum and clypeus **56** Epipharynx. Abbreviations: *als* – anteriolateral s., *ams* – anteromedial s., *cls* – clypeal s., *lms* – labral s., *mes* – median s., clss – clypeal sensillum, lr – labral rods.

*Clypeus* (Fig. 55) trapezium-shaped, approximately 3.3 times as wide as long with two medium size, equal in length *cls*, and one sensillum (clss) between setae; all very close to margin with frons; anterior margin of clypeus rounded to inside.

**Figure 57.**
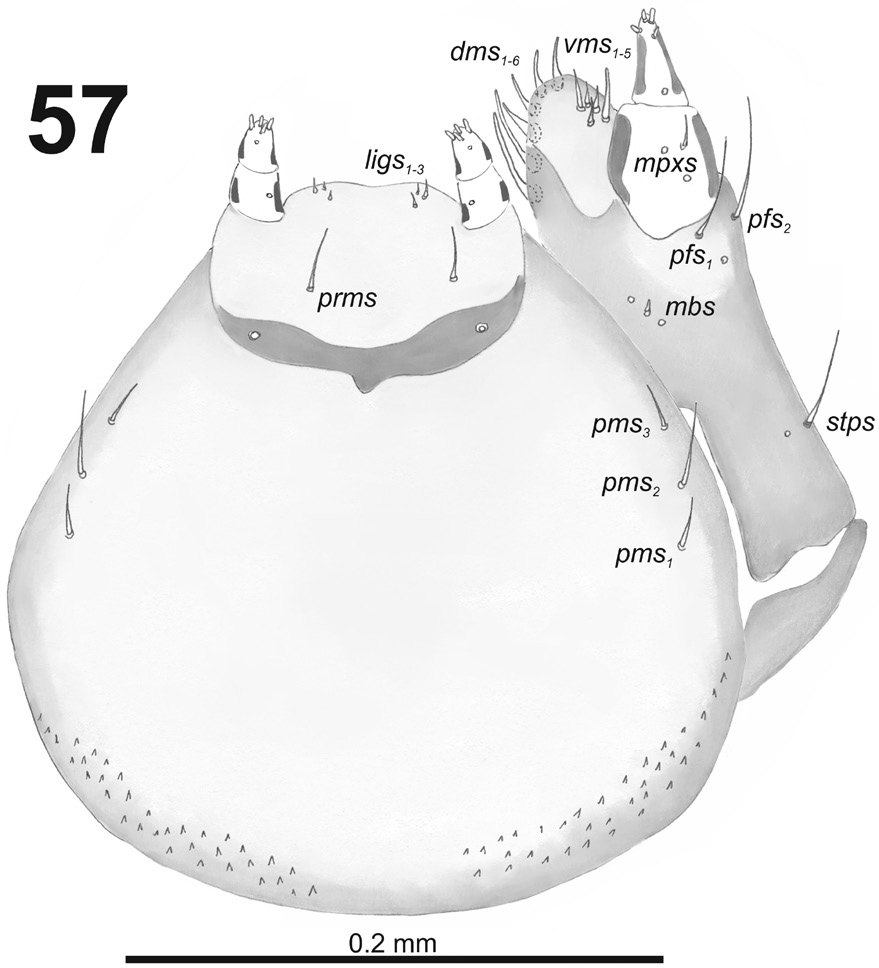
*Miarusabnormis* larval mouthparts, maxillolabial complex, ventral view right maxilla. Abbreviations: *dms* – dorsal malar s., *vms* – ventral malar s., *mpxs* – maxillary palps s., *mbs* – basioventral s., *pfs* – palpiferal s., *stps* – stipital s., *prms* – premental s., *pms* – postmental s., *ligs* – ligular s.

*Mouthparts.* Labrum (Fig. 55) 2.5 times as wide as long, with three piliform *lms*, *lms_1_* twice longer than (equal in the length) *lms_2_* and *lms_3_*; all located more or less anteromedially, all reach labral margin; anterior margin double sinuate. Epipharynx (Fig. 56) with three medium sized finger-like *als*, all similar in length; with two rather short, different in length *ams*; and one medium size, finger-like *mes*; labral rods (lr) distinct, kidney-shaped. Mandibles (Fig. 54) distinctly broad, bifid, teeth of unequal height; slightly truncate; cutting edge with a blunt tooth; bearing with two setae in short size, piliform, and aligned longitudinally. Maxilla (Fig. 57): stipes with long *stps*, two long *pfs*, one minute *mbs* and two sensillae close to *mbs*; mala with six finger-like *dms* (first and second elongated, forth to sixth medium size); five *vms* (two medium size and three very short); all *vms* shorter than *dms.* Maxillary palpi with two palpomeres; basal palpomere with one short *mxps* and two sensilla; length ratio of basal and distal palpomeres almost 1:1; distal palpomere with one sensillum and a group of microcuticular processes apically. Prelabium (Fig. 57) oval-shaped, with one medium *prms*; ligula with sinuate margin and three minute *ligs*; premental sclerite narrow, ring-shaped, well visible. Labial palpi with one palpomere (partially seems as two palpomere); palpomere with one sensillum and medium, cuticular apical processes. Postlabium (Fig. 57) with three *pms*, all located laterally; *pms_1_* and *pms_3_* short, *pms_2_* medium size; membranous area basolaterally finely asperate.

**Figures 58–60.**
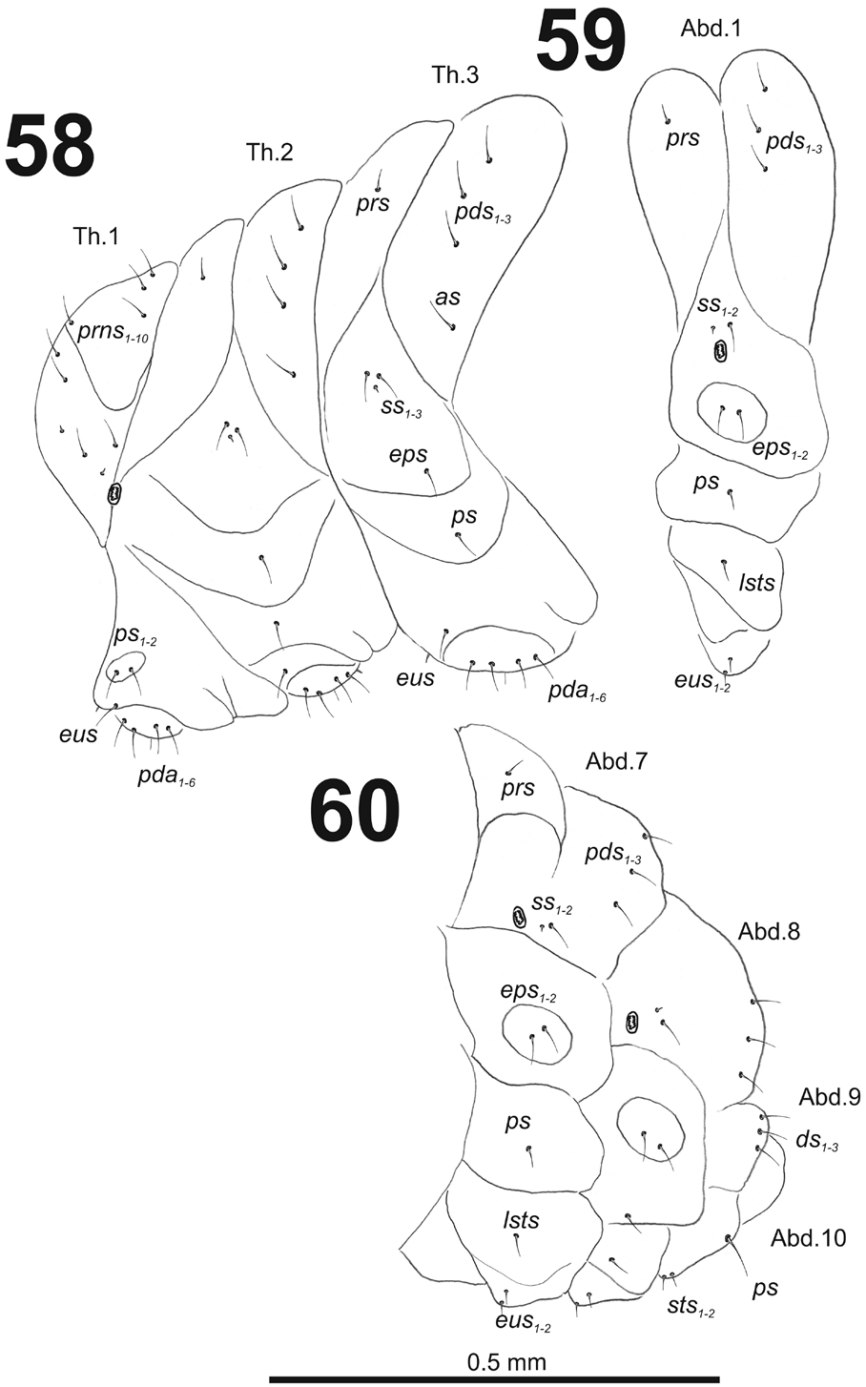
*Miarusabnormis* mature larva, habitus. **58** Lateral view of thoracic segments **59** Lateral view of abdominal segment I **60** Lateral view of abdominal segments VII–X. Abbreviations: *as* – alar s., *ds* – dorsal s., *eps* – epipleural s., *eus* – eusternal s., *lsts* – laterosternal s., *pda* – pedal s., *pds* – postdorsal s., *prns* – pronotal s., *prs* – prodorsal s., *ss* – spiracular s., *ps* – pleural s., *sts* – sternal s., *ts* – terminal s., Th1–3 – number of thoracic segments, Ab1–10 – number of abdominal seg.

*Thorax.* Prothorax smaller than meso- and metathorax. Spiracle bicameral, placed between the pro- and mesothorax. Prothorax (Fig. 58) with ten *prns* (two minute and eight long), well pigmented dorsal sclerite with four long *prns*); two medium *ps*; and one short *eus.* Meso- and metathorax (Fig. 58) with one short *prs*, three medium *pds*; one medium *as*; two medium and one minute *ss*; one medium *eps*; one medium *ps*; and one very short *eus.* Chaetotaxy of metathorax (Fig. 58) almost identical to that of mesothorax. Each pedal area of the thoracic segments with six medium length *pda* (four of them placed on well-separated pedal areas, next two setae outside).

*Abdomen.* Abdominal segments I–VII (Figs 59, 60) with one short *prs*; three medium size *pds* (equal in length); one medium and one minute *ss*; two medium *eps*; one medium *ps*; one medium *lsts*; and two very short *eus.* Abdominal segment VIII (Fig. 60) without *prs*; three medium *pds*; one medium and one minute *ss* (sometimes absent); two medium *eps*; one medium *ps*; one medium *lsts*; and two very short *eus.* Abdominal segment IX (Fig. 60) with three medium *ds*; one relatively long *ps*; and two short *sts.* Abdominal segment X (Fig. 60) without seta.

###### Biology.

The only detailed biological data are reported by Tomasi (2002), who observed that this species lives in Friuli-Venezia Giulia (Italy) on *Campanulapyramidalis* L., where larvae cause a distinct swelling of the calix of the flowers, which remain closed.

###### Remarks.

This species has a well-delimited distribution (south-eastern Poland, Austria, north-eastern Italy, Slovenia, Croatia, Serbia, Montenegro, Macedonia). It is easily distinguishable from all other species of *Miarus* by the shape of the body of the penis, which is characterized by the presence of two lateral flanges at its apex. However, for the external morphology, the *M.abnormis* adults are very similar to several other species, such as *M.ajugae* and *M.campanulae*, from which they can be distinguished only by the characters of the male ventrite five (fovea less deep, teeth less robust). Unfortunately, the females of these three species appear not to be distinguishable (Caldara 2007), and the molecular fragment COI poorly differentiates these species. Therefore, the differences between the immatures of these species are much important for the separation of these three species. According to the larval morphology, *M.abnormis* appears more closely related to *M.ajugae* than to *M.campanulae* due to several features (mala with six finger-like *dms*, different in length: two setae elongated, and four setae of medium length; epipharynx with 1–2 *mes*, and finally *des_4_* and *fs_3_* present), confirming what was suggested by the adult morphology (Caldara 2007). Moreover, larvae of *M.abnormis* differ from other *Miarus* species here studied mainly by an epipharynx with one finger-like *mes*.

##### Miarus ajugae

Taxon classificationAnimaliaColeopteraCurculionidae

(Herbst, 1795)

Figures 61 , 62 , 63–64 , 65–66 , 67 , 68–70

###### Material examined.

26 L3 larvae: 9 exx., 12.07.2009, Bychawa ad Lublin, CE Poland, leg. E. Szwaj, det. J. Łętowski; 12 exx, ex galls on capsules of *Adenophoraliliifolia*, 30.06.2017, Kaludjerske Bare, Mt. Tara, Central Serbia, leg. I. Toševski, det. R. Caldara; 5 exx, ex galls on capsules of *Campanulabononiensis* L., 14.07.2017, Zavojsko jezero, Pirot, east Serbia, leg. I. Toševski, all collected in association with adults, det. R. Caldara. Accession numbers of sequenced specimens: MH558548.

###### Description.

*Measurements* (in mm). Body length: 4.50–8.39 (mean 5.70). Body width (metathorax or abdominal segments I–II) up to 2.04. Head width: 0.68–0.83 (mean 0.70).

**Figure 61.**
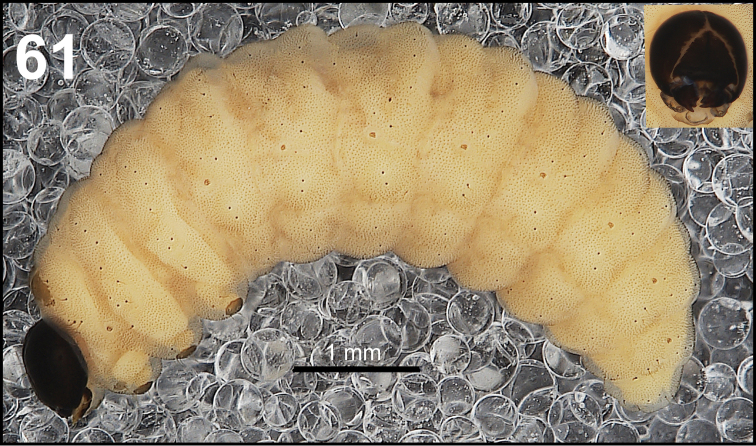
*Miarusajugae* mature larva habitus.

*General.* Body slender, C-curved, rounded in cross section (Fig. 61).

*Colouration.* Head dark brown to black (Fig. 61). All thoracic and abdominal segments yellowish with fine speculation, with clearly separated dark pigmented pedal areas (Fig. 61).

*Vestiture.* Setae on body very thin, piliform, distinctly different in length (minute to very short or long to very long).

**Figure 62.**
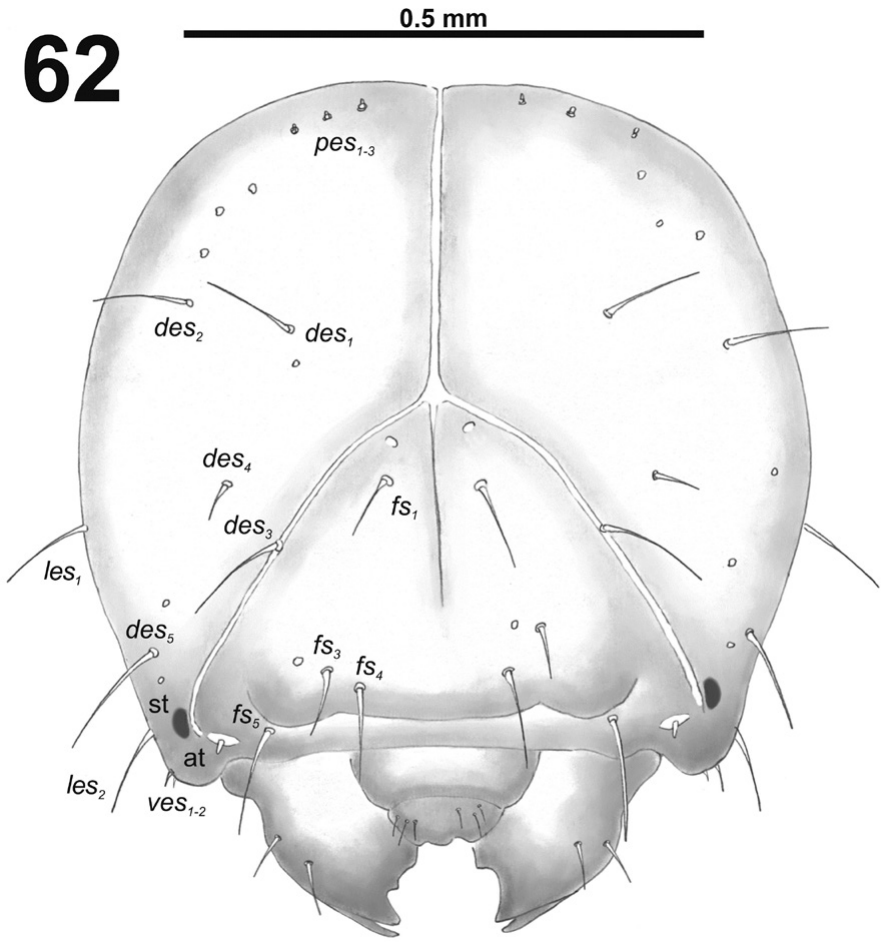
*Miarusajugae* mature larva head, frontal view. Abbreviations: *des* – dorsal epicranial s., *fs* – frontal epicranial s., *les* – lateral epicranial s., *pes* – postepicranial s., *ves* – ventral s., at – antenna, st – stemmata.

*Head capsule* (Fig. 62). Head almost rounded. Frontal sutures narrow and loosened, but distinct. One stemma (st), in the form of a large pigmented spot. *Des_1–3_* and *des_5_* long; *des_4_* very short (Fig. 62). *Fs_1_* long; *fs_2_* absent; *fs_3_* short; *fs_4_* and *fs_5_* long (Fig. 62). *Les_1_* and *les_2_* as long as *des_5_*; both *ves* very short. Epicranial area with three very short *pes* and also three sensilla.

**Figures 63–64.**
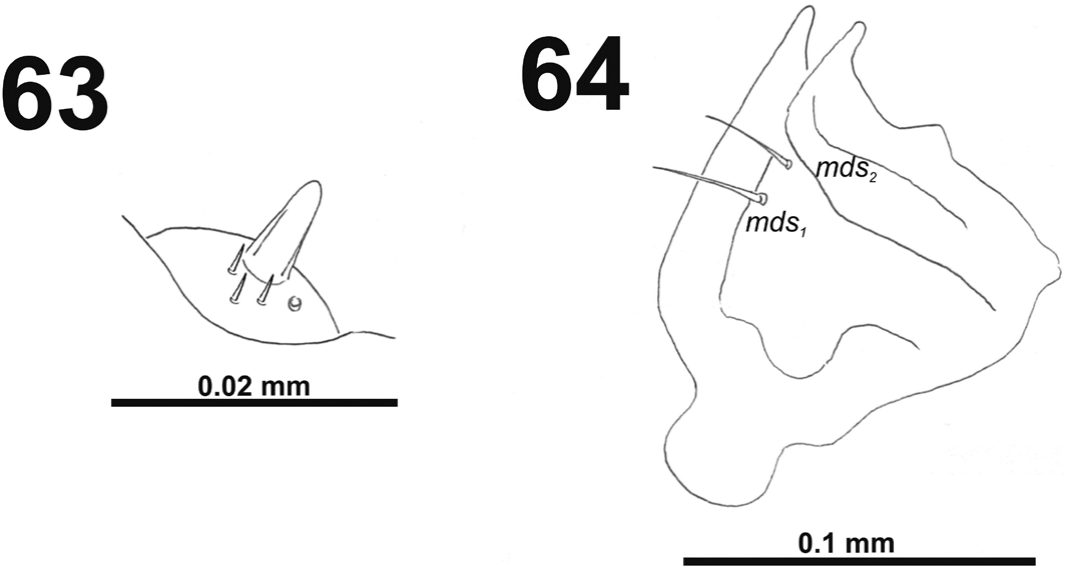
*Miarusajugae* mature larva. **63** Antenna **64** Right mandible. Abbreviation: *mds* – mandible dorsal s.

*Antennae* bearing one very long conical sensorium, and basal membranous article with three sensilla and one pore (Fig. 63).

**Figures 65–66.**
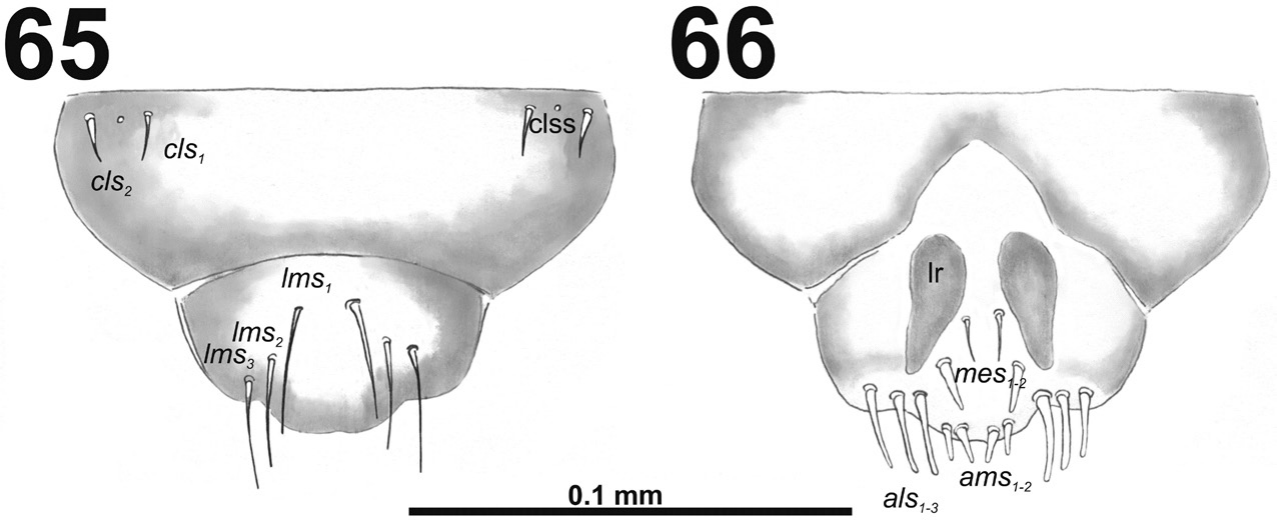
*Miarusajugae* mature larva, mouthparts. **65** Labrum and clypeus **66** Epipharynx. Abbreviations: *als* – anteriolateral s., *ams* – anteromedial s., *cls* – clypeal s., *lms* – labral s., *mes* – median s., clss – clypeal sensillum, lr – labral rods.

*Clypeus* (Fig. 65) approximately 3.5 times as wide as long with two short, almost equal in length *cls*, and one sensillum between them; anterior margin sinuate.

**Figure 67.**
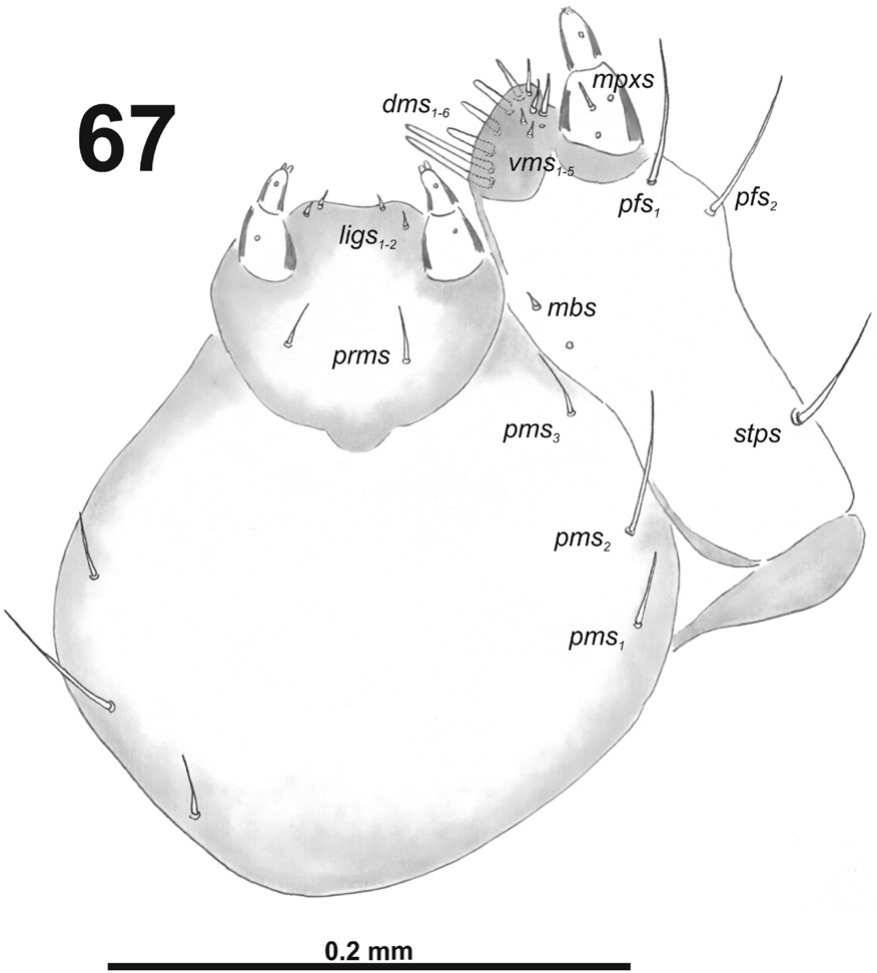
*Miarusajugae* larval mouthparts, maxillolabial complex, ventral view right maxilla. Abbreviations: *dms* – dorsal malar s., *vms* – ventral malar s., *mpxs* – maxillary palps s., *mbs* – basioventral s., *pfs* – palpiferal s., *stps* – stipital s., *prms* – premental s., *pms* – postmental s., *ligs* – ligular s.

*Mouthparts.* Labrum (Fig. 65) 1.6 times as wide as long, with three piliform *lms*, rather equal in length; *lms_1_* located medially, *lms_2_* located anteromedially, and *lms_3_* located anterolaterally; all of them reaches labral margin. Epipharynx (Fig. 66) with three long finger-like *als*, all of identical in length; with two medium size *ams*; and two *mes*, first finger-like, second sharp and more slender; labral rods (lr) elongated, broad, slightly convergent posteriorly. Mandibles (Fig. 64) bifid; cutting edge with small blunt tooth; bearing with two setae in medium to long size, piliform, located apically and aligned longitudinally. Maxilla (Fig. 67) stipes with long *stps* and both *pfs*, very short to minute *mbs*, and one sensillum close to *mbs*; mala with six finger-like *dms*, different in length: first and second very long, forth to sixth medium size; five *vms*, different in length, three setae medium size, and two setae very short. Maxillary palpi: basal palpomere with one short *mxps* and two sensilla; distal palpomere with cuticular apical processes; length ratio of basal and distal palpomeres 1:0.9. Prelabium (Fig. 67) with one medium *prms*; ligula with two minute *ligs*; premental sclerite narrow, ring-shaped. Labial palpi with two palpomeres; length ratio of basal and distal palpomeres 1:0.7; each of the palpomeres with one sensillum, distal palpomere with cuticular apical processes. Postlabium (Fig. 67) with short *pms_1_* located basally, long *pms_2_* located medially and short *pms_3_* located apically; membranous area without any asperities.

**Figures 68–70.**
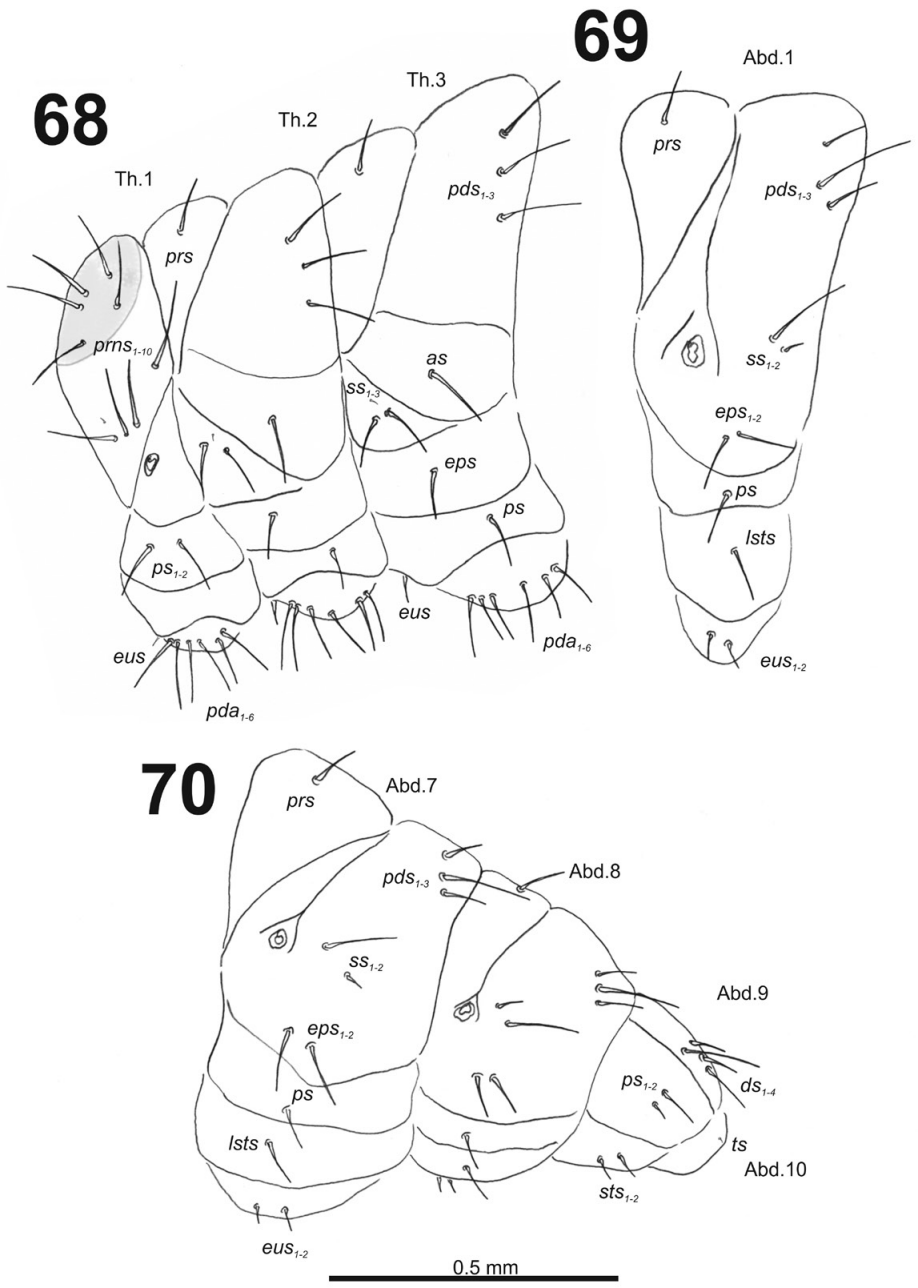
*Miarusajugae* mature larva, habitus. **68** Lateral view of thoracic segments **69** Lateral view of abdominal segment I **70** Lateral view of abdominal segments VII–X. Abbreviations: *as* – alar s., *ds* – dorsal s., *eps* – epipleural s., *eus* – eusternal s., *lsts* – laterosternal s., *pda* – pedal s., *pds* – postdorsal s., *prns* – pronotal s., *prs* – prodorsal s., *ss* – spiracular s., *ps* – pleural s., *sts* – sternal s., *ts* – terminal s., Th1–3 – number of thoracic segments, Ab1–10 – number of abdominal seg.

*Thorax.* Prothorax (Fig. 68) with ten *prns* (nine very long and one minute), small pigmented dorsal sclerite present with five long *prns*, this sclerite subdivided in two triangular plates medially; two long *ps*; and one short *eus.* Meso- and metathorax (Fig. 68) with one medium *prs*, three long *pds*; one very long *as*; two long and one minute *ss*; one long *eps*; one long *ps*; and one short *eus.* Chaetotaxy of metathorax (Fig. 68) almost identical to that of mesothorax. Each pedal area of the thoracic segments with six very long *pda*.

*Abdomen.* Abdominal segments I–VII (Figs 69–70) with one medium *prs*; two medium and one long to very long *pds* (order: medium, very long, medium); one very long and one minute *ss*; two long *eps*; one medium *ps*; one medium *lsts*; and two short *eus.* Abdominal segment VIII (Fig. 70) with one short *prs*; two short and one long *pds* (order: short, long, short); one long and one minute *ss*; two medium *eps*; one medium *ps*; one medium *lsts*; and two short *eus.* Abdominal segment IX (Fig. 70) with two relatively long and two short *ds*; two different in length *ps*; and two short *sts.* Abdominal segment X (Fig. 70) with one very short seta (*ts*).

###### Biology.

*Miarusajugae* was collected on various species of the genus *Campanula* (*C.carpathica* Jacq., *C.glomerata* L., *C.latifolia* L., *C.macrorrhiza* Gay ex DC, *C.media* L., *C.patula* L., *C.persicifolia* L., *C.rapunculoides* L., *C.rapunculus* L., *C.rhomboidalis* L., *C.rotundifolia* L., *C.trachelium* L.) and *Phyteuma* (*P.orbiculare* L., *P.spicatum* L.) (Caldara 2007). However, it was never reported previously to feed on *Campanulabononiensis* L. and *Adenophoraliliifolia* (L.) A. DC. (see also Biology of *C.graminis*).

###### Remarks.

This species with large Palaearctic distribution (from France and north-western Africa along all Europe to the Far East) is very closely related to *M.campanulae*, from which it differs mainly by the shape of the apex of the body of the penis (Caldara 2007). Unfortunately, molecular studies on the fragment COI revealed poor differences between these two species (Vahtera and Muona 2006; Hendrich et al. 2015, I. Toševski, unpublished data). Therefore, the consistent differences which we found in larval morphology between *C.ajugae* and *C.campanulae* are very important in order to confirm the validity of these two taxa at species level. Larvae of *M.ajugae* differ from all other species mainly by an epipharynx with two *mes*, first finger-like, second sharp and slender.

##### Miarus campanulae

Taxon classificationAnimaliaColeopteraCurculionidae

(Linnaeus, 1767)

Figures 71 , 72 , 73–74 , 75–76 , 77 , 78–80

###### Material examined.

9 L3 larvae: 30.07.1939, ex *Campanula*, Store Dyrehave, Denmark, leg. J.P. Kryger, collected in association with adults, det. Van Emden, coll. British Museum of Natural History (London).

###### Description.

*Measurements* (in mm). Body length: 3.80–5.96 (mean 5.20). Body width (metathorax or abdominal segments I–II) up to 1.61. Head width: 0.58–0.64 (mean 0.61).

**Figure 71.**
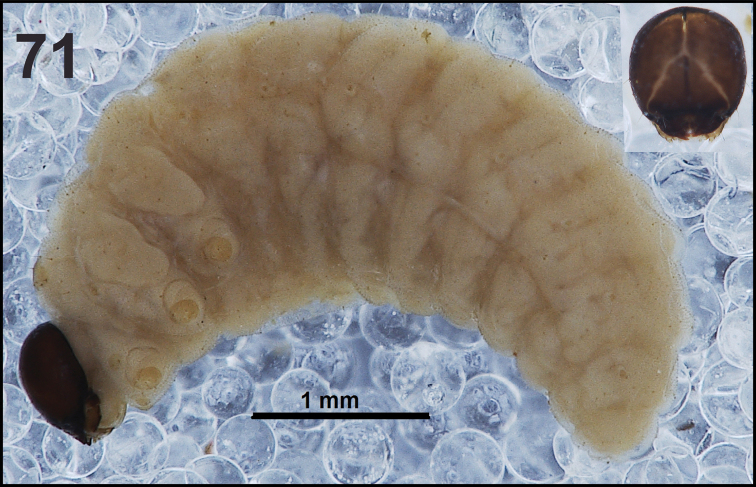
*Miaruscampanulae* mature larva habitus.

*General.* Body slender, C-curved, rounded in cross section (Fig. 71).

*Colouration.* Brown to dark brown head (Fig. 71). All thoracic and abdominal segments yellowish with distinct asperation (Fig. 71).

*Vestiture.* Setae on body thin, slightly from dark orange to brown, distinctly different in length (minute to very short or long to very long) distinct asperate.

**Figure 72.**
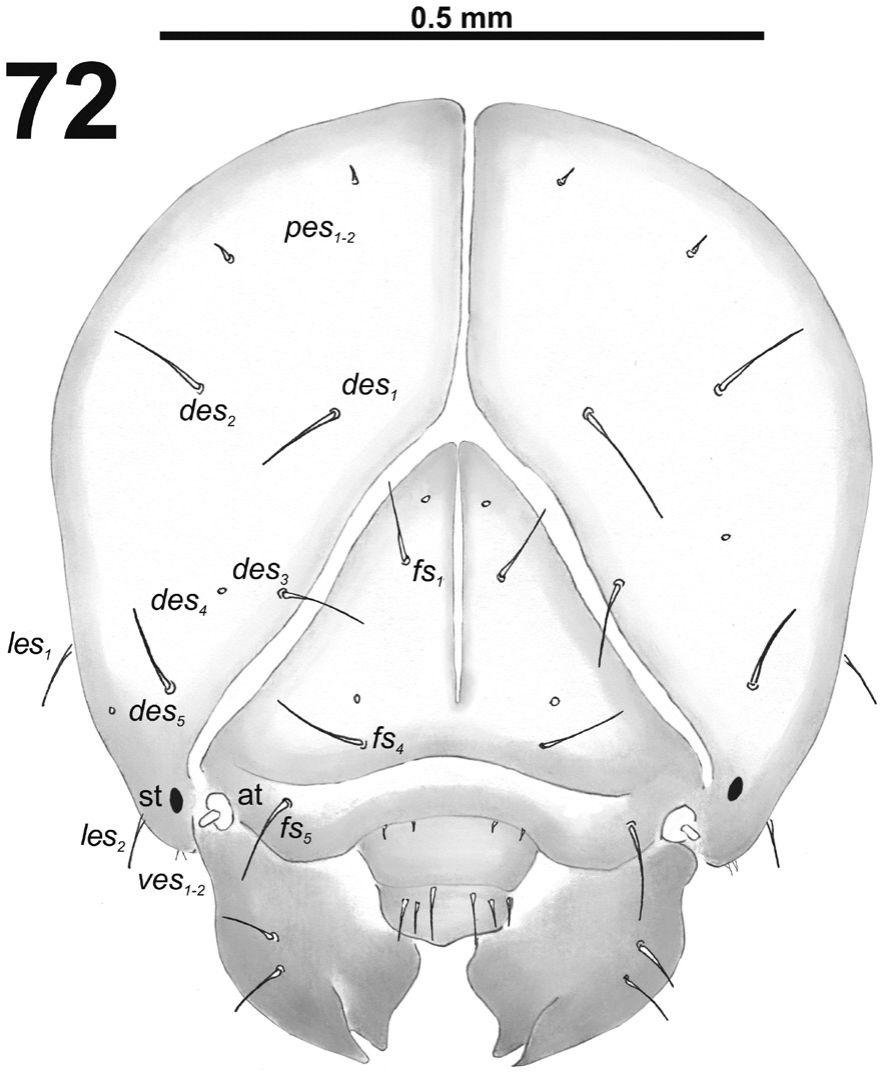
*Miaruscampanulae* mature larva head, frontal view. Abbreviations: *des* – dorsal epicranial s., *fs* – frontal epicranial s., *les* – lateral epicranial s., *pes* – postepicranial s., *ves* – ventral s., at – antenna, st – stemmata.

*Head capsule* (Fig. 72). Head almost round, very slightly flattened laterally. Frontal sutures narrow and loosened, but distinct. One stemma (st), in the form of a large pigmented spot. *Des_1–3_* and *des_5_* long; *des_4_* absent (Fig. 72). *Fs_1_* long; *fs_2_* and *fs_3_* absent; *fs_4_* and *fs_5_* long (Fig. 72). *Les_1_* and *les_2_* as long as *des_5_*; both *ves* short. Epicranial area with two *pes*.

**Figures 73–74.**
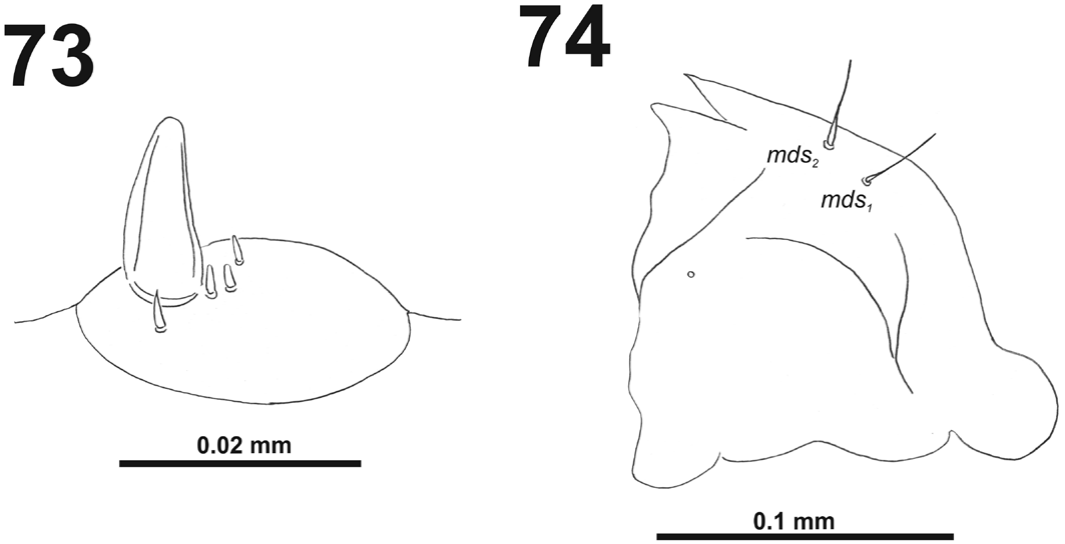
*Miaruscampanulae* mature larva. **73** Antenna **74** Right mandible. Abbreviation: *mds* – mandible dorsal s.

*Antennae* bearing one very long conical sensorium, and basal membranous article with four sensilla different in length, three behind conical sensorium, and one ahead of it (Fig. 73).

**Figures 75–76.**
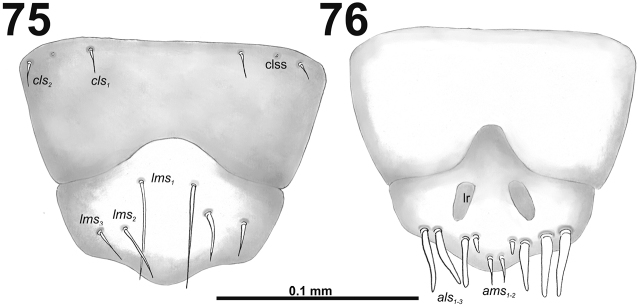
*Miaruscampanulae* mature larva, mouthparts. **75** Labrum and clypeus **76** Epipharynx. Abbreviations: *als* – anteriolateral s., *ams* – anteromedial s., *cls* – clypeal s., *lms* – labral s., *mes* – median s., clss – clypeal sensillum, lr – labral rods.

*Clypeus* (Fig. 75) approximately 2.5–3 times as wide as long with two short to very short *cls*, localized posterolaterally, *cls_1_* slightly longer than *cls_2_*, and one sensillum between them; anterior margin sinuate.

**Figure 77.**
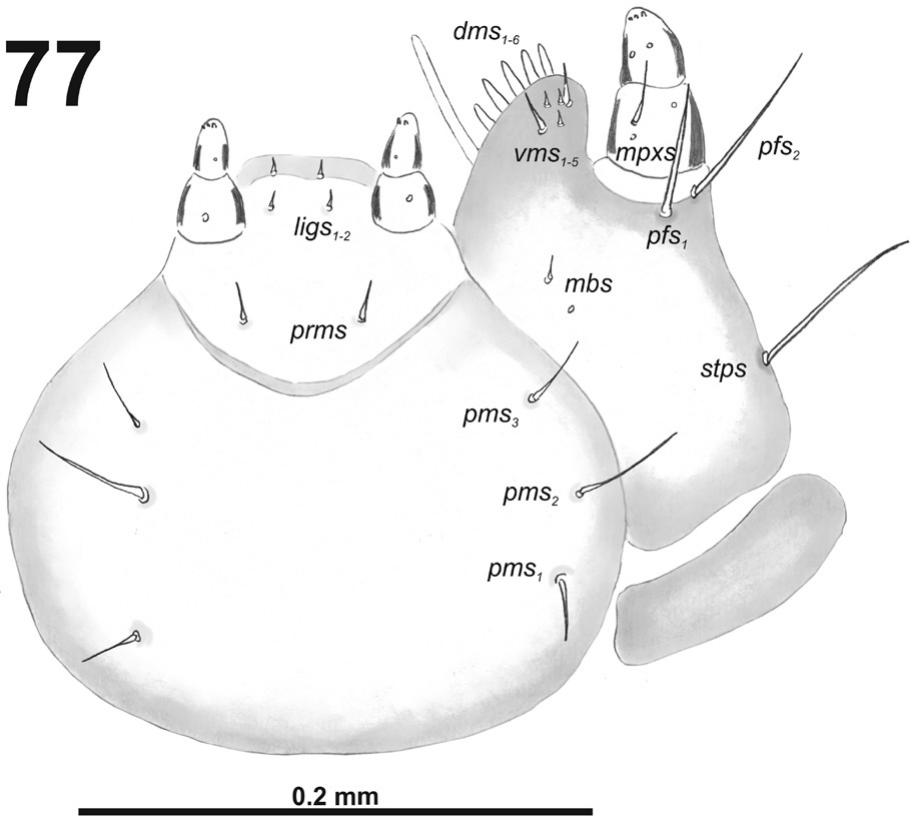
*Miaruscampanulae* larval mouthparts, maxillolabial complex, ventral view right maxilla. Abbreviations: *dms* – dorsal malar s., *vms* – ventral malar s., *mpxs* – maxillary palps s., *mbs* – basioventral s., *pfs* – palpiferal s., *stps* – stipital s., *prms* – premental s., *pms* – postmental s., *ligs* – ligular s.

*Mouthparts.* Labrum (Fig. 75) less than two times as wide as long, with three piliform *lms*, different in the length; *lms_1_* located medially, *lms_2_* located anteromedially, and *lms_3_* located anterolaterally; *lms_1_* long, *lms_2_* medium size, and *lms_3_* distinctly shorter than the previous two; no one distinctly reaches labral margin. Epipharynx (Fig. 76) with three long finger-like *als*, all of identical in length; with two *ams* in different length, *ams_1_* piliform of medium size, and finger-like short *ams_2_*; without *mes*; labral rods (lr) indistinct, slightly elongated, oval. Mandibles (Fig. 74) bifid; bearing with two setae of medium to long size, piliform, both located apically. Maxilla (Fig. 77) stipes with very long *stps* and *pfs_1_*, long *pfs_2_*, very short to minute *mbs*, and one sensillum close to *mbs*; mala with six finger-like *dms*, different in lengths: first very long (as long as *pfs_1_*), next medium size; five *vms*, different in length, two setae medium size, and three setae very short. Maxillary palpi: basal palpomere with one short *mxps* and two sensilla; distal palpomere with short, cuticular apical processes; length ratio of basal and distal palpomeres 1:0.9. Prelabium (Fig. 77) with one short *prms*; ligula with two very short to minute *ligs*; premental sclerite narrow, ring-shaped. Labial palpi with two palpomeres; length ratio of basal and distal palpomeres 1:0.8; each of the palpomeres with one sensillum, distal palpomere with short, cuticular apical processes. Postlabium (Fig. 77) with short *pms_1_* located basally, long *pms_2_* located medially and short *pms_3_* located apically; membranous area without any asperities.

**Figures 78–80.**
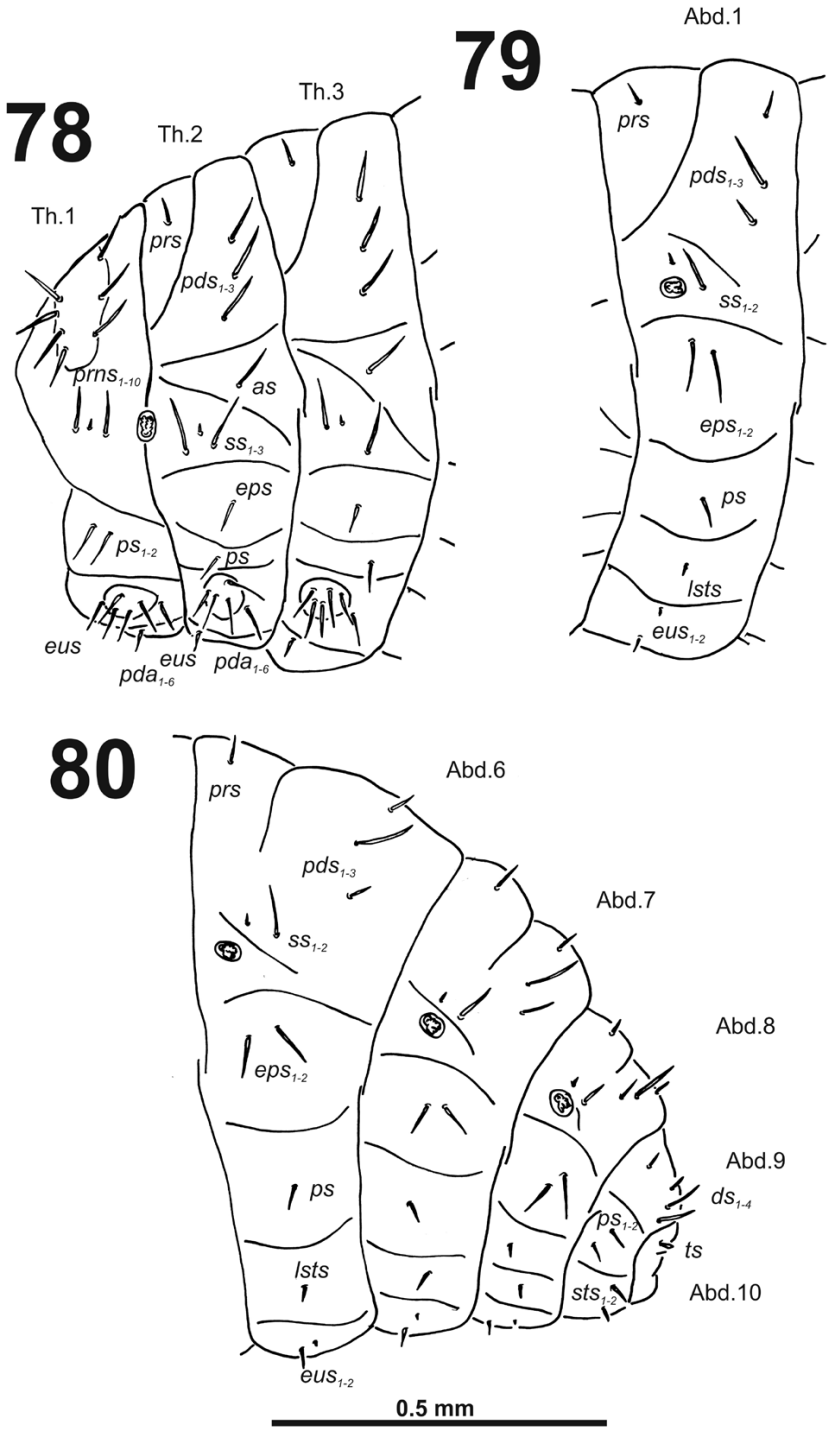
*Miaruscampanulae* mature larva, habitus. **78** Lateral view of thoracic segments **79** Lateral view of abdominal segment I **80** Lateral view of abdominal segments VI–X. Abbreviations: *as* – alar s., *ds* – dorsal s., *eps* – epipleural s., *eus* – eusternal s., *lsts* – laterosternal s., *pda* – pedal s., *pds* – postdorsal s., *prns* – pronotal s., *prs* – prodorsal s., *ss* – spiracular s., *ps* – pleural s., *sts* – sternal s., *ts* – terminal s., Th1–3 – number of thoracic segments, Ab1–10 – number of abdominal seg.

*Thorax.* Prothorax (Fig. 78) with nine very long and one very short to minute *prns*, small pigmented dorsal sclerite present with seven long *prns*, this sclerite subdivided in two triangular plates medially; two very long to long *ps*; and one medium *eus.* Meso- and metathorax (Fig. 78) with one medium *prs*, three very long *pds*; one very long *as*; two very long and one very short to minute *ss*; one long *eps*; one long *ps*; and one medium to long *eus.* Each pedal area of the thoracic segments with 5–6 very long *pda*.

*Abdomen.* Abdominal segments I–VII (Figs 79–80) with one medium to long *prs*; two medium to short and one very long to long *pds* (order: medium/short, very long, medium); one very long and one minute *ss*; two long *eps*; one medium to short *ps*; one medium to short *lsts*; and one medium to short and one very short to minute *eus.* Abdominal segment VIII (Fig. 80) with one very short *prs*; two short and one long to relatively long *pds* (order: short, long, short); one long and one minute *ss*; two very long *eps*; one medium to short *ps*; one medium to short *lsts*; and one medium to short and one very short to minute *eus.* Abdominal segment IX (Fig. 80) with two relatively long and two short to very short *ds*; two relatively long and sometimes one minute *ps*; and one relatively long to short and one short to very short *sts.* Abdominal segment X (Fig. 80) with one very short seta (*ts*).

###### Biology.

Larvae live and pupate in the capsules of several species of *Campanula* (*C.cochleariifolia* Lam., *C.patula* L., *C.persicifolia* L., *C.rapunculoides* L., *C.rapunculus* L., *C.rotundifolia* L., *C.scheuchzeri* Vill. *C.trachelium* L.), and *Phyteumaspicata* L. where they cause distinct swelling (Hoffmann 1958; Buhr 1964)

###### Remarks.

This species has a wide European distribution and is very similar to *M.ajugae*. Only the examination of the penis allows easy separation of these two taxa. Therefore, as reported in the Remarks of *C.ajugae*, the discovery of clearly distinctive characters between the larvae of these two species is extremely important. Moreover, larvae of *M.campanulae* differ from the two other species studied mainly by an epipharynx without *mes*, and *des_4_* and *fs_3_* absent.

##### Key to larvae (mature larva, L3)

The following key is based on the larvae of five *Cleopomiarus* and three *Miarus* species described in this paper. Unfortunately, the previous description of *Cleopomiarushispidulus* (Anderson 1973) cannot be included due to missing details about the chaetotaxy used in our key.

**Table:** 

1	Mala with 6 finger-like *dms*, all more or less in equal length (Figs 7, 17, 27, 37, 47)	***Cleopomiarus* 2**
–	Mala with 6 finger-like *dms*, in two sizes; 1 or 2 *dms* very long and rest of them in medium length (Figs 57, 67, 77)	***Miarus* 6**
2	*Fs_3_* long, almost as long as *fs_4_* (Fig. 42)	*** C. meridionalis ***
–	*Fs_3_* short or very short, always distinctly shorter than *fs_4_* (Figs 2, 12, 22, 32)	**3**
3	Postlabium with medium size *pms_1_* and *pms_3_*, and very long *pms_2_* (Fig. 7). Membranous area of postlabium basolaterally finely asperate (Fig. 7)	*** C. distinctus ***
–	Postlabium with short *pms_1_* and *pms_3_*, and very long *pms_2_* (Figs 17, 27, 37). Membranous area of postlabium basolaterally sparsely and distinctly asperate (Figs 17, 27, 37)	**4**
4	Antennae bearing one medium size conical sensorium, and 4 sensilla (Fig. 23). Dorsal setae (except *des_4_*) extremely long (Fig. 22). Head width over 1.05 mm. Prothorax with 8 very long and 1 very short to minute *prns* (Fig. 28). Body length over 6.60 mm	*** C. longirostris ***
–	Antennae with very long conical sensorium, and 3 sensilla (Figs 13, 33). Dorsal setae (except *des_4_*) long (Figs 12, 32). Head width under 0.80 mm. Prothorax with 9 very long and 1 very short to minute *prns* (Figs 18, 38). Body length under 6.30 mm	**5**
5	Clypeus (Fig. 35) broad, approximately 4.25 times as wide as long; labrum 2.0 times as wide as long	*** C. medius ***
–	Clypeus (Fig. 15) narrow, 2.5–3 times as wide as long, labrum less than 2 times as wide as long	*** C. graminis ***
6	Mala with 6 finger-like *dms*, different in length: 1 seta very long as *pfs_1_*, and 5 setae in medium length (Fig. 77). Epipharynx (Fig. 76) without *mes. Des_4_* and *fs_3_* absent (Fig. 72)	*** M. campanulae ***
–	Mala with 6 finger-like *dms*, different in length: 2 setae elongated, and 4 setae in medium length (Figs 57, 67). Epipharynx (Figs 56, 66) with 1–2 *mes. Des_4_* and *fs_3_* present (Figs 52, 62)	**7**
7	Epipharynx (Fig. 66) with 2 *mes*, first finger-like, second sharp and slender. Antennae with very long conical sensorium (Fig. 63)	*** M. ajugae ***
–	Epipharynx (Fig. 56) with 1 finger-like *mes.* Antennae bearing medium size conical sensorium (Fig. 53)	*** M. abnormis ***

#### *Description of pupae*

##### Genus Cleopomiarus

Taxon classificationAnimaliaColeopteraCurculionidae

Pierce, 1919

###### Description.

*Measurements* (in mm). Body length: 3.00–6.50. Body width: 1.50–3.80. Head width: 0.65–1.10.

*Body* moderately slender or stout. Smooth, dark brown or black spotted cuticle. Rostrum long or very long, from four up to five times as long as wide, reached up to meso- or metacoxae. Antennae elongated. Pronotum from 2.2 up to 2.9 times as wide as long. Mesonotum distinctly shorter than metanotum. Abdominal segments I–V of equal length; abdominal segments VI and VII diminish gradually; abdominal segment VIII almost semicircle; abdomen segment IX distinctly reduced. Spiracles on abdominal segments placed dorsolaterally: on abdominal segments I–V functional; and on segment VI atrophic on next ones invisible. Urogomphi (ur) stout and short, conical, each of them with sclerotized apex.

*Chaetotaxy*: setae piliform, in a different size. Head capsule with 0–1 *vs*, 0–1 *sos*, and two *os.* Rostrum with 0–1 *pas* and one *rs* (placed medially and apically). Pronotum with: two *as*, one *ds*, 1–2 *sls*, 0–1 *ls* and three *pls.* Setae on head, rostrum and pronotum very thin, light and relatively short. Dorsal parts of meso- and metathorax with 2–3 setae placed medially. Apex of each femora with one *fes.* Abdominal segments I–VIII with two setae laterally and sometimes 2–3 short setae ventrally. Dorsal parts of abdominal segments I–VII with 4–5 setae, and abdominal segment VIII with 3–4 setae dorsally. Abdominal segment IX with 2–4 micro-setae ventrally.

##### Cleopomiarus distinctus

Taxon classificationAnimaliaColeopteraCurculionidae

(Boheman, 1845)

Figures 81 , 82–84

###### Material examined.

12 specimens: 2 ♂; 2 ♀, 03.08.2010, Gródek ad Hrubieszów, CE Poland, leg. E. Szwaj, det. J. Łętowski; 2 ♂; 6 ♀, ex seed capsules of *Campanulacervicaria* L., 05.07.2017, Stara Planina, east Serbia, leg. I. Toševski, det. R. Caldara.

###### Description.

*Measurements* (in mm). Body length: 3.00–4.10 (mean 3.20). Body width: 1.50–2.60 (mean 1.80). Head width: 0.65–0.77 (mean 0.67).

**Figure 81.**
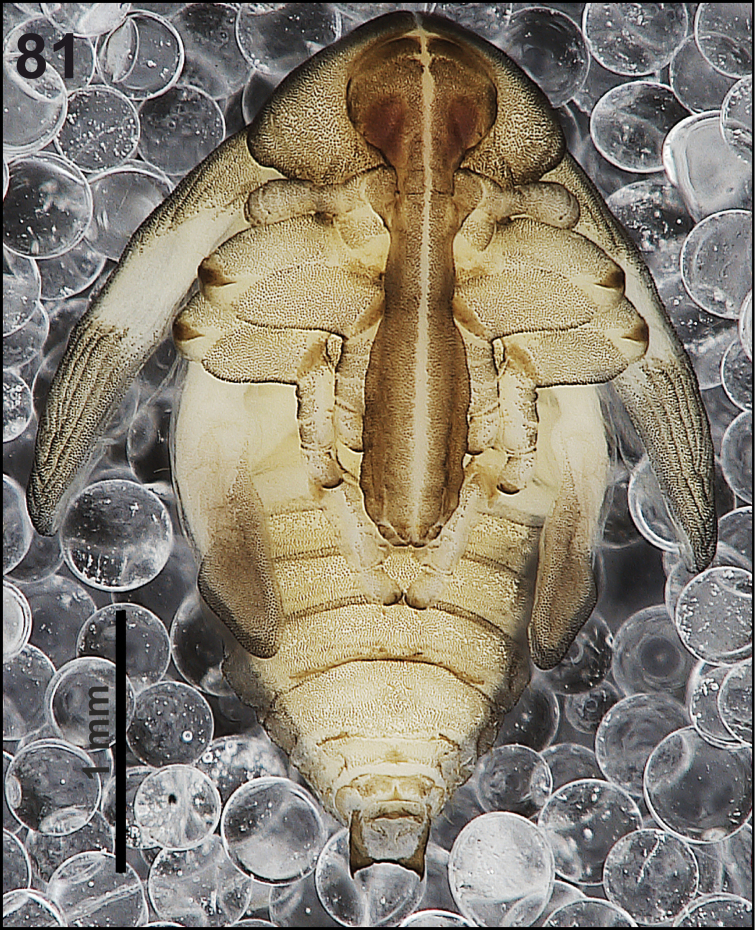
*Cleopomiarusdistinctus* pupa habitus, ventral view.

**Figures 82–84.**
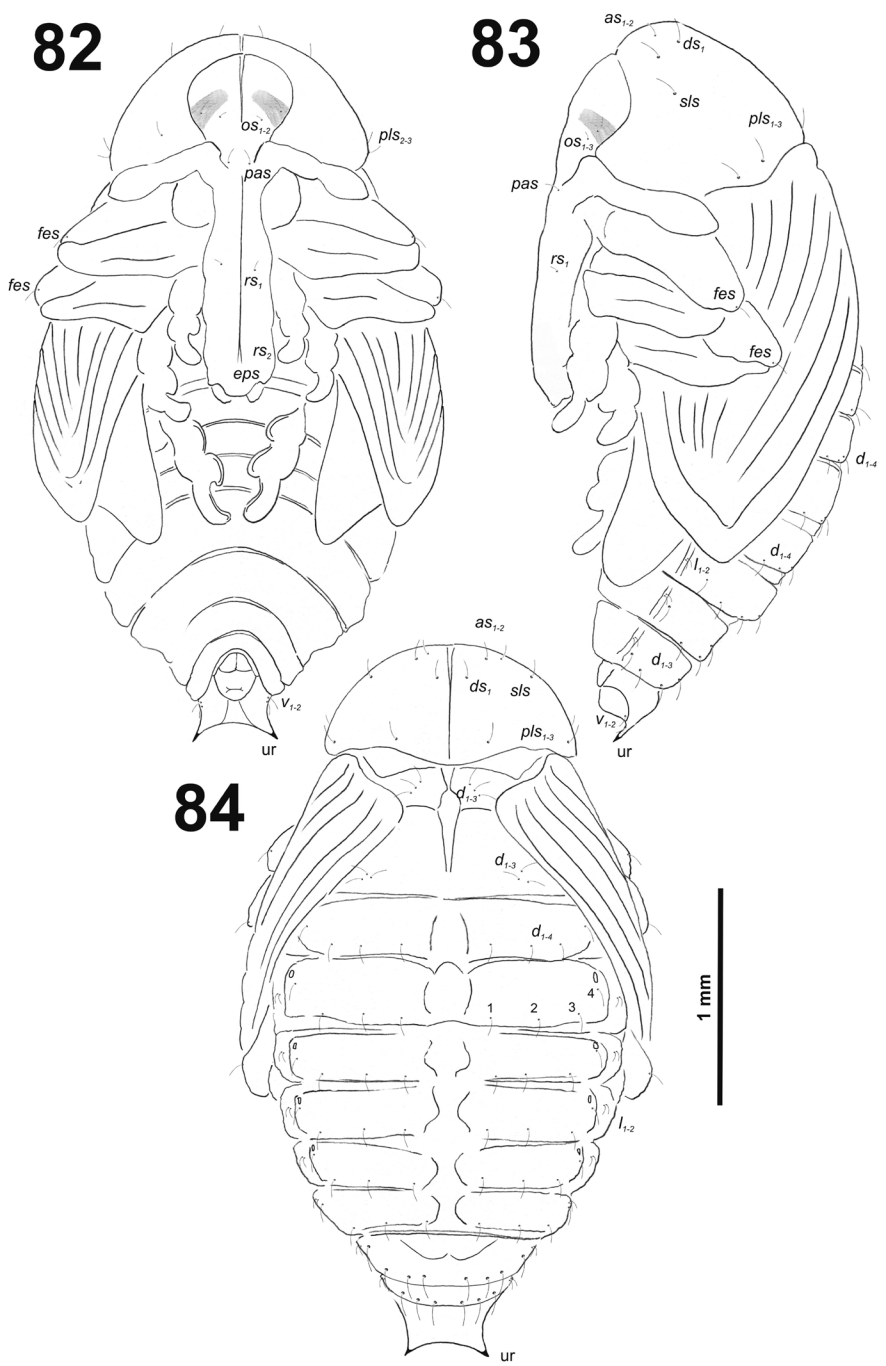
*Cleopomiarusdistinctus* pupa habitus and chaetotaxy. **82** Ventral view **83** Lateral view **84** Dorsal view. Abbreviations: *d* – dorsal s., *ds* – discal s., *fes* – femoral s., *l*, – lateral s., *os* – orbital s., *pas* – postantennal s., *pls* – posterolateral s., *rs* – rostral s., *sls* – super lateral s., *v* – ventral s., ur – urogomphi.

*Body* moderately slender (Figs 81–84). Rostrum long, approximately 4.0 times as long as wide, reached up to mesocoxae. Antennae moderately elongated. Pronotum 2.2 times as wide as long. Urogomphi (ur) stout (Figs 81–84).

*Chaetotaxy*: setae very thin, greyish, piliform, medium size to short. Head capsule with only two *os* of different in length (second pair placed on eye spots). Rostrum with one *pas* and one *rs* (Figs 82, 83). Pronotum with: two *as*, one *ds*, one *sls*, and three *pls* (Figs 83, 84). All setae of prothorax almost equal in length (Fig. 83). Dorsal parts of meso- and metathorax with three setae placed medially. Apex of each femora with one *fes* (Figs 82–84). Abdominal segments I–VIII with two setae laterally. Dorsal parts of abdominal segments I–VII with four setae: *d_1_*–*_3_* placed postero-medially, *d_4_* postero-laterally, abdominal segment VIII with only three setae dorsally. Abdominal segment IX with two micro-setae ventrally.

##### Cleopomiarus graminis

Taxon classificationAnimaliaColeopteraCurculionidae

(Gyllenhal, 1813)

Figures 85 , 86–88

###### Material examined.

15 specimens: 2 ♂, 3 ♀, 27.07.2010, Wólka ad Lublin, CE Poland, leg. E. Szwaj, det. J. Łętowski; 4 ♂, 2 ♀, ex seed pods of *Adenophoraliliifolia*, Dobra, Iron Gate, east Serbia, leg. I. Toševski, det. R. Caldara; 4 ♀, ex seed pods of *Campanulamacrostachya*, 13.07.2015, Dobra, Iron Gate, east Serbia, leg. I. Toševski, det. R. Caldara.

###### Description.

*Measurements* (in mm). Body length: 3.60–4.10 (mean 3.70). Body width: 2.10–2.25 (mean 2.15). Head width: 0.65–0.70 (mean 0.67).

**Figure 85.**
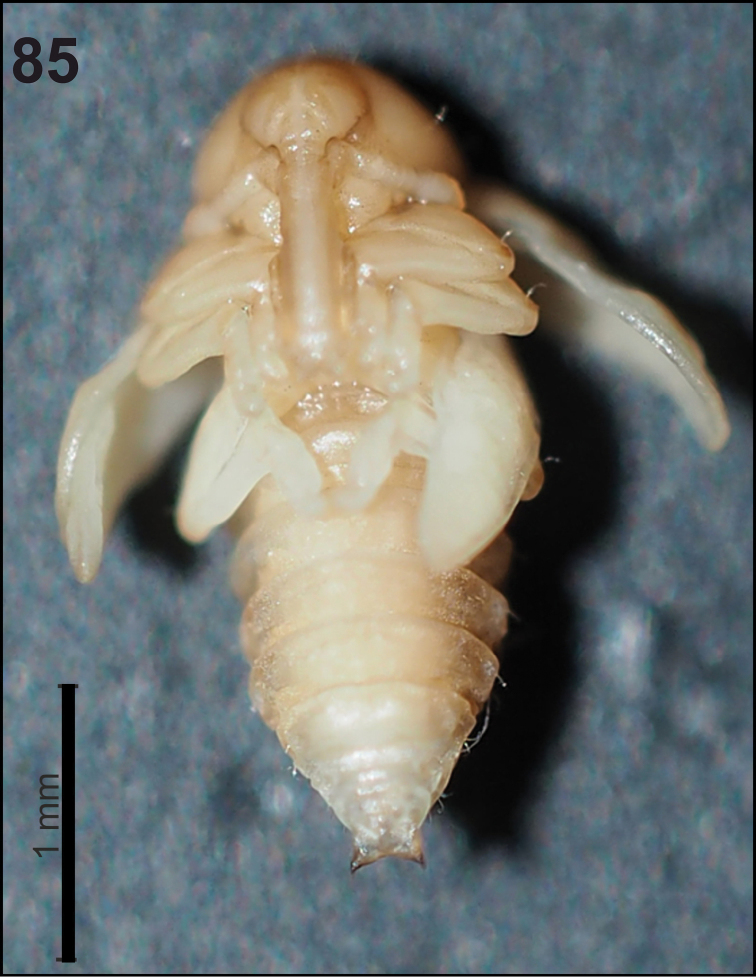
*Cleopomiarusgraminis* pupa habitus, ventral view.

**Figures 86–88.**
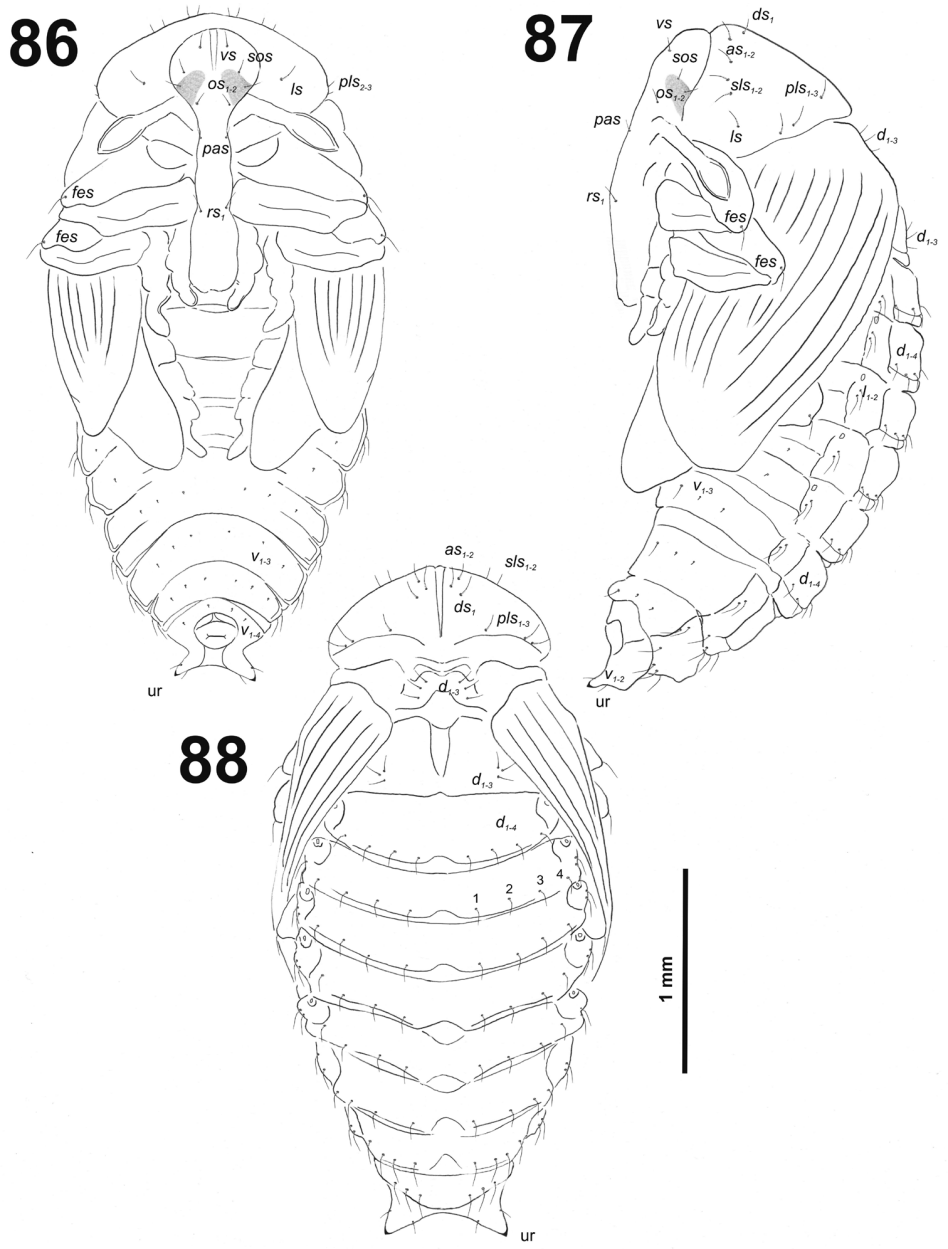
*Cleopomiarusgraminis* pupa habitus. **86** Ventral view **87** Lateral view **88** Dorsal view. Abbreviations: *d* – dorsal s., *ds* – discal s., *fes* – femoral s., *l*, *ls* – lateral s., *os* – orbital s., *pas* – postantennal s., *pls* – posterolateral s., *rs* – rostral s., *sls* – super lateral s., *sos* – super orbital s., *v* – ventral s., *vs* – vertical s., ur – urogomphi.

*Body* moderately slender (Figs 85–88). Rostrum long, approximately four times as long as wide, reached up to mesocoxae. Antennae slender and elongated. Pronotum 2.9 times as wide as long. Urogomphi (ur) moderately stout (Figs 85–88).

*Chaetotaxy*: setae very thin, greyish, piliform, medium size to short. Head capsule with one *vs*, one *sos*, and two *os* of different length (second pair placed on eye spots). Rostrum with one *pas* and one *rs* (Figs 86, 87). Pronotum with: two *as*, one *ds*, two *sls*, one *ls*, and three *pls* (Figs 87, 88). All setae of prothorax equal in length (Fig. 88). Dorsal parts of meso- and metathorax with three setae placed medially. Apex of each femora with one *fes* (Figs 86, 87). Abdominal segments I–VIII with two setae laterally and three micro setae ventrally. Dorsal parts of abdominal segments I–VII with four setae: *d_1_*–*_3_* postero-medially, and *d_4_* postero-laterally. Abdominal segment VIII with four setae dorsally. Abdominal segment IX with four micro-setae ventrally.

##### Cleopomiarus longirostris

Taxon classificationAnimaliaColeopteraCurculionidae

(Gyllenhal, 1838)

Figures 89 , 90–92

###### Material examined.

18 specimens: 8 ♂, 10 ♀, south eastern France, Menton, July 2007, ex seed capsules of *Campanulatrachelium* L., leg. and det. R. Caldara

###### Description.

*Measurements* (in mm). Body length: 5.20–6.50 (mean 5.60). Body width: 3.00–3.80 (mean 3.30). Head width: 0.90–1.10 (mean 1.00).

**Figure 89.**
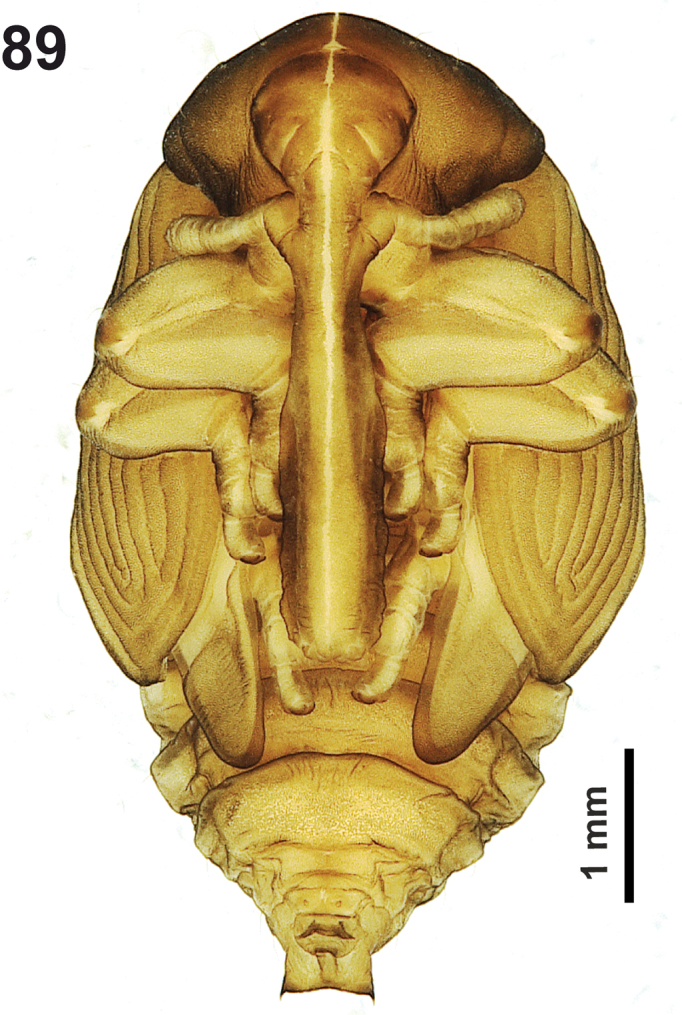
*Cleopomiaruslongirostris* pupa habitus, ventral view.

**Figures 90–92.**
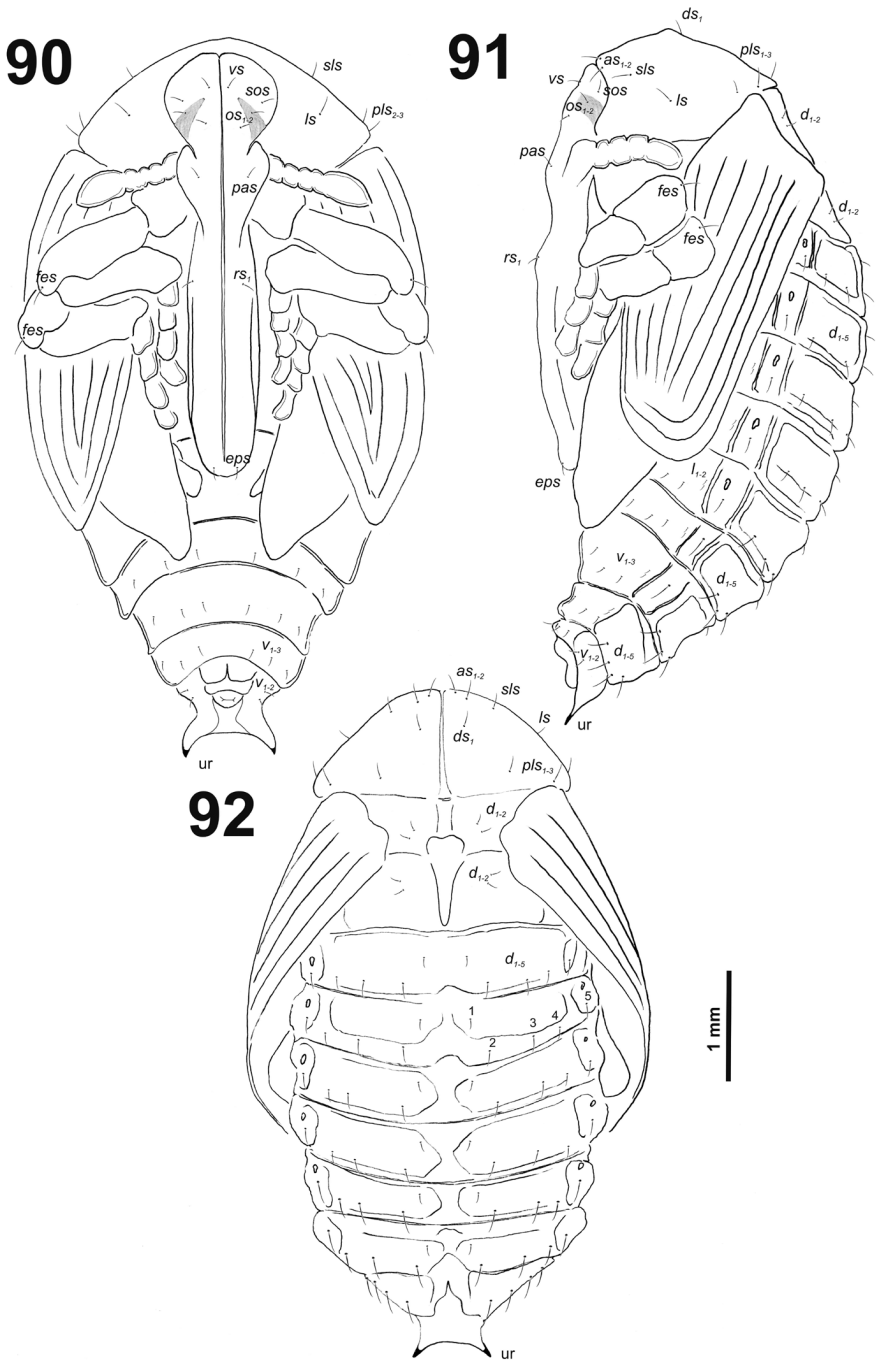
*Cleopomiaruslongirostris* pupa habitus. **90** Ventral view **91** Lateral view **92** Dorsal view. Abbreviations: *d* – dorsal s., *ds* – discal s., *es* –epistomal s., *fes* – femoral s., *l*, *ls* – lateral s., *os* – orbital s., *pas* – postantennal s., *pls* – posterolateral s., *rs* – rostral s., *sls* – super lateral s., *sos* – super orbital s., *v* – ventral s., *vs* – vertical s., ur – urogomphi.

*Body* stout (Figs 89–92). Rostrum very long, almost five times as long as wide, reached almost up to metacoxae. Antennae slender and moderately elongated. Pronotum 2.5 times as wide as long. Urogomphi (ur) short (Figs 90–92).

*Chaetotaxy*: sparse, setae very thin, short, piliform. Head capsule with one *vs*, one *sos*, and two *os* of different length (second pair placed on eye spots). Rostrum with one *rs* and one *es* (Figs 90, 91). Pronotum with: two *as*, one *ds*, one *ls*, one *sls*, and three *pls* (Figs 91, 92). All setae of prothorax equal in length (Fig. 92). Dorsal parts of meso- and metathorax with two setae placed medially. Apex of each femora with one *fes* (Figs 90–92). Abdominal segments I–VIII with two setae laterally and three medium long setae ventrally. Dorsal parts of abdominal segments I–VII with five setae: *d_1_* placed antero-medially, *d_2_*–*_4_* postero-medially, *d_5_* postero-laterally. Abdominal segment VIII with only three setae dorsally. Abdominal segment IX with two micro-setae ventrally.

##### Cleopomiarus medius

Taxon classificationAnimaliaColeopteraCurculionidae

(Desbrochers des Loges, 1893)

Figures 93 , 94–96

###### Material examined.

4 specimens: 2 ♂, 2 ♀, ex seed capsules of *Campanulalingulata* Waldst. and Kit., 26.06.2017, Staničenje, Pirot, east Serbia, leg. I. Toševski, det R. Caldara.

###### Description.

*Measurements* (in mm). Body length: 4.40–4.60. Body width: 2.40–2.50. Head width: 0.70–0.80.

**Figure 93.**
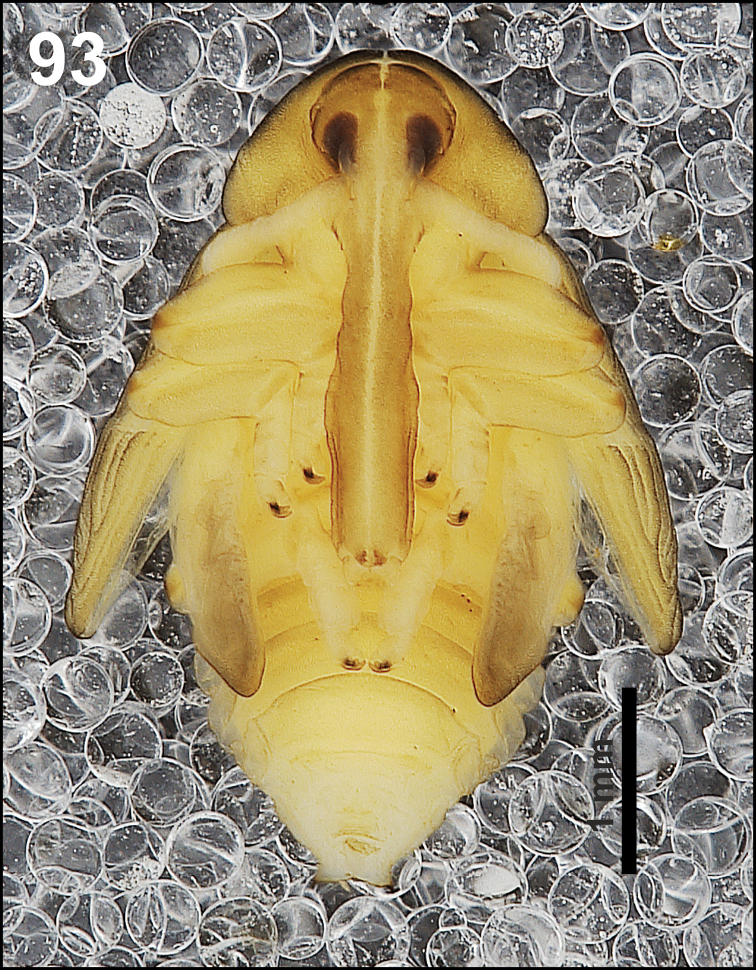
*Cleopomiarusmedius* pupa habitus, ventral view.

**Figures 94–96.**
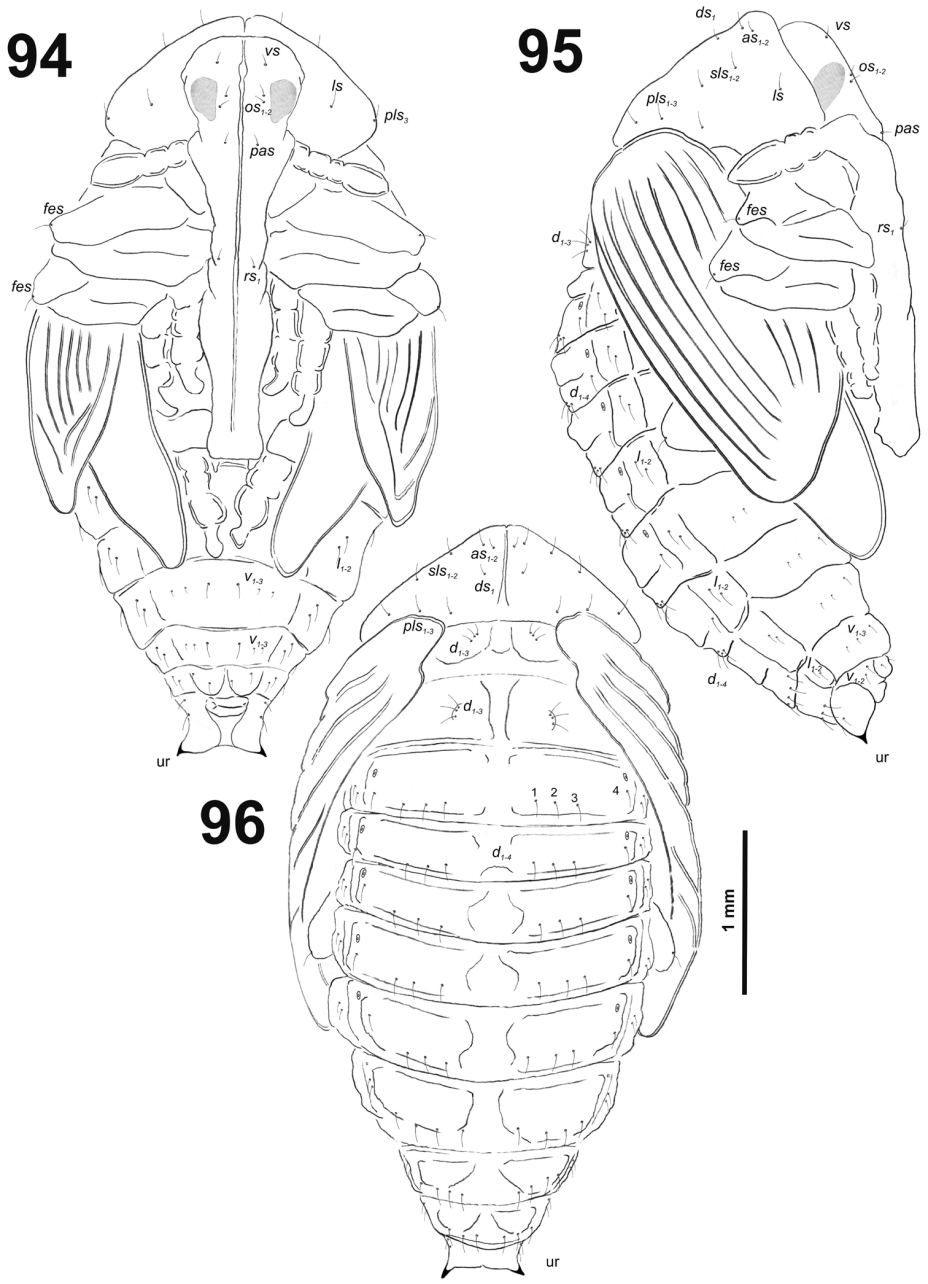
*Cleopomiarusmedius* pupa habitus. **94** Ventral view **95** Lateral view **96** Dorsal view. Abbreviations: *d* – dorsal s., *ds* – discal s., *fes* – femoral s., *l*, *ls* – lateral s., *os* – orbital s., *pas* – postantennal s., *pls* – posterolateral s., *rs* – rostral s., *sls* – super lateral s., *v* – ventral s., *vs* – vertical s., ur – urogomphi.

*Body* moderately stout (Figs 93–96). Rostrum very long, approximately 4.5 times as long as wide, reached beyond mesocoxae. Antennae slender and moderately elongated. Pronotum 2.4 times as wide as long. Urogomphi (ur) stout (Figs 94–96).

*Chaetotaxy*: sparse, setae short to very short, light, piliform. Head capsule with one very short *vs*, and two *os* of different length, both placed between eye spots. Rostrum with one *rs* (Figs 94, 95). Pronotum with: two *as*, one *ds*, two *sls*, one *ls* and three *pls* (Figs 95, 96). All setae of prothorax equal in length (Fig. 96). Dorsal parts of meso- and metathorax with three setae placed medially. Apex of each femora with one *fes* (Figs 94, 95). Abdominal segments I–VIII with two setae laterally and three medium long setae ventrally. Dorsal parts of abdominal segments I–VII with four setae: *d_1_*–*_3_* postero-medially, *d_4_* postero-laterally. Abdominal segment VIII with only three setae dorsally. Abdominal segment IX with four micro-setae ventrally.

##### Genus Miarus

Taxon classificationAnimaliaColeopteraCurculionidae

Schoenherr, 1826

###### Description.

*Measurements* (in mm). Body length: 3.20–4.00. Body width: 1.90–2.70. Head width: 0.55–0.70.

*Body* moderately slender or stout. Smooth, dark brown or black spotted cuticle. Rostrum long or very long, from 2.3 up to 3.5 times as long as wide, reached up to meso- or metacoxae. Antennae elongated. Pronotum from 2.1 up to 2.5 times as wide as long. Mesonotum distinctly shorter than metanotum. Abdominal segments I–V of equal length; abdominal segments VI and VII diminish gradually; abdominal segment VIII almost semicircle; abdomen segment IX distinctly reduced. Spiracles on abdominal segments placed dorsolaterally: on abdominal segments I–V functional; and on segment VI atrophic on next ones invisible. Urogomphi (ur) slender and elongated, conical, each of them with sclerotized apex.

*Chaetotaxy*: setae piliform, in a different size. Head capsule with one *vs*, one *sos*, and 3–4 *os.* Rostrum with one *pas* and three *rs* (placed medially and apically). Pronotum with: two *as*, two *ds*, two *sls*, one *ls* and three *pls.* All setae of prothorax almost equal in length. Dorsal parts of meso- and metathorax with three setae placed medially. Apex of each femora with one *fes.* Abdominal segments I–VIII with two setae laterally and sometimes 3–4 short setae ventrally. Dorsal parts of abdominal segments I–VII with 4–5 setae, and abdominal segment VIII with 3–4 setae dorsally. Abdominal segment IX with 4–6 micro-setae ventrally.

##### Miarus abnormis

Taxon classificationAnimaliaColeopteraCurculionidae

Solari, 1947

Figures 97 , 98–100

###### Material examined.

3 specimens: 1 ♂, 2 ♀, north-eastern Italy, Venezia Giulia, Duino (Trieste), Rilke path, August 2017, ex galls on capsules of *Campanulapyramidalis* L., leg. E. Tomasi, det. R. Caldara.

###### Description.

*Measurements* (in mm). Body length: 3.55–3.60. Body width: 1.90–2.00. Head width: 0.55–0.60.

**Figure 97.**
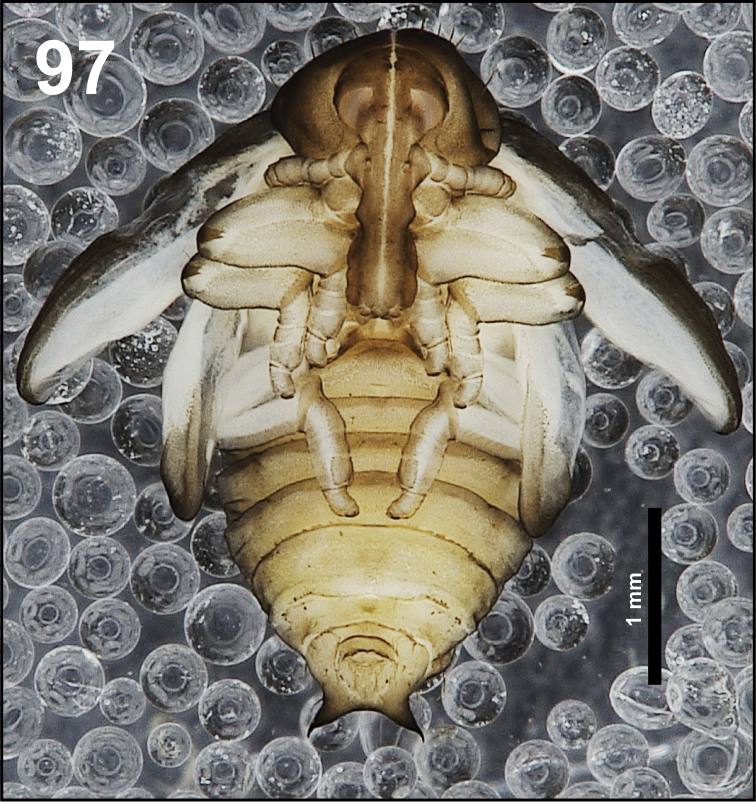
*Miarusabnormis* pupa habitus, ventral view.

**Figures 98–100.**
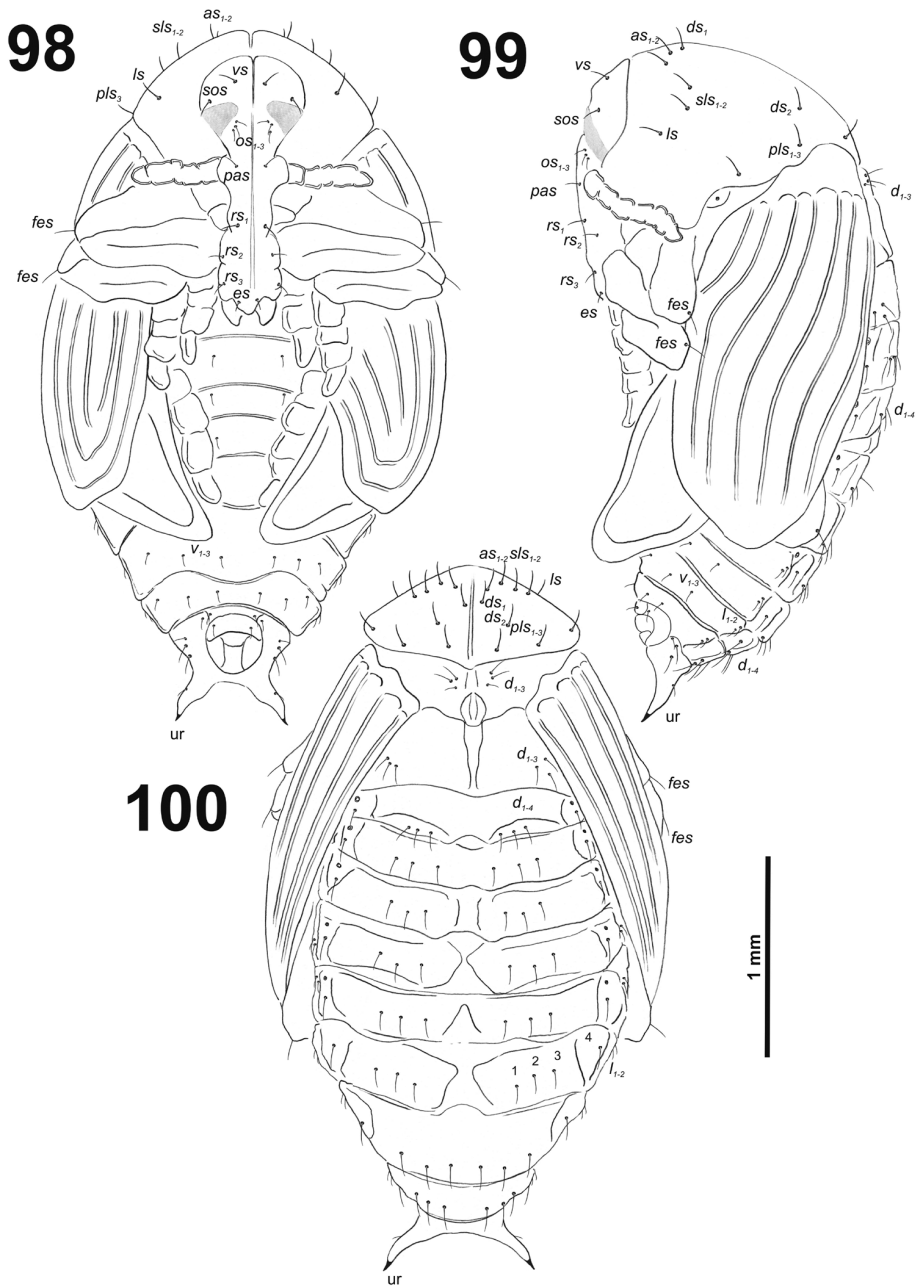
*Miarusabnormis* pupa habitus. **98** Ventral view **99** Lateral view **100** Dorsal view. Abbreviations: *d* – dorsal s., *ds* – discal s., *es* – epistomal s., *fes* – femoral s., *l*, *ls* – lateral s., *os* – orbital s., *pas* – postantennal s., *pls* – posterolateral s., *rs* – rostral s., *sls* – super lateral s., *sos* – super orbital s., *v* – ventral s., *vs* – vertical s., ur – urogomphi.

*Body* moderately slender (Figs 97–100). The cuticle densely covered with asperities. Rostrum medium length, approximately 2.3 times as long as wide, reached up to mesocoxae. Antennae moderately elongated. Pronotum 2.1 times as wide as long. Urogomphi (ur) slender and elongated, conical, each of them with sclerotized apex (Figs 98–100).

*Chaetotaxy*: setae brownish to dark brown, piliform, short to medium size. Head capsule with one *vs*, one *sos*, and three *os* of equal length; rostrum with three *rs* (*rs_1_* and *rs_2_* placed medially, *rs_3_* more apically) and one *es* (Figs 98, 99). Pronotum with: two *as*, two *ds*, two *sls*, one *ls* and three *pls* (Figs 99. 100). Setae on head and rostrum slightly shorter than those on prothorax. All setae of prothorax almost equal in length (Fig. 100). Mesothorax with three setae placed medially; metathorax with three setae placed laterally. Apex of each femora with one *fes* (Figs 98–100). Abdominal segments I–VIII with two short, thin setae laterally and three short setae ventrally. Dorsal parts of each abdominal segments I–VIII with four setae: *d_1_*–*_3_* placed postero-medially, *d_4_* postero-laterally. Abdominal segment IX with six microsetae ventrally.

##### Miarus ajugae

Taxon classificationAnimaliaColeopteraCurculionidae

(Herbst, 1795)

**Figure 101.**
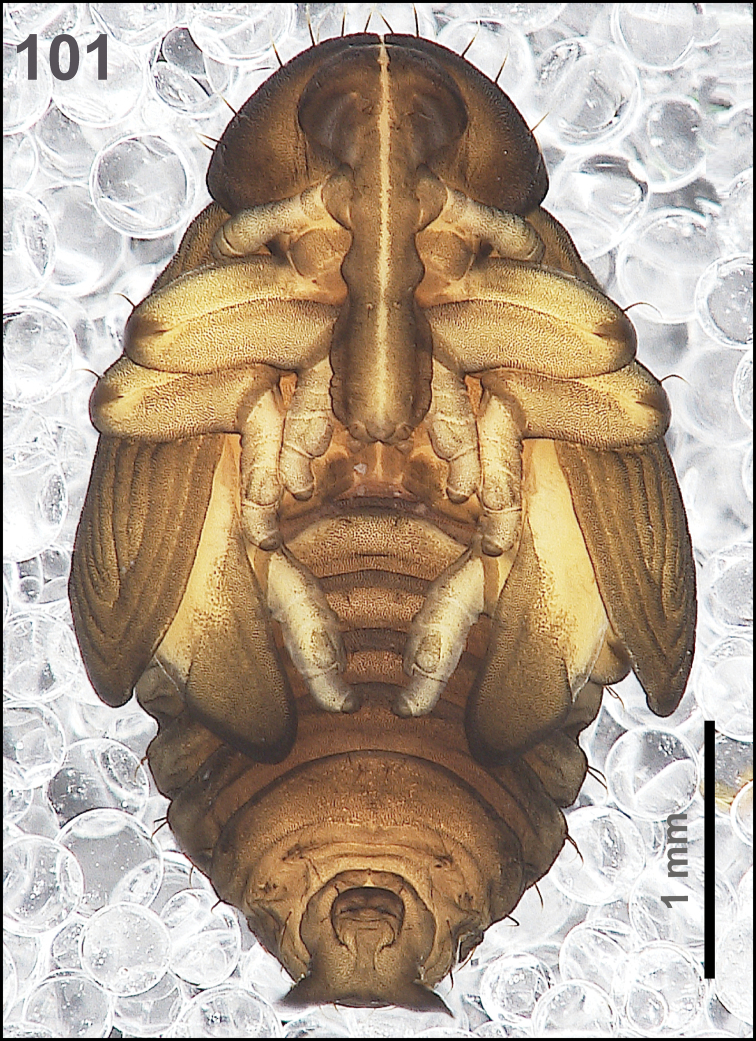
*Miarusajugae* pupa habitus, ventral view.

**Figures 102–104.**
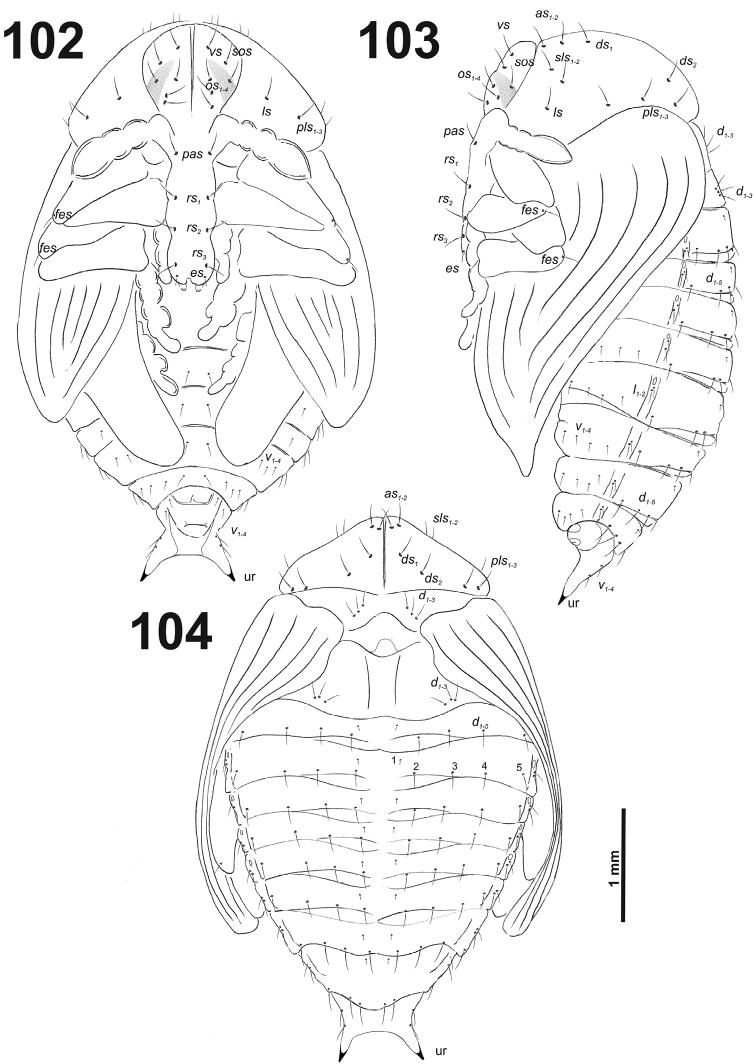
*Miarusajugae* pupa habitus. **102** Ventral view **103** Lateral view **104** Dorsal view. Abbreviations: *d* – dorsal s., *ds* – discal s., *es* – epistomal s., *fes* – femoral s., *l*, *ls* – lateral s., *os* – orbital s., *pas* – postantennal s., *pls* – posterolateral s., *rs* – rostral s., *sls* – super lateral s., *sos* – super orbital s., *v* – ventral s., *vs* – vertical s., ur – urogomphi.

Figures 101 , 102–104

###### Material examined.

12 specimens: 2 ♂, 1 ♀, 24.08.2009, Ciechanki, CE Poland, leg. E. Szwaj, det. J. Łętowski; 3 ♂, 2 ♀, 30.06.2017, ex galls on capsules of *Adenophoraliliifolia*, 30.06.2017, Kaludjerske Bare, Mt. Tara, Central Serbia, leg. I. Toševski, det. R. Caldara; 3 ♂, 1 ♀, ex galls on capsules of *Campanulabononiensis*, 14.07.2017, Zavojsko jezero, Pirot, east Serbia, leg. I. Toševski, det. R. Caldara.

###### Description.

*Measurements* (in mm). Body length: 3.20–4.00 (mean 3.40). Body width: 1.90–2.70 (mean 2.10). Head width: 0.65–0.70 (mean 0.65).

*Body* rather stout (Figs 101–104). Rostrum moderately elongated, approximately 3.5 times as long as wide, reached almost up to metacoxae. Antennae rather stout and moderately elongated. Pronotum 2.5 times as wide as long. Urogomphi (ur) slender and elongated (Figs 102–104).

*Chaetotaxy*: setae piliform, greyish to dark brown, different in length, short to medium size. Head capsule with one *vs*, one *sos*, and four *os* of different length (fourth placed on eye spots). Rostrum with one *pas*, and three *rs* (*rs_1_* and *rs_2_* placed medially, *rs_3_* more apically) and one *es* (Figs 102, 103). Pronotum with: two *as*, two *ds*, one *ls*, two *sls*, and three *pls* (Figs 103, 104). All setae of prothorax equal in length (Fig. 104). Dorsal parts of meso- and metathorax with three setae placed medially. Apex of each femora with one *fes* (Figs 102, 103). Abdominal segments I–VIII with two setae laterally and four medium long setae ventrally. Dorsal parts of abdominal segments I–VII with five setae: *d_1_* placed antero-medially, *d_2_*–*_4_* postero-medially, *d_5_* postero-laterally, and abdominal segment VIII with only three setae dorsally. Abdominal segment IX with four micro-setae ventrally.

##### Key to pupae

The following key is based on pupae of four *Cleopomiarus* and two *Miarus* species described in this paper. Unfortunately, the description of *Cleopomiarushispidulus* published previously (Anderson 1973) could not be included due to missing details about the chaetotaxy used in our key.

**Table:** 

1	Rostrum with 1 *rs* (Figs 82, 86, 90, 94). Setae on head, rostrum and pronotum very thin, light and relatively short (Figs 83, 87, 91, 95). Pronotum with 1 *ds* (Figs 84, 88, 92, 96). Urogomphi stout and short (Figs 82, 86, 90, 94)	**2 *Cleopomiarus***
–	Rostrum with 3 *rs* (Figs 98, 102). Setae on head, rostrum and pronotum brown, prominent and relatively long (Figs 99, 103). Pronotum with 2 *ds* (Figs 100, 104). Urogomphi relatively slender and elongated (Figs 100, 104)	**5 *Miarus***
2	Rostrum extremely elongated (at least 4.5 times as long as wide) (Figs 89, 93). Head with 2 *os* (Figs 90, 94)	**3**
–	Rostrum elongated (ca. 4.0 times as long as wide) (Figs 81, 85). Head with 3 *os* (Figs 82, 86)	**4**
3	Body length over 5.20 mm; head width over 0.90 mm. Pronotum with 1 *sls*; *es* present (Fig. 91)	*** Cleopomiarus longirostris ***
–	Body length under 4.60 mm; head width under 0.80 mm. Pronotum with 2 *sls*; *es* absent (Fig. 95)	*** C. medius ***
4	*Vs* and *sos* present (as long as other setae on head) (Fig. 86). Pronotum with 2 *sls* (Fig. 87). Abdominal segments I–VII with 3 ventral setae (Fig. 87)	*** C. graminis ***
–	*Vs* and *sos* absent (or as microsetae) (Fig. 82). Pronotum with 1 *sls* (Fig. 83). Abdominal segments I–VII without ventral setae (Fig. 82)	*** C. distinctus ***
5	Body length usually over 3.60 mm (Fig. 101). Head width over 0.65 mm. Head with 4 *os* (Fig. 102)	*** Miarus ajugae ***
–	Body length usually under 3.50 mm (Fig. 97). Head width under 0.60 mm. Head with 3 *os* (Fig. 98)	*** M. abnormis ***

## Discussion

### Comparison with immature stages of known Mecinini

Presently, it is somewhat difficult to compare the immatures of *Cleopomiarus* and *Miarus*, which we have just described, with those of other genera of the Mecinini. As above reported, the description of most of the 19 species previously described is somewhat problematic because of missing details about the chaetotaxy and/or absence of quality drawings. Only the recent descriptions of three species of *Gymnetron* (Jiang and Zhang 2015) and one species of *Rhinusa* (Gosik 2010) are useful for a comparison with our data, although some categorizations of setae in the former paper are very disputable (e.g., thoracic and abdominal dorsal setae) and create an unfounded differential diagnosis, precluding the construction of a key to the tribe. Having said this, we think that there are important but still disputable character states within the tribe Mecinini.

The most important morphological character of larvae in this tribe is probably the count of palpomeres on the labial palpi. The basal state in weevils is the presence of two palpomeres on labial palpi (Marvaldi 1997), but it is known that *Gymnetron* species has only one palpomere (May 1993; Jiang and Zhang 2015). The count of these palpomeres in some descriptions in this paper, mainly in *Cleopomiarus*, is disputable. There is not a distinct separation of basal palpomere from the labium, which can appear to be only one palpomere. This state in *Cleopomiarus* and partially in *Miarus* could be an intermediate stage in the reduction in the *Gymnetron* species; however, this should be compared with other Mecinini genera such as *Rhinusa* and *Mecinus* and discussed only within the whole tribe.

Another crucial genus-specific character in Mecinini larvae is the state of the thoracic and abdominal spiracles. All known larvae of *Cleopomiarus* and *Miarus* species have bicameral spiracles on the thorax and abdomen, but other groups within tribe have only unicameral spiracles (*Gymnetron* species; Jiang and Zhang 2015) or thoracic spiracle bicameral and abdominal spiracles unicameral (some *Rhinusa* and *Mecinus*; Anderson 1973; May 1993).

The count of some setae on the epipharynx (especially *ams* and *mes*) in Curculionidae has not been completely resolved, but this has been discussed in previous papers (e.g., Tychiini: Skuhrovec et al. 2014, 2015; Gosik et al. 2017). According to Marvaldi (1998, 1999), the standard status of the epipharyngeal setae in weevils is two *ams* and three *mes*, but when the position of the distal *mes* is very close to the anterior margin, it appears as *ams.* There are also situations with some out of line positions of the *als* where the position is very close to the tormae (then it appears to be a *mes*) or close to the *ams.* The final decision about the count of each seta is important, but not crucial, and the comparison between groups/genera should be done together for all three of these epipharyngeal setae in order to make fewer mistakes in the creation of a differential diagnosis of genera in the tribe.

The last observed important characteristic within the Mecinini tribe is the vestiture of the body with distinct asperities; however, this feature can be variable within each genus due to specific environmental conditions within plant tissues. This feature will possibly be discussed after other detailed descriptions within the Mecinini tribe.

### Comparison of the immature stages of *Cleopomiarus* and *Miarus*

Before this study, larvae of only two *Cleopomiarus* species (*C.graminis* and *C.hispidulus*) and one *Miarus* species (*M.campanulae*) had been described (Emden 1938; Scherf 1964; Anderson 1973), while a description of the pupae was available for *C.hispidulus* (Anderson 1973). Therefore, a detailed redescription of the two species, *Cleopomiarusgraminis* and *Miaruscampanulae*, has been necessary for their incorporation into the key. The previous descriptions were almost without the chaetotaxy with a few exceptions (e.g., number of *ams* and *als* in *C.graminis*) and included only basal morphological descriptions such as the number of teeth on the mandible or colouration of the head and body. The present detailed descriptions of the immature stages of five *Cleopomiarus* and three *Miarus* species allow a comparison of both genera.

The comparative study of these immatures supports the theory that these genera may be monophyletic based on several unique characteristics (see below), as already suggested in a phylogenetic study by Caldara (2001) on the basis of the adult morphological characters. Larvae of *Miarus* have mala with six finger-like *dms* in two sizes: one or two very long *dms* with the rest being medium length. This appears to be a unique characteristic in weevils. More genus-specific characters are in the pupae, which are more conservative in chaetotaxy. The main differential characters in pupae among known species include the following: (1) setae on the head, rostrum and pronotum very thin, light and relatively short (*Cleopomiarus*) vs brown, prominent and relatively long (*Miarus*); (2) rostrum with one *rs* (*Cleopomiarus*) vs rostrum with three *rs* (*Miarus*); (3) pronotum with one *ds* (*Cleopomiarus*) vs pronotum with two *ds* (*Miarus*); and finally (4) urogomphi (ur) stout and short (*Cleopomiarus*) vs relatively slender and elongated (*Miarus*). Less genus-specific character states in the larvae than in pupae were also shown in another tribe of the Curculioninae (Tychiini) with regard to genera *Tychius* Germar, 1817 and *Sibinia* Germar, 1817 (see Skuhrovec et al. 2014, 2015).

### Differences between immatures at the species level

Our study shows that all the species considered can be identified by the examination of larvae and pupae based on at least one character state. It is noteworthy that several taxa examined only by the study of imagoes were difficult to separate. Therefore, finding distinctive supplementary characters is welcome. This is true for *C.longirostris* vs *C.graminis* and especially *M.ajugae* vs *M.campanulae*. The latter case is particularly emblematic. In these two taxa the adults may be separated by the shape of the apical part of the penis. On the contrary they seem indistinguishable on the basis of barcoding (Vahtera and Muona 2006; Hendrich et al. 2015).

### Importance of molecular data

We have confirmed that a molecular study of immatures is very important in cases where it is necessary to be sure on the identity of a species as already demonstrated by Horecka et al. (2017) just for *Miarus* and *Cleopomiarus*. This is true especially when it is known that more than one related species can live on the same plant or when imagoes are not available or finally when one has to study specimens not personally collected and preserved in a museum. It is noteworthy that the data filed on GenBank or BOLD are becoming larger and larger and therefore more and more useful for such an adequate comparison. Therefore, it is also important to continue to implement these data. In this regard, it is noteworthy that we reported the barcode of *C.medius* for the first time.

### Biological considerations

It is obvious that only a careful search of immature and a careful study of their biological cycle can distinguish true host plants from those used only as a refuge or adult food when the host plants are not yet or no longer available. Our observations confirm that the species of both genera *Cleopomiarus* and *Miarus* are monophagous, although never strictly monophagous, to oligophagous (Bernays and Chapman 1994). Moreover, our data show that the larvae of *Miarus* are gall-inducers as previously reported (Buhr 1964; Tomasi 2002) but that the *Cleopomiarus* species do not form galls. However, this apparently different biological behaviour requires confirmation, since it is well known that several closely related species of Mecinini, especially in the genera *Gymnetron* and *Rhinusa*, do not have the same behaviour with regard to induction of galls (Caldara 2008; Caldara et al. 2010).

## Conclusions

Our data show that detailed descriptions of the immature stages of the Mecinini species are important for further studies of generic taxonomic relationships within the Mecinini group. The detailed descriptions of larvae and pupae of five *Cleopomiarus* and three *Miarus* species are reported in this study. Although the number remains low in comparison with the total number of species of *Cleopomiarus* and *Miarus*, these results demonstrate the possibility of identifying the immature stages in these species, as was done in other groups of weevils (see Skuhrovec et al. 2014, 2015). This is our first paper about the Mecinini group. Detailed descriptions of the genera *Gymnetron*, *Rhinusa*, and *Mecinus* and a tribe overview will be published soon.

## Supplementary Material

XML Treatment for Cleopomiarus

XML Treatment for Cleopomiarus distinctus

XML Treatment for Cleopomiarus graminis

XML Treatment for Cleopomiarus longirostris

XML Treatment for Cleopomiarus medius

XML Treatment for Cleopomiarus meridionalis

XML Treatment for Miarus

XML Treatment for Miarus abnormis

XML Treatment for Miarus ajugae

XML Treatment for Miarus campanulae

XML Treatment for Cleopomiarus

XML Treatment for Cleopomiarus distinctus

XML Treatment for Cleopomiarus graminis

XML Treatment for Cleopomiarus longirostris

XML Treatment for Cleopomiarus medius

XML Treatment for Miarus

XML Treatment for Miarus abnormis

XML Treatment for Miarus ajugae
